# Anthranilamide-protected vinylboronic acid: rational monomer design for improved polymerization/transformation ability providing access to conventionally inaccessible copolymers†

**DOI:** 10.1039/d2sc05094c

**Published:** 2022-10-18

**Authors:** Hiroshi Suzuki, Tsuyoshi Nishikawa, Hiroshi Makino, Makoto Ouchi

**Affiliations:** Department of Polymer Chemistry, Graduate School of Engineering, Kyoto University Nishikyo-ku Kyoto 606-8501 Japan nishikawa.tsuyoshi.8n@kyoto-u.ac.jp ouchi.makoto.2v@kyoto-u.ac.jp

## Abstract

We have studied several protecting groups for vinylboronic-acid derivatives as monomers in radical polymerizations with the objective to improve the polymerization ability and C–B bond-cleaving post-transformation performance. Anthranilamide (aam)-protected vinylboronic acid (VBaam) exhibited experimentally a relatively high polymerization activity, which was theoretically corroborated by density functional theory (DFT) calculations that revealed a peculiar effect of the interaction between the aam groups on the polymerization behavior. The VBaam units in the copolymers can subsequently be transformed into vinyl alcohols or into ethylene units through C–B-bond-cleaving side-chain replacement, which affords valuable copolymers such as poly(vinyl alcohol-*co*-styrene), poly(ethylene-*co*-styrene), and poly(ethylene-*co*-acrylate).

## Abstract 2

We designed a vinyl-boronic-acid protected by anthranilamide as a “transformable” monomer in radical polymerization to synthesize conventionally inaccessible copolymers, such as poly(vinyl alcohol-*co*-styrene) and poly(ethylene-*co*-acrylate).

## Graphical abstract

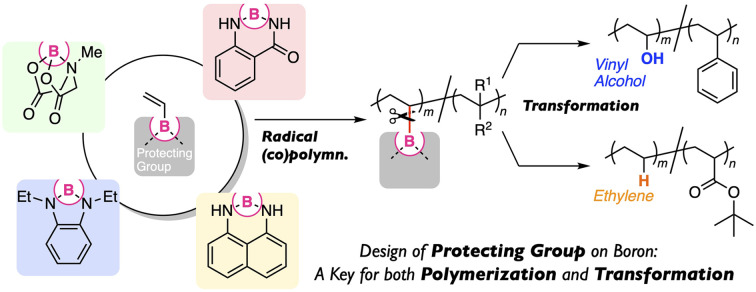

## Introduction

**Fig. 1:**
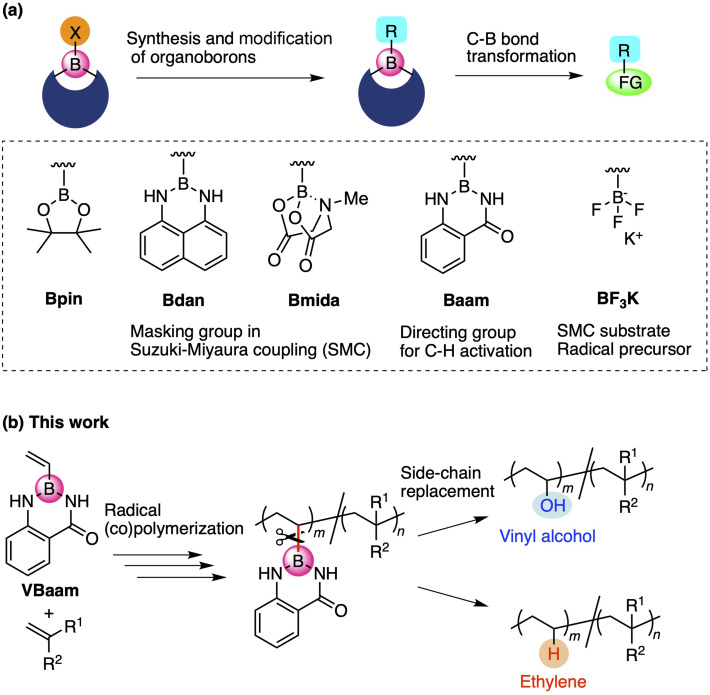
(a) Design of protecting groups for organoborons and their synthetic utility. (b) Vinyl monomer bearing an aam-protected boron for the versatile synthesis of copolymer *via* C–B bond-cleaving side-chain replacement.

Organoboron compounds occupy an important position in contemporary synthetic chemistry due to their unique functionality, reactivity, and transformability. Boron forms stable covalent bonds with carbon atoms due to its relatively high electronegativity, while its vacant p-orbital allows the activation of C–B bonds by nucleophiles, leading to versatile molecular transformations.^1^ One of the reasons for the high synthetic utility of organoboron compounds is the ability to tune their reactivity and transformability based on the choice of the boron protecting group (Fig. 1a).^2^ For instance, this protecting-group-dependent reactivity enables the precise synthesis of oligoarenes *via* iterative Suzuki–Miyaura cross-coupling (SMC) reactions *via* a detachable masking group such as 1,8-diaminonaphthalene (dan) or *N*-methyliminodiacetic acid (mida).^3,4^ Anthranilamide (aam) is another class of masking group for such coupling reactions; Suginome and co-workers have reported that aam-protected boron moieties act as a directing group for *ortho*-selective C–H-activation reactions due to the affinity of the amide group toward transition-metal complexes.^5^ Trifluoroborates, *i.e.*, anionic organoborons that bear three fluorine atoms, are also useful as cross-coupling substrates and carbon-radical precursors.^6^ The use of such boron-based synthetic chemistry can be expected to broaden the scope of synthetically accessible molecules.

In the chain-growth polymerization of vinyl monomers, the element attached to the vinyl moiety and/or the substituent of the pendant crucially determine the polymerization ability.^7^ For example, vinyl ether is a monomer with an alkoxy (OR) side chain on the vinyl group. This monomer is suitable for cationic polymerizations rather than anionic or radical polymerizations due to its non-conjugated structure and electron-richness, although recent studies have shown that it is also able to engage in radical polymerizations.^8^*N*- or *S*-vinyl monomers such as *N*-vinyl pyrrolidone and vinyl phenyl sulfide can be used for radical homopolymerizations, and the resulting polymers have attracted attention due to their heteroatom-oriented optoelectronic functions.^9,10^ These vinyl monomers with attached heteroatoms belong to the class of non-conjugated or less-activated monomers, and usually form less-stabilized chain-growth radical species. The compatibility of monomers in copolymerizations is also critically affected by the monomer character, which depends on the side chain moieties. For example, it is hard to synthesize poly(vinyl alcohol-*co*-styrene) due to the poor copolymerization ability of vinyl acetate and styrene.^11^

Recently, we have revealed the radical-polymerization ability of isopropenyl and vinylboronic acid pinacol ester (IPBpin and VBpin), in which the boron atom is directly attached to the olefin moiety.^12^ In contrast to other vinyl monomers that contain directly connected heteroatoms such as oxygen, nitrogen, or sulfide, alkenyl boronates show conjugated-type monomer character due to the moderate stabilization of the growing carbon-radical species on account of the vacant p-orbital on boron. Furthermore, the boronyl substituents on the resulting polymer backbone can be replaced with other pendants *via* post-polymerization modifications (PPMs). The PPM concept has attracted attention as a technique for varying the properties and functions of polymers,^13^ and the replacement of boronyl side chains is quite unique because of the possibility for the introduction of various side-chain elements to overcome the synthetic limitations in chain-growth polymerizations.^14^ For example, post-polymerization oxidation of boron pendants allowed the preparation of poly(α-methyl vinyl alcohol) and poly(vinyl alcohol-*co*-styrene), which are difficult to synthesize due to the poor (co)polymerization ability of the precursor monomers α-methyl vinyl acetate and vinyl acetate. Thus, boron-containing monomers may provide access to novel routes to polymers that overcome the synthetic limitations of hitherto reported polymerization strategies. Moreover, the synthetic utility of vinyl azaborines as another class of boron monomers has been reported by Jäkle,^15^ Staubitz,^16^ and Klausen.^17^ Azaborine-type monomers exhibit styrene-like polymerization abilities, and the side chain can be oxidized to obtain a vinyl alcohol unit. In the context of these pioneering investigations of boron-based monomers and the structure-dependent reactivity of boron reagents in organic chemistry, we focused on the design of monomers that contain a boronic acid ester and amide protecting groups to broaden the synthetic targets in polymer reactions. We found that aam-protected vinylboronic acid (VBaam) was a suitable monomer for radical (co)polymerizations and that its activity was higher than that of VBpin. The C–B bond of the VBaam unit was more susceptible to transformations than that of VBpin, which allowed synthesizing valuable copolymers, such as poly(vinyl alcohol-*co*-styrene), poly(ethylene-*co*-styrene), and poly(ethylene-*co*-acrylate) (Fig. 1b). Synthesis of these copolymers is not straightforward due to poor compatibility of the corresponding monomers for radical copolymerization.

## Results and discussion

**Fig. 2:**
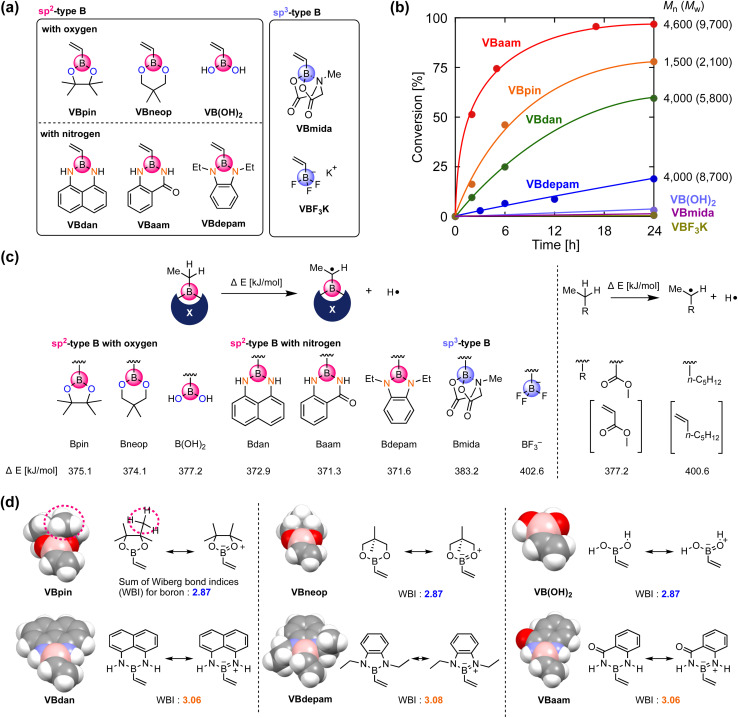
Evaluation of the radical-polymerization behavior of vinylboron derivatives: (a) structure of the boron-containing monomers. (b) Time–conversion plot for the radical polymerization of the vinylboron derivatives. (c) DFT-based evaluation of the radical-stabilization effect of the boron pendants. (d) Space-filling model for each of the optimized vinylboron derivatives and the sum of the WBI around the boron atom; calculated at the (U)B3LYP/6-31G(d) level.

We synthesized vinylboronic acid derivatives that carry different protecting groups on the boron atom and investigated their ability to serve as monomers for radical polymerizations (Fig. 2a, Schemes S1 and S2, Fig. S1–S4†). Vinylboronic acid pinacol ester (VBpin), neopentyl-glycol-protected vinylboronic acid (VBneop), and free vinylboronic acid [VB(OH)_2_] are categorized as oxygen-connected boron compounds, whereas diaminonaphthalene (VBdan), anthranilamide (VBaam), and *N*,*N*′-diethyl-*o*-phenylene diamine (VBdepam) are classified as nitrogen-connected boron compounds. In contrast to the B(sp^2^)-type motif of these compounds, methyliminodiacetic acid (VBmida) and trifluoroborate (VBF_3_K) are B(sp^3^)-type derivatives.

An excess of these compounds was stirred with 2,2′-azobisisobutyronitrile (AIBN) at 60 °C in THF to examine their radical polymerization ability (Table S1†). Fig. 2b shows the time–conversion plots and molecular weights of the products after 24 h. The consumption rates of VBdepam and VBdan were lower than that of VBpin, and the number-average molecular weight (*M*_n_) of the products (after 24 h), which was measured by size-exclusion chromatography (SEC; PMMA calibration; Fig. S5a–c†), was higher. In contrast, the nitrogen-connected derivative VBaam allowed a faster polymerization and furnished a polymer with relatively high *M*_n_ (*M*_n_ = 4.6 × 10^3^; Fig. S5d†), albeit that the polymer partially precipitated during the polymerization, which is most likely due to interactions between the aam groups (*vide infra*). In the case of VB(OH)_2_ and B(sp^3^)-type derivatives (VBmida and VBF_3_K), polymerization did not take place (DMF was used as the solvent instead of THF; Fig. S6†). During the polymerization of VBneop, an insoluble precipitate formed, and the product was not soluble in common solvents. Thus, the polymerization behavior varied greatly depending on the substituent on boron. To understand any underlying trends in the polymerization ability, we used DFT calculations to evaluate the effect of the boron protecting groups on the stability of the chain-growth radicals using the dissociation energy (Δ*E*) of the α-C–H group in the ethylboronic-acid derivatives that correspond to the saturated monomers (Fig. 2c). For reference purposes, we also performed the same calculations for those compounds that correspond to methyl acrylate (MA) and 1-heptene as typical conjugated and non-conjugated monomers. The non-polymerizable monomers that contain an sp^3^-type boron (Bmida and BF_3_^−^) showed relatively high Δ*E* values (Bmida: 383.2 kJ mol^−1^; BF_3_^−^: 402.6 kJ mol^−1^). In these monomers, the boron moiety cannot stabilize the neighboring carbon radical due to its non-conjugated structure, which was supported by the comparable value obtained for the typical unconjugated monomer 1-heptene (400.6 kJ mol^−1^). The values for B(OH)_2_ (377.2 kJ mol^−1^) and Bneop (374.1 kJ mol^−1^) were lower than and almost equal to those of Bpin (375.1 kJ mol^−1^) and MA (377.2 kJ mol^−1^). The vacant p-orbital in boron can contribute to the stabilization of adjacent radicals, and thus, they can be categorized as conjugated monomers. The B–N-bond-containing vinylboron compounds also furnished similar Δ*E* values (Bdan: 372.9 kJ mol^−1^; Baam: 371.3 kJ mol^−1^; Bdepam: 371.6 kJ mol^−1^), suggesting that a similar radical-stabilizing effect can be expected. However, the values were only a little lower than those of the B–O counterparts. The p-orbital of boron is probably occupied by the lone pair of nitrogen attached to boron, and the antibonding π orbital of the B–N double bond can contribute to stabilizing the chain-growth radical in a manner similar to general conjugated monomers.

The vacant p-orbital of boron is known to engage in intermolecular interactions with carbon radical species.^18^ To discuss the effects of the protecting group from the viewpoint of steric hindrance, we built space-filling models of the vinylboron compounds and investigated the sum of the Wiberg bond indices (WBIs)^19^ of chemical bonds around boron (Fig. 2d). The boron center of VBpin is obviously protected by the methyl groups of pinacol (highlighted by the dashed line), and its steric effect was more striking than those of VB(OH)_2_ and VBneop. Thus, the unfavorable results in the polymerizations of VB(OH)_2_ and VBneop, *i.e.*, no polymerization and/or precipitation, should most likely be attributed to the insufficient protection of boron in these monomers. On the other hand, the B–N-containing monomers showed radical polymerization ability, even though the boron atom seems sterically unprotected. Their different behavior of the B–O counterparts is related to the nature of the heteroatoms attached to boron. The B–N-type monomers show larger WBIs than the B–O monomers, indicating a higher electron-donation effect from the lone pair of the neighboring nitrogen to boron. The lone-pair-donation effect likely contributes to the suppression of the attack of the radical on boron even when little steric protection is available. This notion is supported by reports in which nitrogen has been used to suppress the decomposition of relatively unstable boron-containing compounds.^20^ Overall, the key factors for the design of boron monomers for radical polymerizations are: (i) the use of an sp^2^-type boron for the stabilization of the growing radical, and (ii) kinetic protection of boron from radical species through steric shielding, *e.g.*, by the pinacol moiety in VBpin, or thermodynamic protection by electron donation from a lone pair of electrons on nitrogen, *e.g.*, by diaminonaphthalene in VBdan.

**Fig. 3:**
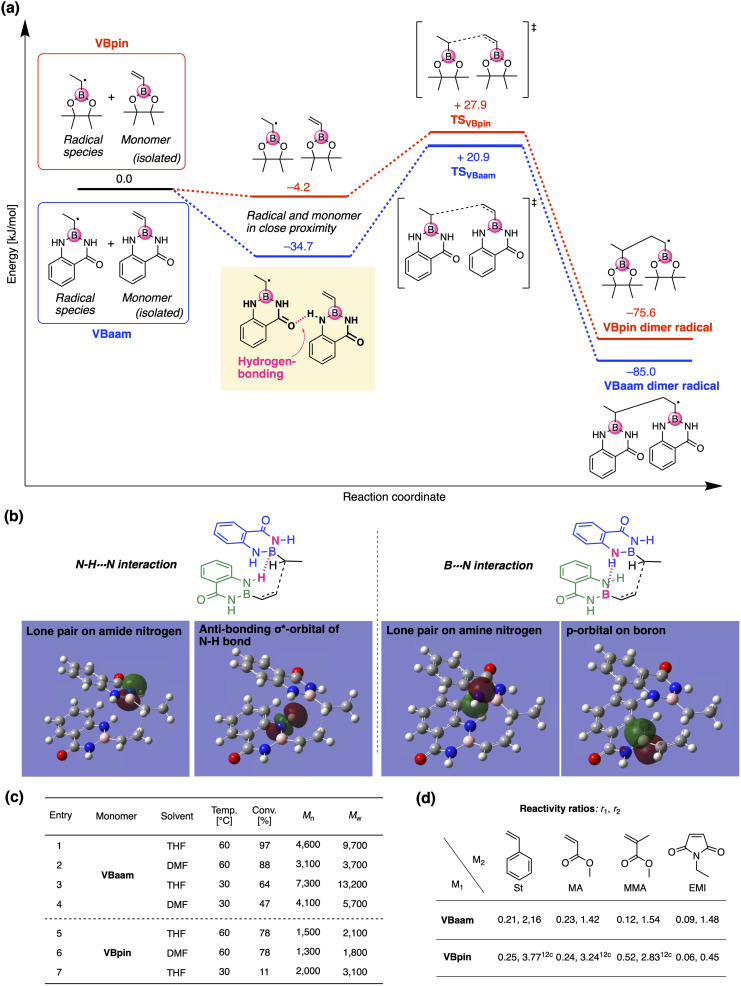
Analysis of the (co)polymerization behavior of VBaam: (a) comparison of the energy diagram of the chain-growth reactions of VBaam and VBpin, calculated at the (U)CAM-B3LYP/6-31G(d) level. (b) Interactions between two aam groups observed in the NBO calculations. (c) Effect of the conditions on the polymerization of VBaam and VBpin. (d) Monomer reactivity ratios of the boron monomers (M_1_) in copolymerizations with common vinyl monomers (M_2_) [*r*_1_ (left), *r*_2_ (right)].

Next, we used DFT calculations to further investigate the cause of the higher polymerization activity of VBaam compared to that of VBpin. For that purpose, we calculated the energy diagram of the chain-growth reaction (Fig. 3a). The approach of the VBaam radical to the monomer resulted in a large energy gain (−34.7 kJ mol^−1^), which was attributed to hydrogen bonding between the protecting groups (C=O⋯H–N). On the other hand, such a significant energy gain was not observed in the case of VBpin (−4.2 kJ mol^−1^). Moreover, the transition state of VBaam was more stable than that of VBpin (VBaam: +20.9 kJ mol^−1^; VBpin: +27.9 kJ mol^−1^). The higher activity of VBaam is partly related to these differences. The obtained transition-state structure was further analyzed through NBO calculations (Fig. 3b). The calculations indicated that the intermolecular interactions between two aam groups based on N–H⋯N hydrogen bonding (2.6 kJ mol^−1^) and B⋯N interactions (0.7 kJ mol^−1^) contribute to a stabilization of the transition state. This interaction between protecting groups likely causes the faster polymerization of VBaam. We then examined the effects of solvent and temperature on the polymerization behavior (Fig. 3c). When the solvent was changed from THF to DMF, which is able to interfere with the hydrogen bonding of the amides, the conversion after 24 h and the molecular weight of the obtained polymer decreased (entry 1 *vs.* 2). When the polymerization was performed at 30 °C with V-70 as the initiator, the molecular weight increased, while the polymerization slowed (entry 3 and 4). These results likely support the contribution of the protecting-group interaction to the improvement of the polymerization activity. On the other hand, in the case of VBpin, the effects of solvent and temperature were smaller, and the conversions and molecular weights were much lower under all applied conditions (entry 5–7).

We then studied the copolymerization behavior of VBaam with common monomers such as styrene (St), methyl methacrylate (MMA), methyl acrylate (MA), and *N*-ethyl maleimide (EMI), and determined the monomer reactivity ratios (*r*_1_ and *r*_2_ for VBaam as M_1_) (Fig. 3d, Tables S2–S6, and Fig. S11–S15†). The copolymerization with St as M_2_ furnished *r*_1_ = 0.21 and *r*_2_ = 2.16; these values indicate that the quantity of VBaam introduced in the 1 : 1 copolymerization is small. Comparing these values with those for the copolymerization of VBpin with St (*r*_1_ = 0.25; *r*_2_ = 3.77)^12*c*^ revealed that particularly *r*_2_ is decreased, indicating that the copolymerization ability of VBaam with styrene was improved. A similar trend was observed for the reactivity ratios of the copolymerizations with MA. In the copolymerizations with MMA, the reactivity ratios of both components were greatly decreased for VBaam (*r*_1_ = 0.12; *r*_2_ = 1.54) relative to those of VBpin (*r*_1_ = 0.52; *r*_2_ = 2.83), and thus, its copolymerization ability was significantly improved. In the copolymerization of VBaam with EMI, *r*_1_ was almost zero and *r*_2_ became higher than 1; this stands in contrast to the EMI/VBpin pair, which afforded low values for both *r*_2_ and *r*_1_ meaning alternation-rich copolymerization. This is probably – at least in part – due to the lower electron density of the double bond of VBaam compared to that of VBpin, as supported by ^1^H NMR spectroscopy (Fig. S16†). Thus, we discovered that the protecting group on boron affects the copolymerization behavior. The time–conversion plots for the copolymerization of VBaam with common monomers (St, MMA, MA, EMI) were also evaluated, together with the molecular weight of the resulting copolymers; the monomer-consumption values were consistent with the trend in the reactivity ratios, and the molecular weight of the copolymers reached 3.08 × 10^4^ (Fig. S17–S20†).

**Fig. 4:**
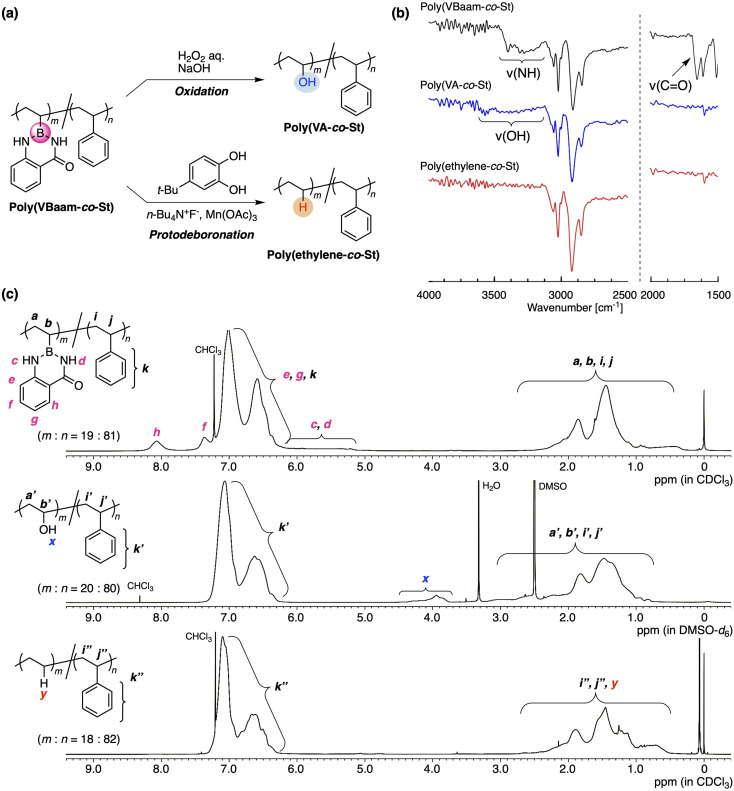
Transformability of the VBaam unit in the copolymer: (a) C–B bond-cleaving oxidation and protonation. (b) FT-IR and (c) ^1^H NMR spectra of poly(VBaam-*co*-St), poly(vinyl alcohol-*co*-St), and poly(ethylene-*co*-St).

Our interest was then directed to the transformability of the VBaam-containing copolymers in polymer reactions (Fig. 4a). We chose St as the comonomer to investigate post-polymerization reactions, mostly on account of its chemical robustness. Poly(VBaam-*co*-St) was prepared *via* the radical copolymerization of VBaam and St with a 1 : 2 monomer molar ratio (Fig. S21;†*M*_n_ = 1.73 × 10^4^; *M*_w_ = 3.10 × 10^4^; unit ratio (*m* : *n*) = 19 : 81, where *m* is VBaam and *n* is St). We first performed the transformation into vinyl alcohols *via* oxidation, which had previously been achieved in Bpin and azaborin side chains. In the IR spectrum, the peak derived from the stretching vibration of the carbonyl group of aam at ∼1700 cm^−1^ completely disappeared, and a broad peak at ∼3300 cm^−1^ that corresponds to a hydroxy group emerged (black and blue lines in Fig. 4b and S22†). This transformation was supported by the ^13^C NMR spectrum, in which a peak for the methine carbon neighboring the hydroxy group was clearly observed (Fig. S23b†). The transformation was further monitored by ^1^H NMR spectroscopy (Fig. 4c). The aromatic peaks of anthranilamide (h, f) disappeared, while a broad peak for the hydroxy group (x) appeared. On the other hand, the peaks for the phenyl protons remained virtually unchanged, and thus, the transformation proceeded without damaging the styrene units. The unit ratio (*m* : *n*) determined by peak integration was 20 : 80, which is almost consistent with that before the transformation. Thus, VBaam is suitable as a precursor monomer for vinyl-alcohol units, and the synthesis of the styrene/vinyl-alcohol copolymer was achieved.

The group of Aggarwal has reported the protonation of the C–B bond in small organic molecules using tetrabutylammonium fluoride (TBAF), Mn(OAc)_3_, and 4-*tert*-butylpyrocatechol (TBC).^21^ This organic reaction encouraged us to explore the transformation of the VBaam into ethylene units in the polymer reaction of VBaam-containing copolymers (Fig. S24†). The reaction of poly(VBaam-*co*-St) with TBAF/Mn(OAc)_3_/TBC under conditions similar to those of Aggarwal gave a product whose IR spectrum contained no peaks from the carbonyl or amine moieties of aam (1650 and 3200 cm^−1^). There is a potential risk that oxidation could occur, which would lead to the introduction of some hydroxy pendants; however, the occurrence of this potential side-reaction could be discarded given that a characteristic peak at ∼3300 cm^−1^ was not observed (red line in Fig. 4b and S22†). In the ^13^C NMR spectrum, multiple peaks were observed between 25 and 38 ppm, and these were attributed to the ethylene moieties in the ethylene–styrene sequence based on a literature report of copolymers synthesized *via* coordination polymerization (Fig. S25†).^22^ The transformation of the VBaam unit into an ethylene unit was further supported by ^1^H NMR spectroscopy. The peaks derived from the aam protons (c, d, h, f) disappeared, while a broad peak, most likely derived from the replacement hydrogen atom (y), appeared at 0.8 ppm. Importantly, the peak integration ratio was consistent with the assumption of a quantitative transformation into ethylene units; the experimentally calculated unit ratio (*m* : *n*) was 18 : 82, which is almost identical to that before the reaction (19 : 81). The thermal properties of the polymers were clearly affected by these transformations (Fig. S26†); the glass-transition temperature (*T*_g_) of poly(VBaam-*co*-St) (*T*_g_ = 106.1 °C) was higher than that of the styrene homopolymer (90.9 °C) due to the interaction between the VBaam units, and the transformation of the VBaam units lowered the *T*_g_ [poly(VA-*co*-St): 86.5 °C; poly(ethylene-*co*-St): 68.5 °C]. It should also be noted here that the copolymer of VBpin with styrene [poly(VBpin-*co*-St) (*m* : *n* = 20 : 80); Fig. S27†] was not quantitatively transformed into the corresponding ethylene copolymer (73% transformation; Fig. S28†) under otherwise identical conditions. The almost quantitative transformation of VBaam is probably due to its lower steric demand than that of VBpin, as shown in the space-filling model in Fig. 2d, which can enhance the reactivity of the boron center toward the reactant (fluoride anion or manganese salt). Higher reactivity of the aam-protected aryl boron center compared to that of the pinacol-protected boron center has already been reported for the simple hydrolysis of low-molecular-weight compounds.^5*a*^

**Fig. 5:**
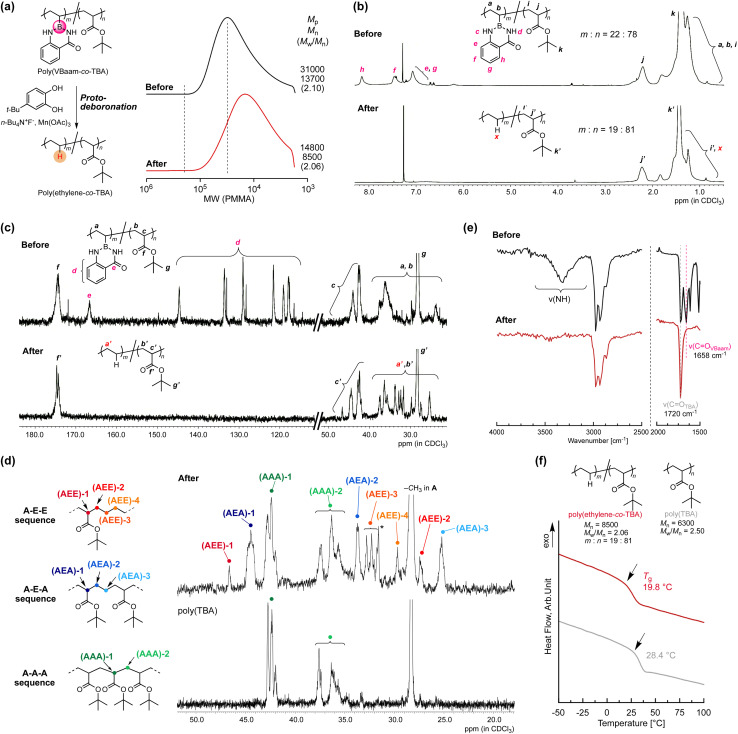
Synthesis of poly(ethylene-*co*-TBA) by C–B bond transformation of poly(VBaam-*co*-TBA); change in the (a) SEC trace and the (b) ^1^H NMR and (c) ^13^C NMR spectra. (d) A comparison of the aliphatic region in the ^13^C NMR spectra of poly(ethylene-*co*-TBA) and the TBA homopolymer (the asterisk denotes the peak from the *t*-butyl group of a very small amount of TBC). (e) Change in the IR spectra due to protodeboronation. (f) DSC curves of poly(ethylene-*co*-TBA) and TBA homopolymer.

Copolymers of ethylene with polar monomers such as acrylates have attracted attention for materials applications,^23^ and their synthesis has been attempted *via* two polymerization systems: radical polymerization and metal-coordination polymerization. The latter approach tends to afford copolymers with a low ratio of polar monomer units, albeit that the recent development of elaborately designed ligands for late transition metals has allowed higher incorporation ratios of polar monomer units.^24^ On the other hand, in radical copolymerizations, increasing the ratio of ethylene units is rather challenging.^25^ In 2017, Chapman *et al.* described the transformation of acrylic acid unit for synthesis of olefin-acrylate copolymers.^26^ Quite recently, Theato and Sumerlin have independently reported radical copolymerizations of *N*-(acryloyloxy)phthalimide with acrylate, followed by a decarboxylation in the presence of a hydrogen-atom donor to access ethylene–acrylate copolymers that are unusually rich in ethylene units.^27,28^ The copolymerization ability of VBaam and its transformation into ethylene *via* protodeboronation after polymerization could also be expected to improve the restricted range of ethylene units in acrylate–ethylene copolymers. We thus examined the radical copolymerization of VBaam with *tert*-butyl acrylate (TBA), followed by the transformation of VBaam into ethylene. Herein, cyanomethyl dodecyl trithiocarbonate (CMDT) was used as the chain-transfer agent for the reversible addition–fragmentation chain transfer (RAFT) copolymerization in order to ensure a uniform incorporation ratio of ethylene in the chain (Fig. S29†). The VBaam : TBA injection monomer molar ratio in the copolymerization was 1 : 2, and the conversion of the monomers after 8 h was 60% and 81%, respectively (Fig. S29a†). The molecular-weight distributions of the obtained copolymers were not particularly narrow, but the SEC curves shifted to higher molecular weight with increasing conversion of both monomers, indicating that the copolymerization was moderately controlled (Fig. S29b†). The resultant copolymer was subjected to the aforementioned protodeboronation treatment in order to transform the VBaam units into ethylene units (Fig. 5a and S30†). The SEC curve of the product largely shifted to lower molecular weight [*M*_p_ (peak top molecular weight) shift: 3.10 × 10^4^ → 1.48 × 10^4^], supporting a decrease in molecular weight due to the replacement of the Baam side chain with a proton. One possible side reaction is hydrolysis to give a boronic-acid side chain, which would broaden the SEC curve toward higher molecular weight due to the often-observed intermolecular trimerization of boronic acids. However, the SEC curve after transformation was not broadened (*M*_w_/*M*_n_: 2.10 → 2.06), *i.e.*, this undesired reaction most likely did not take place. The transformation was further characterized by ^1^H and ^13^C NMR spectroscopy. In the ^1^H NMR spectra, the peaks for the aam aromatic protons (e–h) disappeared, whereas that for the *tert*-butyl group (k and k′) remained (Fig. 5b). Unfortunately, the peak from the replacement proton (x) was not clearly detected due to peak overlap. However, the integration ratio of the peak for the backbone methine proton of the TBA unit (2.2 ppm) relative to those of the other peaks (0.5–2.5 ppm) indicated a 19 : 81 molar ratio between ethylene and TBA, which is close to the 22 : 78 ratio between VBaam and TBA before the transformation, thus supporting an almost quantitative transformation. The ^13^C NMR spectra were also consistent with the transformation of VBaam to ethylene (Fig. 5c); the peaks for the aromatic carbons (d) and the carbonyl carbon (e) of the Baam pendant disappeared, while the peaks characteristic for the TBA unit (g′ for *tert*-butyl and f′ for carbonyl) were retained. We further characterized the peaks from the aliphatic backbone carbons at 20–50 ppm in more detail with reference to a report on the microstructure of a acrylate–ethylene copolymer prepared *via* metal-coordination polymerization^29^ to confirm the transformation to ethylene (Fig. 5d). For comparison, the TBA homopolymer [poly(TBA)] was also measured; the peaks overlapping with those in the spectrum of poly(TBA) can be assigned to a sequence of successive TBA units (A–A–A sequence). We observed peaks from the backbone methine of the TBA unit [(AAA)-1] and those from the backbone methylene [(AAA)-2]. Two peaks were present at lower magnetic field than that of (AAA)-1, which are likely derived from the backbone methine of a TBA unit neighboring an ethylene unit; the peak at 44.5 ppm was attributed to the sequence AEA (acrylate adjacent to isolated ethylene), and another at lower magnetic field (46.7 ppm) to AEE (an isolated acrylate with successive ethylene units). Moreover, some peaks from the methylene carbons in the backbone in ethylene were observed from 25 to 35 ppm. Based on the literature, these peaks were assigned to the ethylene backbone carbons in an AEA sequence [(AEA)-2 and -3] and an AEE one [(AEE)-2, -3, and -4]. The relatively small peaks corresponding to AEE as compared to those of AEA indicate that successive VBaam units are rare, which is consistent with the expectations from the monomer-reactivity ratios of VBaam and acrylate. The selective conversion of aam was also supported by FT-IR spectroscopy (Fig. 5e and S31†). The stretching vibration peaks of C=O and N–H in aam at 1658 cm^−1^ and 3300 cm^−1^ disappeared, whereas the C=O peak of the *tert*-butyl ester at 1720 cm^−1^ clearly remained. The possibility of another side reactions, such as the hydrolysis of the TBA unit, could be discarded as no carboxylic-acid-derived peak was observed at ∼3000 cm^−1^. Based on these structural characterizations, we concluded that poly(VBaam-*co*-TBA) was successfully converted into poly(ethylene-*co*-TBA) *via* protodeboronation. The *T*_g_ of the thus-obtained poly(ethylene-*co*-TBA) (19.8 °C) was markedly lower than that of poly(TBA) (28.4 °C) (Fig. 5f). No melting point was observed due to the low ratio of successive ethylene sequences.

## Conclusions

In this study, we examined the use of various protecting groups on vinylboronic-acid derivatives in order to increase their activity in radical (co)polymerizations as well as to provide access to more versatile transformations in post-polymerization modifications. Our results suggest that among the protecting groups tested, anthranilamide (aam) is the most efficient for both objectives. The crucial points for monomer design that have emerged in this study are: (a) use of an sp^2^-type boron for moderate stabilization of the growing radical; (b) suppression of the attack of radical species on the p-orbital of boron *via* electron donation by a neighboring nitrogen; (c) interactions between two aam groups. The superior transformability of this protecting group was demonstrated for copolymers of aam-protected vinylboronic acid (VBaam) with commodity monomers such as styrene and acrylate. The protected boron pendants in the copolymers were quantitatively converted to hydroxy groups and protons *via* oxidation and protodeboronation, respectively, leading to valuable copolymers such as poly(vinyl alcohol-*co*-styrene), poly(ethylene-*co*-styrene), and poly(ethylene-*co*-acrylate). The tunable polymerization ability and versatile transformability of this bespoke boron monomer should be helpful in pushing the boundaries of conventional polymer synthesis.

## Data availability

All experimental data is available in the ESI.†

## Author contributions

The study was conceptualized and supervised by T. N. and M. O. Most of the experiments were conducted by H. S., and the preliminary study was performed by H. M. DFT-based investigation was carried out by T. N. The manuscript was written by T. N. and M. O. The final version of the manuscript was approved by all authors.

## Conflicts of interest

The authors declare no competing financial interest.

## Supplementary Material

SC-013-D2SC05094C-s001
