# Supplementary material for: Anthranilamide-protected vinylboronic acid: rational monomer design for improved polymerization/transformation ability providing access to conventionally inaccessible copolymers
Source: Chem Sci. 2022 Oct 18;13(43):12703–12. doi: 10.1039/d2sc05094c (PMC9645380; doi:10.1039/d2sc05094c)
Supplement: SC-013-D2SC05094C-s001 [file SC-013-D2SC05094C-s001.pdf]

Electronic Supplementary Material (ESI) for Chemical Science.
This journal is © The Royal Society of Chemistry 2022

Supplementary Information for

Anthranilamide-protected vinylboronic acid: rational monomer design for improved polymerization/transformation ability providing access to conventionally inaccessible copolymers

Hiroshi Suzuki, Tsuyoshi Nishikawa,* Hiroshi Makino, and Makoto Ouchi*

Department of Polymer Chemistry, Graduate School of Engineering, Kyoto University, Kyoto 615-8510, Japan

*To whom correspondence should be addressed.

E-mail: nishikawa.tsuyoshi.8n@kyoto-u.ac.jp
ouchi.makoto.2v@kyoto-u.ac.jp

Contents

1 General Information 2

2 Experimental Procedures 5

3 NMR Spectra of the Synthesized Monomers 11

4 Results of the Free Radical Polymerization of Vinyl-Boronic-Acid Derivatives . 13

5 Determination of the Monomer Reactivity Ratios 17

6 Results of Free Radical Copolymerization of VBaam 23

7 Side-chain Replacement of Poly(VBaam-*co*-St) 25

8 DSC Measurements of St-containing Polymers 30

9 Reactivity of Poly(VBpin-*co*-St) in the Protodeboronation 31

10 RAFT Copolymerization of VBaam with TBA and Protodeboronation 32

11 Computational Study for the Radical Polymerization Behavior 35

12 References 79

1 General Information

Materials

For monomer synthesis: 1,8-Diaminonaphthalene (Wako, >95%), trimethyl borate ($B(OMe)_3$: TCI, >98%), vinylmagnesium bromide (TCI or Aldrich, ca. 1 mol/L in tetrahydrofuran), anthranilamide (Aldrich, >98%), *o*-phenylenediamine (Wako, >95%), acetic anhydride (Ac_2O: Wako, >97%), imidazole (TCI, >98%), iron trichloride ($FeCl_3$: Aldrich, 97%), lithium chloride (LiCl: Kanto Kagaku, >99%), lithium aluminum hydride ($LiAlH_4$: TCI, >95%), neopentyl glycol (TCI, >98%), acetonitrile (CH_3CN: Wako, >99.5%), tetrahydrofuran (THF: Wako, super dehydrated, stabilizer free), ultrapure water (H_2O: Wako), diethyl ether (Et_2O: Wako, >99.5%), *n*-pentane (TCI, >97%), 2-propanol (Nacalai tesque, >99.7%), methanol (Nacalai tesque, >99.8%), sodium hydroxide (NaOH: Wako, >97%), ammonium chloride (NH_4Cl: Wako, >99.5%), sodium chloride (NaCl: Wako, >99.5%), sodium sulfate (Na_2SO_4: Wako, anhydrous, >99%), and magnesium sulfate ($MgSO_4$: Wako, anhydrous, >98%) were used as received. Vinylboronic acid pinacol ester (VBpin: Aldrich, 95%) was purified by bulb-to-bulb distillation prior to use. For purifications using column chromatography: Celite No. 503 (Wako), Wako gel C-200 (Wako), *n*-hexane (Wako, >96%), ethyl acetate (EtOAc: Wako, >99.5%), and dichloromethane (DCM: Wako, >99.5%) were used. 2-Vinyl-2,3-dihydro-1*H*-naphtho[1,8-*de*][1,3,2]diazaborine (VBdan) and 5,5-dimethyl-2-vinyl-1,3,2-dioxaborinane (VBneop) were synthesized according to the literature.[1, 2]

For radical polymerizations: VBpin (Aldrich, 95%), styrene (St: TCI, >99%), methyl methacrylate (MMA: TCI, >99.8%), methyl acrylate (MA: TCI, >99%), and 1,2,3,4-tetrahydronaphthalene (tetralin: TCI, >97%; internal standard for ^{1}H NMR) were purified by distillation prior to use. *tert*-Butyl acrylate (TBA: TCI, >98%) was purified by stirring with inhibitor remover (Aldrich) and filtration prior to use. Vinylboronic acid [$VB(OH)_2$] was prepared *in situ* via the hydrolysis of vinylboronic anhydride pyridine complex (Aldrich, 95%) by adding water (3.0 eq.). 6-Methyl-2-vinyl-1,3,6,2-dioxazaborocane-4,8-dione (VBmida: Aldrich, >97%), potassium vinyltrifluoroborate (VBF_3K: Aldrich, >95%), *N*-ethylmaleimide (EMI: Wako, >98%), 2,2′-azobis(isobutyronitrile) (AIBN: TCI, >98%), 2,2′-azobis(4-methoxy-2,4-dimethylvaleronitrile) (V-70: Wako, >95%), cyanomethyl dodecyl trithiocarbonate (CMDT: Aldrich, >97%; chain-transfer agent for RAFT), toluene (Wako, deoxidized, >99.5%), THF (Wako, deoxidized, stabilizer free, >99.5%), and *N,N*-dimethylformamide (DMF: Wako, deoxidized, >99.5%) were used as received. For purification of the obtained polymers by preparative SEC, chloroform

(CH_3Cl: Wako, >99%), DMF (Wako, >99%), and methanol (Nacalai tesque, >99.8%) were used.

For side-chain replacement in boron-containing copolymers (oxidation and protodeboronation): Sodium hydroxide (NaOH: Wako, >97%), hydrogen peroxide (H_2O_2: TCI, 35% in H_2O), ultrapure water (Wako), tetrabutylammonium fluoride trihydrate ($TBAF \cdot 3H_2O$: Wako, >98%), manganese(III) acetate dihydrate ($Mn(OAc)_3 \cdot 2H_2O$: Aldrich, >97%), 4-*tert*-butylpyrocatecol (TBC: Wako, >98%), ethanol (EtOH: Wako, >99,5%), THF (Wako, with stabilizer, >99.5%), and toluene (Wako, deoxidized, >99.5%) were used as received. For dialysis after the oxidation reaction, MWCO1000 (Spectra/PorVR7, diameter 11.5 mm) was used. For purification by short column chromatography after the protodeboronation reaction, aluminum oxide (Al_2O_3: Merck, 90 active basic, 0.063–0.200 mm), DCM (Wako, >99.5%), and toluene (Nacalai tesque, >99.5%) were used.

Measurements

^{1}H and ^{13}C NMR: ^{1}H NMR (500.16 MHz) and ^{13}C NMR (125.77 MHz) spectra were recorded in $CDCl_3$ or DMSO-d_6 at room temperature (^{13}C NMR spectra of polymers were recorded at 55 °C) on a JEOL JNM-ECA500 spectrometer. The chemical shifts of the ^{1}H and ^{13}C NMR spectra are reported in parts per million (ppm) using tetramethylsilane (TMS) or residual solvent signals as references ($CDCl_3$: δ H = 0.00 ppm (TMS), δ C = 77.16 ppm/DMSO-d_6: δ H = 2.50 ppm, δ C = 39.52 ppm). All coupling constants (J values) are reported in Hertz (Hz). Data for ^{1}H NMR are reported as follows: chemical shift, multiplicity (s = singlet, d = doublet, t = triplet, q = quartet, m = multiplet, brs = broad singlet).

Infrared spectroscopy (IR): IR spectra were recorded on an Agilent Technologies Cary 630 FTIR spectrometer.

Size-exclusion chromatography (SEC): SEC curves, number-average molecular weight (M_n), weight-average molecular weight (M_w), peak-top molecular weight (M_p), and molecular weight distribution (M_w/M_n) of the polymers were measured by SEC in THF at 40 °C (flow rate: 0.35 mL/min) on two polystyrene gel columns (Shodex LF-404) that were connected to an HLC-8320GPC system (TOSOH). The columns were calibrated against twelve standard poly(MMA) samples (PSS Ready Cal Kit: M_p = 800–2200000). The values of polymers that dissolve in DMF were measured by SEC in DMF containing

10 mM LiBr at 40 °C (flow rate: 1 mL/min) on three polystyrene gel columns (Shodex KF-805L) that were connected to a JASCO PU-2080 precision pump, a JASCO RI-2031 refractive index detector, and a JASCO UV-2075 UV/vis detector set at 270 nm. The columns were calibrated against eleven standard poly(MMA) samples (PSS Ready Cal Kit: M_p = 2380–2200000).

Preparative SEC: Unless otherwise noted, vinyl-boronic-acid-based polymers, except for VBaam-containing (co)polymers, were purified via preparative SEC using $CHCl_3$ as the eluent at room temperature (flow rate: 10 mL/min) on JAIGEL-2.5HR (exclusion limit: 20,000) or Shodex KF-5001 (exclusion limit: 1,500) columns. VBaam-containing (co)polymers were purified by preparative SEC using DMF as the eluent at room temperature (flow rate: 15 mL/min) on TOSOH TSKgel α-3000 (exclusion limit: 10,000).

Differential scanning calorimetry (DSC): These measurements were performed on a DSC Q200 calorimeter (TA instrument) equipped with an RCS 90 electric machine under a flow of dry nitrogen at a heating or cooling rate of 10 °C/min. The polymer sample (ca. 3–10 mg) was placed into an aluminum pan. The temperature program was as follows:

For St-containing (co)polymers: first heating from 40 °C to 150 °C → first cooling from 150 °C to 0 °C → second heating from 0 °C to 150 °C → second cooling from 150 °C to 0 °C → third heating from 0 °C to 150 °C.

For TBA-containing (co)polymers: first heating from 40 °C to 140 °C → first cooling from 140 °C to –70 °C → second heating from –70 °C to 140 °C → second cooling from 140 °C to –70 °C → third heating from –70 °C to 140 °C.

The second heating scan was employed to determine the T_g of all polymer samples.

2 Experimental Procedures

Synthesis of 2-vinyl-2,3-dihydrobenzo[*d*][1,3,2]diazaborinin-4(1*H*)-one (VBaam)

Scheme S1. Synthesis of VBaam

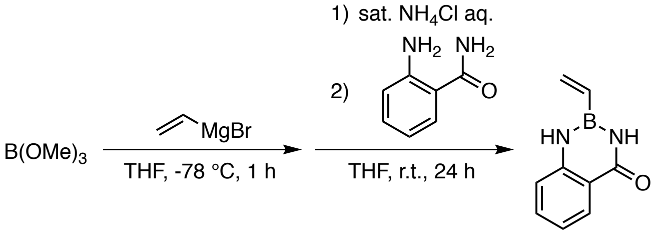

Vinylmagnesium bromide (ca. 1 M in THF, 100 mL, 100 mmol) was added dropwise to a stirred solution of $B(OMe)_3$ (12.5 mL, 112 mmol) in dehydrated THF (94 mL) at –78 °C (cooled by an acetone/dry ice bath) under a dry argon atmosphere. The resulting mixture was stirred for 1 h and then warmed to room temperature, before a saturated NH_4Cl aqueous solution (41 mL) was added. After 10 min, a solution of anthranilamide (11.5 g, 84.3 mmol) in THF (20 mL) was added and stirring was continued for 24 h. The reaction mixture was diluted with EtOAc (200 mL) and passed through a layer of celite. The filtrate was washed with H_2O (2 × 100 mL) and brine (100 mL). The collected organic phase was dried over Na_2SO_4 and filtered, before the filtrate was concentrated under reduced pressure. The thus obtained crude residue was purified by column chromatography on silica gel [eluent: *n*-hexane/EtOAc = 2/1 (v/v)] to obtain the product as a white solid (8.65 g, 50.3 mmol, 60%).

^{1}H NMR (500 MHz, DMSO-d_6): δ (ppm) 9.39 (brs, 1H), 9.07 (brs, 1H), 7.96 (dd, J = 8.0, 1.0 Hz, 1H), 7.52 (dt, J = 7.8, 2.0 Hz, 1H), 7.25 (d, J = 8.0 Hz, 1H), 7.06 (dt, J = 7.5, 1.0 Hz, 1H), 6.35 (dd, J = 20.0, 3.5 Hz, 1H), 6.13 (dd, J = 19.5, 13.5 Hz, 1H), 6.00 (dd, J = 13.5, 3.5 Hz, 1H).

^{13}C NMR (126 MHz, DMSO-d_6): δ (ppm) 166.0, 145.3, 134.1, 133.3, 132.3, 127.9, 120.6, 118.9, 117.9.

IR: 1153, 1281, 1485, 1513, 1616, 1659, 3222, 3318, 3415 cm^{-1}

ESI-MS: Calculated for $[M+H]^+$ ($C_9H_{10}BN_2O$): 173.0881, Found: 173.0879.

Synthesis of 1,3-diethyl-2-vinyl-2,3-dihydro-1*H*-benzo[*d*][1,3,2]diazaborole (VBdepam)

Scheme S2. Synthesis of VBdepam

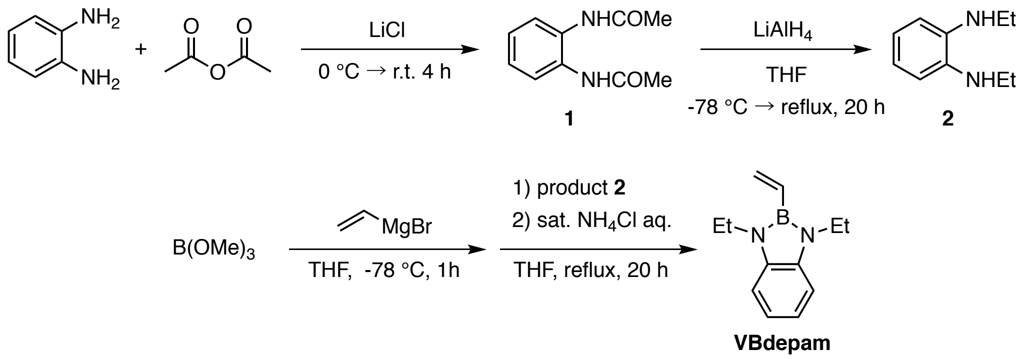

Synthesis of *N,N′*-(1,2-phenylene)diacetamide (1)

Following a literature procedure,[3] Ac_2O (13.0 mL, 132 mmol) and LiCl (0.25 g, 6.01 mmol) were added to *o*-phenylenediamine (6.48 g, 60.0 mmol) at 0 °C (cooled by a water/ice bath), before the mixture was stirred at room temperature for 4 h. The resulting precipitate was isolated by filtration, washed with *n*-pentane, and dried under reduced pressure. The thus obtained white solid (8.61 g, 44.8 mmol, 75%) was used for subsequent synthesis of *N,N′*-diethyl-*o*-phenylenediamine without further purification.

^{1}H NMR (500 MHz, DMSO-d_6): δ (ppm) 9.32 (brs, 2H), 7.54 (dd, J = 6.0, 3.5 Hz, 2H), 7.11 (dd, J = 6.0, 3.0 Hz, 2H), 2.07 (s, 6H).

Synthesis of *N,N′*-diethyl-*o*-phenylenediamine (2)

Following a literature procedure,[3] **1** (5.77g, 30.0 mmol) was slowly added to a THF solution (102 mL) of $LiAlH_4$ (2.60 g, 68.4 mmol) at –78 °C under a dry argon atmosphere. The mixture was refluxed for 20 h and then cooled at 0 °C, before EtOAc (10 mL), 2-propanol (10 mL), methanol (10 mL), and 1 M NaOH aqueous solution (90 mL) were added to the reaction flask in order to deactivate any residual reductant. The reaction mixture was then diluted with DCM (150 mL) and passed through a layer of celite. The filtrate was washed with brine (3 × 50 mL). The collected organic phase was dried over $MgSO_4$ and filtered, before the filtrate was concentrated under reduced pressure. The thus obtained dark-red oil was purified by column chromatography on silica [eluent: *n*-hexane/EtOAc = 8/1 (v/v)] to obtain the product as a light-orange oil (3.37 g, 20.5 mmol, 68%).

^{1}H NMR (500 MHz, $CDCl_3$): δ (ppm) 6.79 (dd, J = 5.8, 3.5 Hz, 2H), 6.67 (dd, J =

5.5, 3.5 Hz, 2H), 3.14 (brq, J = 7.5 Hz, 6H), 1.30 (t, J = 7.3 Hz, 6H).

Synthesis of VBdepam

Vinylmagnesium bromide (ca. 1 M in THF, 26.7 mL, 26.7 mmol) was added dropwise to a stirred solution of $B(OMe)_3$ (3.5 mL, 30.8 mmol) in dehydrated THF (17 mL) at –78 °C under a dry argon atmosphere. The resulting mixture was stirred for 1 h and then warmed to room temperature. A THF solution (3.0 mL) of **2** (2.48 g, 15.1 mmol) was added, and the mixture was refluxed for 20 h. Subsequently, the solution was cooled to room temperature, before a saturated aqueous solution of NH_4Cl (7.3 mL) was added. The reaction mixture was diluted with EtOAc (120 mL) and passed through a layer of celite. The filtrate was washed with brine (3 × 100 mL). The collected organic phase was dried over Na_2SO_4 and filtered, before the filtrate was concentrated under reduced pressure. The crude residue was purified by column chromatography on silica gel [eluent: *n*-hexane/EtOAc = 100/1 (v/v)] to afford the target product as a light-orange oil (2.12 g, 10.6 mmol, 70%).

^{1}H NMR (500 MHz, $CDCl_3$): δ (ppm) 7.06–7.03 (m, 2H), 7.01–6.97 (m, 2H), 6.39 (dd, J = 20.0, 15.0 Hz, 1H), 6.02 (dd, J = 14.5, 3.0 Hz, 1H), 5.92 (dd, J = 20.5, 3.5 Hz, 1H), 3.83 (q, J = 7.0 Hz, 4H), 1.31 (t, J = 7.3 Hz, 6H).

^{13}C NMR (126 MHz, $CDCl_3$): δ (ppm) 137.2, 131.6, 118.6, 108.5, 37.6, 16.3. The boron-bearing carbon atom was not observed.

IR: 1260, 1305, 1373, 1437, 1483, 1601, 1892, 2881, 2969, 3053 cm^{-1}

ESI-MS: Calculated for $[M\cdot]^+$ ($C_{12}H_{17}BN_2$): 200.1485, Found:200.1482.

Free radical polymerization of vinyl-boronic-acid derivatives

The polymerization was performed in various solvents (THF, DMF, or toluene) using AIBN (at 60 °C) or V-70 (at 30 °C) as an initiator under an inert gas atmosphere. The typical procedure for the polymerization of VBpin in THF at 60 °C was as follows: VBpin (170 μL, 154 mg, 1.0 mmol), tetralin (13.6 μL, 13.2 mg, 100 μmol, internal standard), and THF (153 μL) were placed in a Schlenk tube with a PTFE stopcock (J. Young) under a dry argon atmosphere. A THF solution of AIBN (164 μL of 10 mg/mL, 10 μmol) was then added at room temperature. The reaction mixture was stirred at 60 °C in an oil bath. The conversion of VBpin was monitored by ^{1}H NMR spectroscopy: Conv. = 78% after 24 h. All volatiles were removed under reduced pressure, before an SEC analysis was carried out to determine the M_n, M_w, and M_w/M_n of the resultant polymer (M_n = 1.5 × 10^3; M_w = 2.1 × 10^3; M_w/M_n = 1.43). The polymerization of other vinyl-boronic-acid

derivatives was performed in a similar way.

Free radical copolymerization of VBaam with common vinyl monomers

The copolymerization was carried out in DMF at 60 °C with AIBN as the initiator under an inert gas atmosphere with the following injection ratio: $[\text{VBaam}]_0$ = $[\text{comonomer}]_0$ = 1 M, $[\text{AIBN}]_0$ = 20 mM. The typical procedure for the copolymerization with styrene as a comonomer was as follows: VBaam (172 mg, 1.0 mmol), styrene (St, 114 μL, 104 mg, 1.0 mmol), tetralin (27.3 μL, 26.4 mg, 100 μmol, internal standard), and DMF (530 μL) were placed in a Schlenk tube with a PTFE stopcock (J. Young) under a dry argon atmosphere. A DMF solution of AIBN (328 μL of 10 mg/mL, 20 μmol) was then added at room temperature. The reaction mixture was stirred at 60 °C in an oil bath. The conversion of both monomers was monitored by ^{1}H NMR spectroscopy: Conv. (VBaam) = 28%, Conv. (St) = 78% after 72 h. Then, all volatiles were removed under reduced pressure, before an SEC analysis was carried out to determine the M_n, M_w, and M_w/M_n of the resultant polymer (M_n = 10.9 × 10^3; M_w = 19.2 × 10^3; M_w/M_n = 1.77). Copolymerizations with other vinyl monomers were performed in a similar way. The obtained copolymers were purified by preparative SEC, and the unit ratios of VBaam (F_VBaam) in the copolymers were calculated by ^{1}H NMR analysis.

Oxidation of poly(VBaam-*co*-St) to synthesize poly(VA-*co*-St)

Poly(VBaam-*co*-St) was synthesized via free radical copolymerization in THF at 60 °C for 72 h with the following injection ratio: $[\text{VBaam}]_0$ = 1 M, $[\text{St}]_0$ = 2 M, $[\text{AIBN}]_0$ = 20 mM. After purification by preparative SEC, the obtained poly(VBaam-*co*-St) (300 mg; F_VBaam = 20 mol%, M_n = 19.1 × 10^3, M_w/M_n = 1.73) was dissolved in THF (85 mL). Then, ethanol (9.0 mL), an aqueous solution of NaOH (6 M, 9.0 mL), and an aqueous solution of H_2O_2 (35 wt%, 18 mL) were added. The reaction solution was stirred at 65 °C under atmospheric conditions. After 24 hours, the volume of the solution was reduced to about 20 mL by evaporation. The products were subjected to dialysis in order to remove any residues of lower molecular weight. Dialysis was performed several times with substitution of the solvent in the following order: water → water/THF = 50/50 (v/v) → water/THF = 30/70 (v/v) → THF. For a detailed structural analysis, the obtained polymer was further purified by preparative SEC using $CHCl_3$ as the eluent (199 mg).

Protodeboronation of poly(VBaam-*co*-St) and poly(VBpin-*co*-St) to synthesize poly(ethylene-*co*-St)

The poly(VBaam-*co*-St) (300 mg; F_VBaam = 19 mol%, M_n = 18.9 × 10^3, M_w/M_n =

1.67), TBAF·3H_2O (186 mg, 588 µmol), Mn(OAc)$_3$·2H_2O (132 mg, 490 µmol), TBC (408 mg, 2.45 mmol), and deoxidized toluene (7.0 mL) were placed in a Schlenk tube with a three-way stopcock under a dry argon atmosphere. The reaction mixture was stirred at 80 °C. After 24 h, the mixture was cooled at room temperature and then diluted with DCM (50 mL). The solution was washed with water (2 × 50 mL) and brine (50 mL). The collected organic phase was dried over Na_2SO_4, filtered, and passed through a basic Al_2O_3 column to remove residual TBAF and Mn salts. After evaporation of the filtrate, the residue was purified by preparative SEC using $CHCl_3$ as the eluent (62 mg).

The protodeboronation reaction was also applied to poly(VBpin-*co*-St) (F_{VBpin} = 20 mol%, M_n = 13.3 × 10^3, M_w/M_n = 1.72) synthesized via free radical polymerization in THF at 60 °C for 72 h with the following injection ratio: $[\mathrm{VBpin}]_0$ = $[\mathrm{St}]_0$ = 1.5 M, $[\mathrm{AIBN}]_0$ = 20 mM.

Synthesis of poly(ethylene-*co*-TBA) by protodeboronation of the VBaam unit

Synthesis of the precursor poly(VBaam-*co*-TBA):

VBaam (4.59 g, 26.7 mmol), TBA (7.78 mL, 6.84 g, 53.3 mmol), CMDT (63.5 mg, 200 µmol), tetralin (273 µL, 264 mg, 2.0 mmol, internal standard), and DMF (9.65 mL) were placed in a round-bottom flask equipped with a three-way stopcock under a dry argon atmosphere. A DMF solution of AIBN (2.30 mL of 10 mg/mL, 140 µmol) was then added at room temperature. The reaction mixture was stirred at 60 °C in an oil bath. The conversion of the monomers and the molecular weight of resulting polymer were monitored by ^{1}H NMR spectroscopy and SEC: Conv.(VBaam) = 60%, Conv.(TBA) = 81%, M_n = 15.5 × 10^3, M_w/M_n = 1.99 after 8 h. The polymerization solution was added dropwise into 1 L of stirred MeOH/water (8/2, v/v) in order to purify the obtained polymer via precipitation. The collected polymer was dissolved in $CHCl_3$ and dried over Na_2SO_4. After filtration, the solvent was removed by evaporation, and poly(VBaam-*co*-TBA) was obtained as a yellow solid (7.47 g).

Protodeboronation of poly(VBaam-*co*-TBA):

Poly(VBaam-*co*-TBA) (402 mg; F_{VBaam} = 22 mol%, M_n = 13.7 × 10^3, M_w/M_n = 2.10), TBAF·3H_2O (238 mg, 754 µmol), TBC (835 mg, 5.02 mmol), and deoxidized toluene (9.0 mL) were placed in a Schlenk tube with a three-way stopcock under a dry argon atmosphere. The solution was stirred at 80 °C for 3 h, and the resultant solution was then transferred to another Schlenk tube containing Mn(OAc)$_3$·2H_2O (628 mg, 628 µmol) under an argon atmosphere. The reaction mixture was again stirred at 80 °C. After 24 hours, the mixture was cooled to room temperature and then diluted with toluene (50 mL). The solution was washed with water (3 × 50 mL), and the collected organic phase was

dried over Na_2SO_4, filtered, and passed through a basic Al_2O_3 column to remove TBAF and Mn salts. After evaporation, the residue was purified twice by preparative SEC twice using $CHCl_3$ as the eluent (205 mg).

Synthesis of styrene homopolymer (PSt) and TBA homopolymer [poly(TBA)] for the DSC measurements

Both homopolymers for the DSC measurements were synthesized by free radical polymerization. The polymerization conditions, M_n, and M_w/M_n of the obtained polymer were as follows:

For PSt (M_n = 15.0 × 10^3, M_w/M_n = 1.69): $[St]_0/[AIBN]_0$ = 2000/20 mM in THF at 60 °C for 72 h.

For poly(TBA) (M_n = 6.3 × 10^3, M_w/M_n = 2.50): $[TBA]_0/[AIBN]_0$ = 2000/10 mM in THF at 60 °C for 6 h.

3 NMR Spectra of the Synthesized Monomers

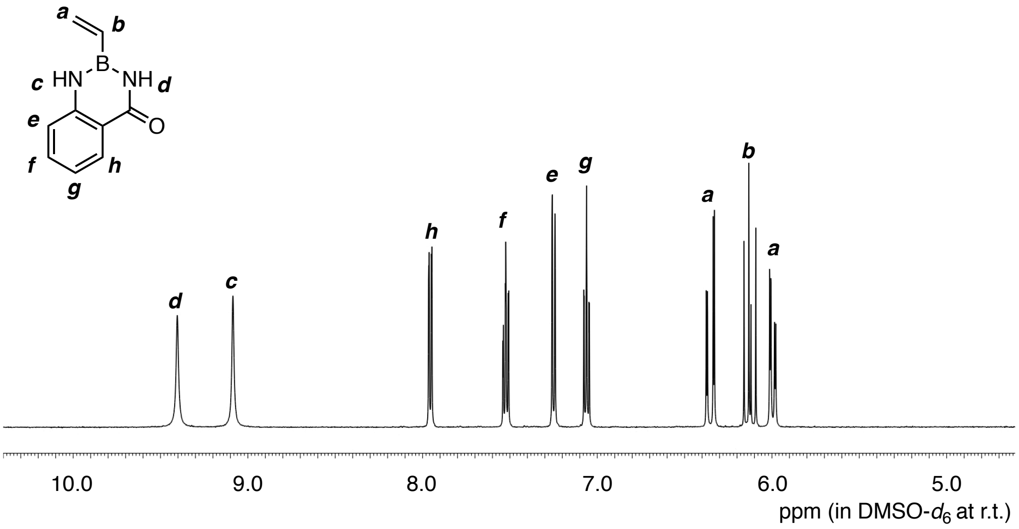

Figure S1. ^{1}H NMR spectrum (in DMSO-d_6 at r.t.) of VBaam.

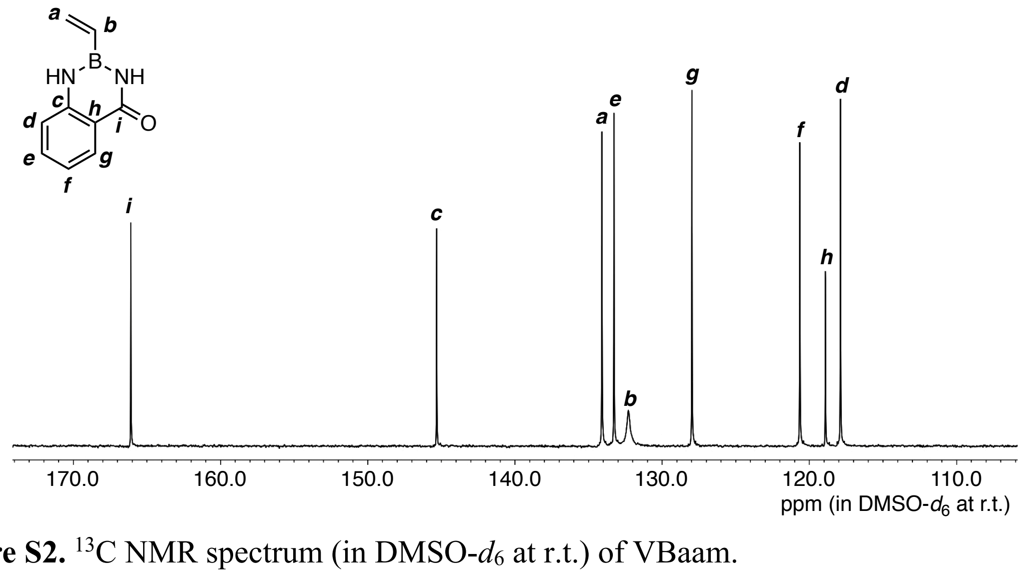

Figure S2. ^{13}C NMR spectrum (in DMSO-d_6 at r.t.) of VBaam.

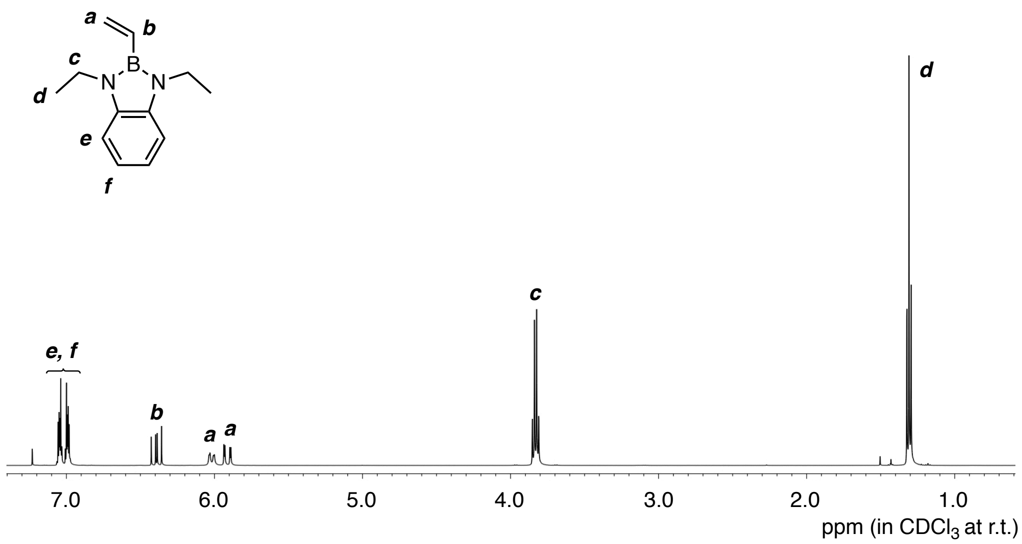

Figure S3. ^{1}H NMR spectrum (in $CDCl_3$ at r.t.) of VBdepam

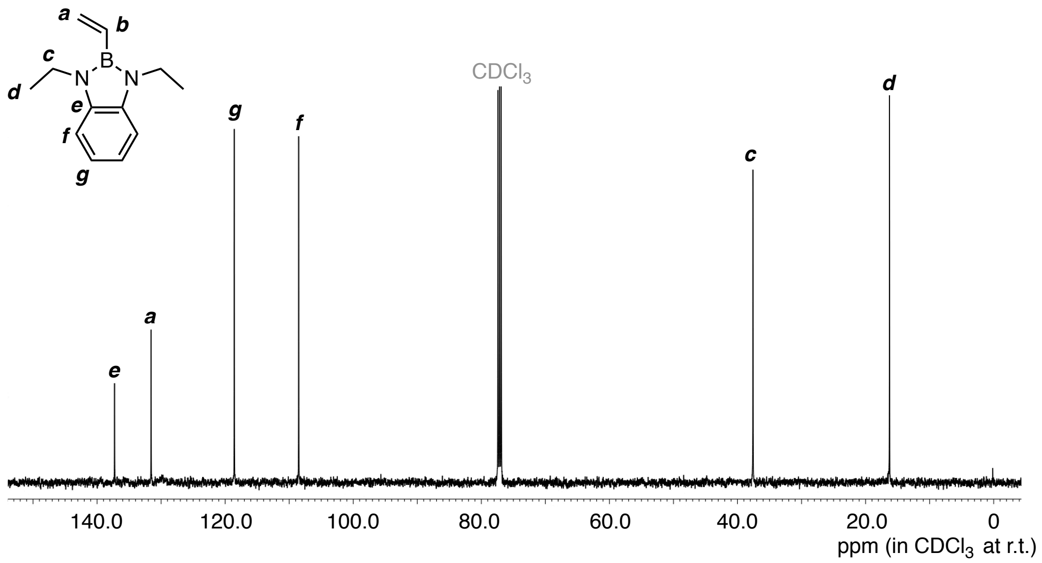

Figure S4. ^{13}C NMR spectrum (in $CDCl_3$ at r.t.) of VBdepam.

4 Results of the Free Radical Polymerization of Vinyl-Boronic-Acid Derivatives

Table S1. Free radical polymerization of several vinylboronic acid derivatives (VBs) [a]

Entry	VBs	Solvent	Temp. [°C]	Time [h]	Conv. [%] [b]	M_n [c]	M_w [c]
1	VBpin	THF	60	24	78	1500	2100
2	VBdan	THF	60	24	60	4000	5800
3	VBaam	THF [e)]	60	24	97	4600	9700
4	VBdepam	THF	60	72	32	4300 [g]	9500 [g]
5 [d]	$VB(OH)_2$	DMF	60	24	3	n.d. [h]	n.d. [h]
6 [d]	VBneop	Toluene	60	24	30 [f)]	n.d. [h]	n.d. [h]
7	VBmida	DMF	60	72	2	n.d. [g, h]	n.d. [g, h]
8	VBF_3K	DMF	60	72	1	n.d. [h]	n.d. [h]

a) Polymerization conditions: $[VBs]_0/[\text{initiator (AIBN or V-70)}]_0$ = 2000/20 mM in the specified solvent at 60 °C or 30 °C. b) Determined by ^{1}H NMR spectroscopy. c) Determined by SEC [eluent: DMF (10 mM LiBr), calib: PMMA]. d) $[VBs]_0/[AIBN]_0$ = 4000/40 mM. e) Polymerization solution suspended in the late stage of polymerization. f) Consumption of vinyl compound just resulted in formation of insoluble material. g) Determined by SEC (eluent: THF, calib: PMMA). h) Not determined.

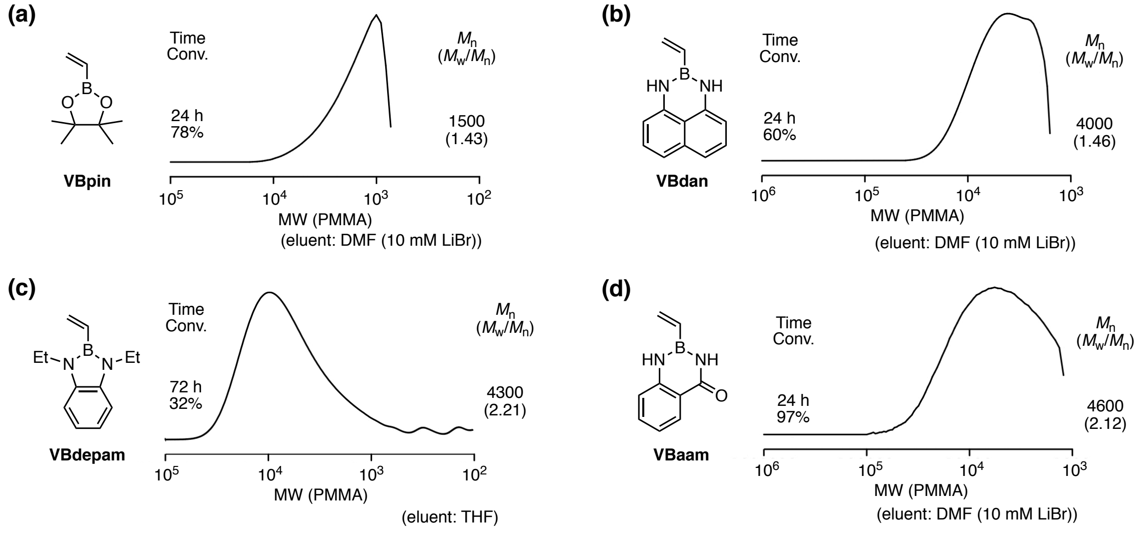

Figure S5. SEC traces of the resulting polymer in the free radical polymerization of VBpin (a), VBdan (b), VBdepam (c), and VBaam (d) (for polymerization conditions, see entries 1–4 in Table S1).

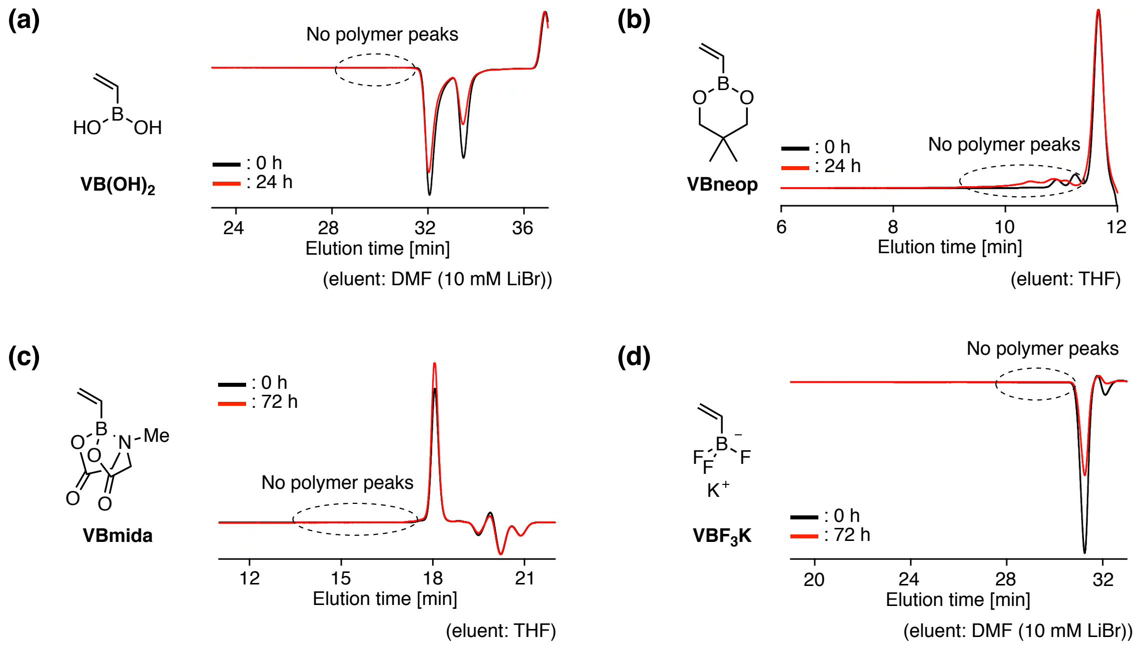

Figure S6. SEC traces in the free radical polymerization of VB(OH)$_2$ (a), VBneop (d), VBmida (c), and VBF$_3$K (d) (for the polymerization conditions, see entries 5–8 in Table S1).

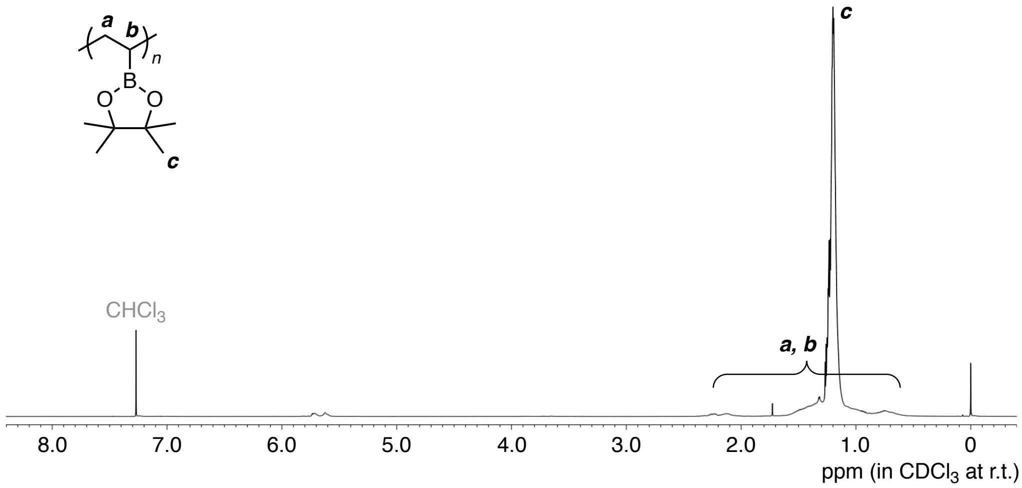

Figure S7. ^{1}H NMR spectrum (in $CDCl_3$ at r.t.) of poly(VBpin) (entry 1 in Table S1).

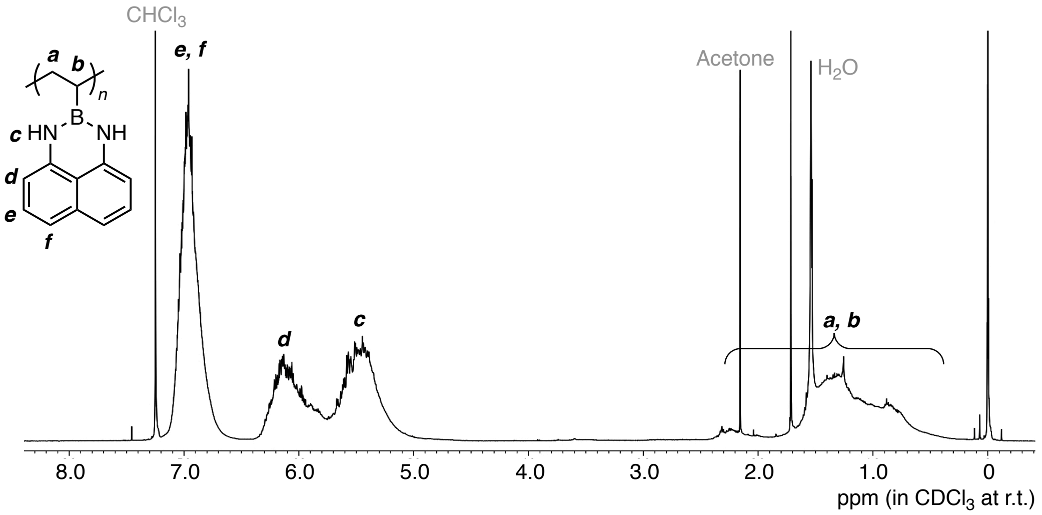

Figure S8. ^{1}H NMR spectrum (in $CDCl_3$ at r.t.) of poly(VBdan) (entry 2 in Table S1).

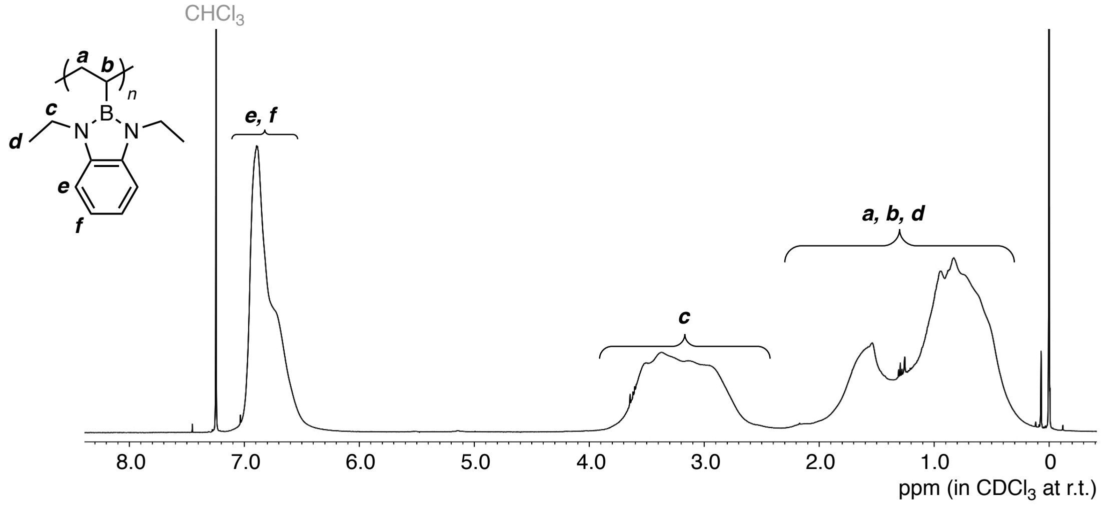

Figure S9. ^{1}H NMR spectrum (in $CDCl_3$ at r.t.) of poly(VBdepam) (entry 4 in Table S1).

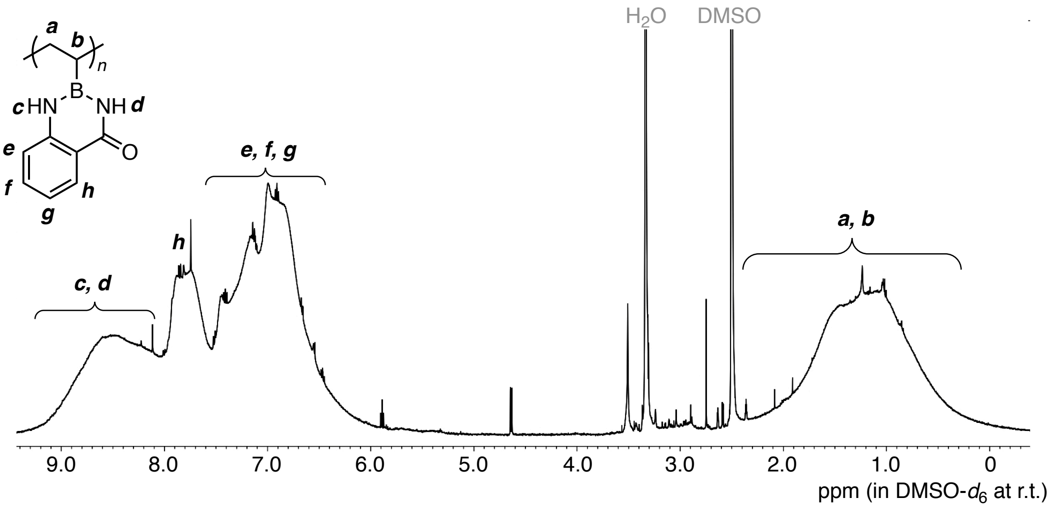

Figure S10. ^{1}H NMR spectrum (in DMSO-d_6 at r.t.) of poly(VBaam) ($M_n = 7.9 \times 10^3$, $M_w/M_n = 1.89$): $[VBaam]_0/[V\text{-}70]_0 = 2000/4$ mM in THF at 30 °C for 48 h.

5 Determination of the Monomer Reactivity Ratios

Monomer reactivity ratios were determined based on the report by Mayo and Lewis.[4] The copolymerization behavior of two monomers (M_1 and M_2) is generally described based on equation (1), where $[M_1]$ is the concentration of M_1, $[M_2]$ is the concentration of M_2, and r_1 and r_2 are the reactivity ratios of M_1 and M_2.

$$\frac{d[M_1]}{d[M_2]} = \frac{[M_1](r_1[M_1] + [M_2])}{[M_2](r_2[M_2] + [M_1])} \qquad (1)$$

When the monomer consumption is sufficiently small, the Mayo–Lewis equation shown above can be approximated to

$$\frac{\Delta[M_1]}{\Delta[M_2]} \fallingdotseq \frac{[M_1]_0(r_1[M_1]_0 + [M_2]_0)}{[M_2]_0(r_2[M_2]_0 + [M_1]_0)} \qquad (2)$$

where $[M_1]_0$ is the concentration of M_1 before the reaction, $[M_2]_0$ is the concentration of M_2 before the reaction, $\Delta[M_1]$ is the consumption of M_1 during the reaction, and $\Delta[M_2]$ is the consumption of M_2 during the reaction.

When F_1 is defined as the composition of M_1 in the consumed monomers during the reaction ($F_1 = \Delta[M_1]/(\Delta[M_1]+\Delta[M_2])$, $F_2 = \Delta[M_2]/(\Delta[M_1]+\Delta[M_2])$), and f_1 as the composition of M_1 in the monomer feed before the reaction ($f_1 = [M_1]_0/([M_1]_0+[M_2]_0)$, $f_2 = [M_2]_0/([M_1]_0+[M_2]_0)$), equation (2) can be expressed as:[5]

$$F_1 = \frac{r_1 {f_1}^2 + f_1(1 - f_1)}{r_1 {f_1}^2 + 2f_1(1 - f_1) + r_2(1 - f_1)^2} \qquad (3)$$

On the basis of equation (3) and the experimental results obtained for f_1 and F_1, nonlinear least-squares fitting of f_1 versus F_1 was performed using the Solver Function in Microsoft Office Excel 2011. The sums of the squares of the deviation were minimized by optimizing r_1 and r_2. The experimental procedure to obtain f_1 and F_1 series in each copolymerization is shown in following section.

The copolymerization of VBaam and vinyl comonomers or of VBpin and EMI was performed using AIBN. The sum of the concentrations of both monomers was 2000 mM,

and the feed ratio of each monomer was varied ([VBaam]$_0$:[comonomer]$_0$ = 90:10, 70:30, 50:50, 30:70, and 10:90). A typical procedure for the copolymerization for the determination of the monomer reactivity ratios was as follows: VBaam (86.0 mg, 0.5 mmol), styrene (57.5 µL, 52.1 mg, 0.5 mmol), tetralin (13.6 µL, 13.2 mg, 100 µmol, internal standard), and DMF (265 µL) were placed in a Schlenk tube with a PTFE stopcock (J. Young) under a dry argon atmosphere. A DMF solution of AIBN (164 µL, 1.64 mg, 10 µmol) was then added at room temperature. (5:5 feed ratio condition). The reaction mixture was placed in an oil bath at 60 °C. After 3 h, the solution was cooled to −25 °C to terminate the polymerization. The composition ratio [$F = DP_{\mathrm{VBaam}}/(DP_{\mathrm{VBaam}} + DP_{\mathrm{comonomer}})$] was determined from the monomer conversion ratio [Conv.(VBaam)/(Conv.(VBaam) + Conv.(comonomer))] by ^{1}H NMR spectroscopy (DMSO-d_6) with tetralin as the internal standard. The actual monomer feed ratio [f = [VBaam]$_0$/([VBaam]$_0$ + [comonomer]$_0$) was also determined from ^{1}H NMR spectroscopy (before heating). The obtained series of f_1 and F_1 values were used to determine r_1 and r_2 on the basis of equation (3) through the non-linear least-squares method (*vide supra*), affording the monomer reactivity ratios. The monomer conversions of each copolymerization are summarized in the following tables.

Table S2. Free radical copolymerization of VBaam (M_1) with St (M_2) for the determination of the monomer reactivity ratios [a)]

f [b)]	Time [h]	Conv.$_{M1}$ [%] [c)]	Conv.$_{M2}$ [%] [c)]	F [d)]
0.08	5	7.4	19.7	0.03
0.29	4	4.9	17.9	0.10
0.50	3	8.0	17.7	0.31
0.70	3	5.7	16.8	0.44
0.90	2	3.3	13.6	0.70

a) $[M_1 + M_2]_0/[\mathrm{AIBN}]_0$ = 2000/20 mM in DMF at 60 °C. b) Actual monomer feed ratio ($f = [M_1]_0/([M_1]_0 + [M_2]_0)$) determined by ^{1}H NMR spectroscopy. c) Determined by ^{1}H NMR spectroscopy. d) Calculated from f, Conv.$_{M1}$, and Conv.$_{M2}$.

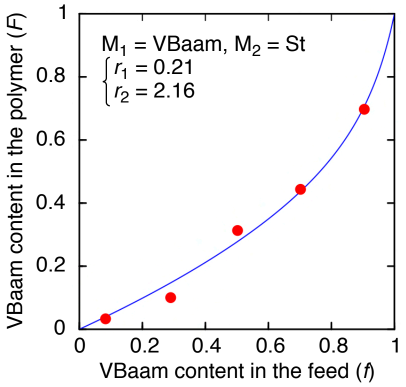

Figure S11. Copolymer composition curve for the copolymerization of VBaam (M_1) and St (M_2) in DMF.

Table S3. Free radical copolymerization of VBaam (M_1) with MMA (M_2) for the determination of the monomer reactivity ratios [a)]

f [b)]	Time [min]	Conv.$_{M1}$ [%] [c)]	Conv.$_{M2}$ [%] [c)]	F [d)]
0.09	20	5.9	13.4	0.04
0.30	20	8.0	14.7	0.19
0.50	30	8.5	16.8	0.33
0.71	30	5.8	20.5	0.41
0.90	30	4.6	22.1	0.66

a) $[M_1 + M_2]_0/[AIBN]_0$ = 2000/20 mM in DMF at 60 °C. b) Actual monomer feed ratio ($f = [M_1]_0/([M_1]_0 + [M_2]_0)$) determined by ^{1}H NMR spectroscopy. c) Determined by ^{1}H NMR spectroscopy. d) Calculated from f, Conv.$_{M1}$, and Conv.$_{M2}$.

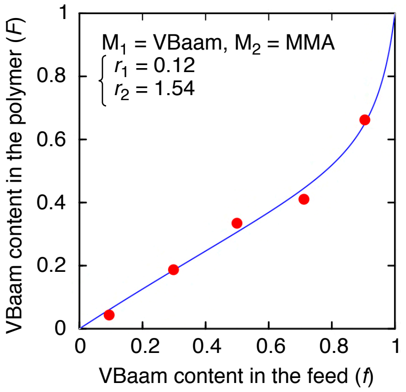

Figure S12. Copolymer composition curve for the copolymerization of VBaam (M_1) and MMA (M_2) in DMF.

Table S4. Free radical copolymerization of VBaam (M_1) with MA (M_2) for the determination of the monomer reactivity ratios [a)]

f [b)]	Time [min]	Conv.$_{M1}$ [%] [c)]	Conv.$_{M2}$ [%] [c)]	F [d)]
0.08	15	5.6	12.4	0.04
0.30	15	4.8	8.5	0.19
0.51	15	8.0	16.3	0.34
0.72	30	7.3	15.5	0.55
0.91	45	6.1	23.7	0.72

a) $[M_1 + M_2]_0/[AIBN]_0$ = 2000/20 mM in DMF at 60 °C. b) Actual monomer feed ratio (f = $[M_1]_0/([M_1]_0 + [M_2]_0)$) determined by ^1H NMR spectroscopy. c) Determined by ^1H NMR spectroscopy. d) Calculated from f, Conv.$_{M1}$, and Conv.$_{M2}$.

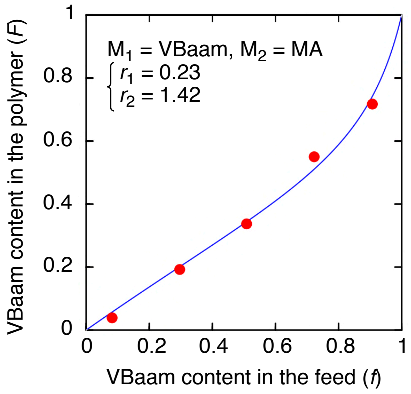

Figure S13. Copolymer composition curve for the copolymerization of VBaam (M_1) and MA (M_2) in DMF.

Table S5. Free radical copolymerization of VBaam (M_1) with EMI (M_2) for the determination of the monomer reactivity ratios [a)]

f [b)]	Time [min]	Conv.$_{M1}$ [%] [c)]	Conv.$_{M2}$ [%] [c)]	F [d)]
0.10	30	5.1	24.7	0.02
0.32	60	4.4	13.3	0.13
0.52	90	7.8	13.1	0.39
0.69	45	4.7	13.3	0.43
0.85	90	4.1	21.7	0.52

a) $[M_1 + M_2]_0/[AIBN]_0$ = 2000/20 mM in DMF at 60 °C. b) Actual monomer feed ratio (f = $[M_1]_0/([M_1]_0 + [M_2]_0)$) determined by ^1H NMR spectroscopy. c) Determined by ^1H NMR spectroscopy. d) Calculated from f, Conv.$_{M1}$, and Conv.$_{M2}$

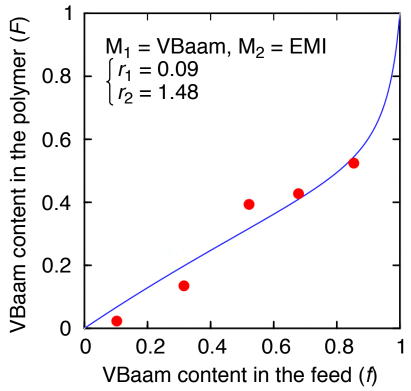

Figure S14. Copolymer composition curve for the copolymerization of VBaam (M_1) and EMI (M_2) in DMF.

Table S6. Free radical copolymerization of VBpin (M_1) with EMI (M_2) for the determination of the monomer reactivity ratios [a)]

f [b)]	Time [min]	Conv.$_{M1}$ [%] [c)]	Conv.$_{M2}$ [%] [c)]	F [d)]
0.09	20	12.4	5.0	0.19
0.31	60	9.5	10.3	0.29
0.52	60	5.2	7.7	0.42
0.71	120	8.1	18.3	0.52
0.92	100	2.8	20.4	0.60

a) $[M_1 + M_2]_0/[AIBN]_0$ = 2000/20 mM in toluene at 60 °C. b) Actual monomer feed ratio ($f = [M_1]_0/([M_1]_0 + [M_2]_0)$) determined by ^{1}H NMR spectroscopy. c) Determined by ^{1}H NMR spectroscopy. d) Calculated from f, Conv.$_{M1}$, and Conv.$_{M2}$

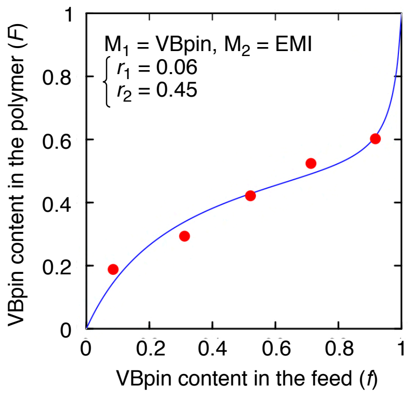

Figure S15. Copolymer composition curve for the copolymerization of VBpin (M_1) and EMI (M_2) in toluene.

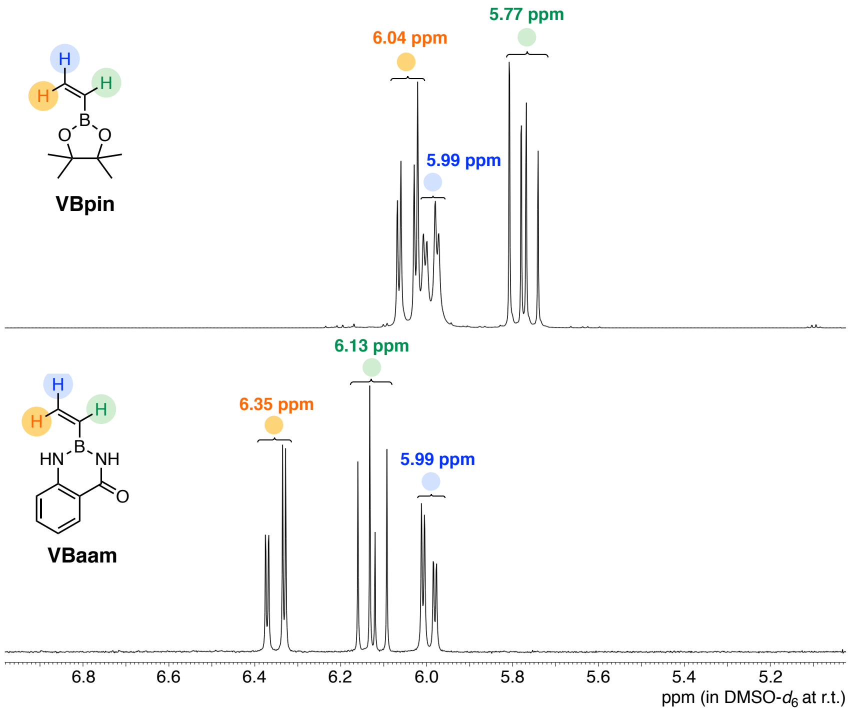

Figure S16. Comparison between the olefin peaks in the ¹H NMR spectra (in DMSO-d_6 at r.t.) of VBpin (top) and VBaam (bottom).

6 Results of Free Radical Copolymerization of VBaam

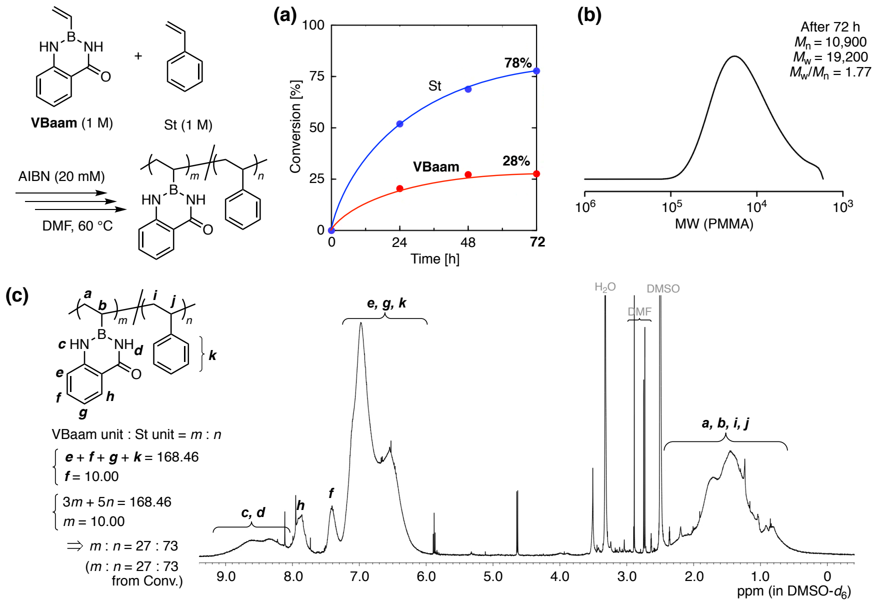

Figure S17. Radical copolymerization of VBaam with St in DMF: (a) Time–conversion curves, (b) SEC trace of the resulting copolymer, and (c) ^{1}H NMR spectrum (in DMSO-d_6 at r.t.) of poly(VBaam-*co*-St) after purification.

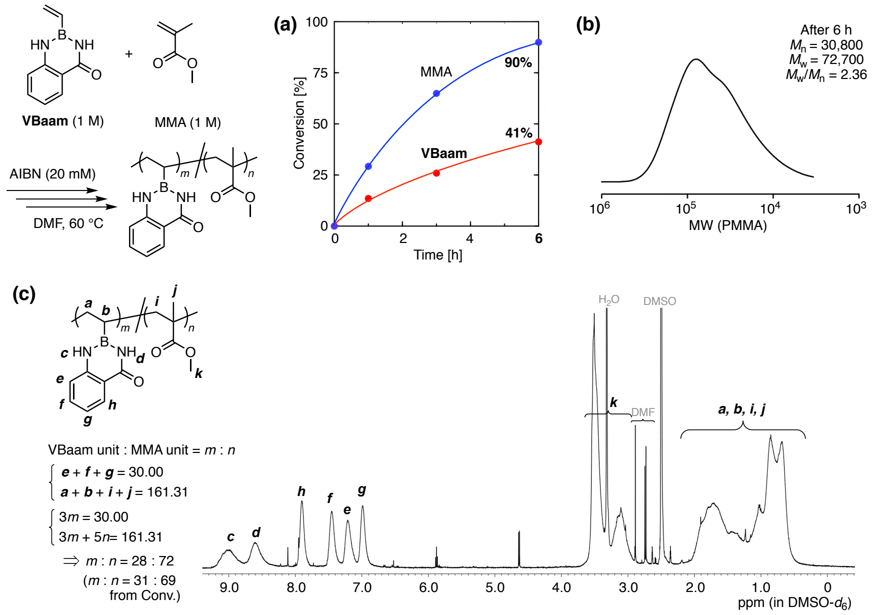

Figure S18. Radical copolymerization of VBaam with MMA in DMF: (a) Time–conversion curves, (b) SEC trace of the resulting copolymer, and (c) ^{1}H NMR spectrum (in DMSO-d_6 at r.t.) of poly(VBaam-*co*-MMA) after purification.

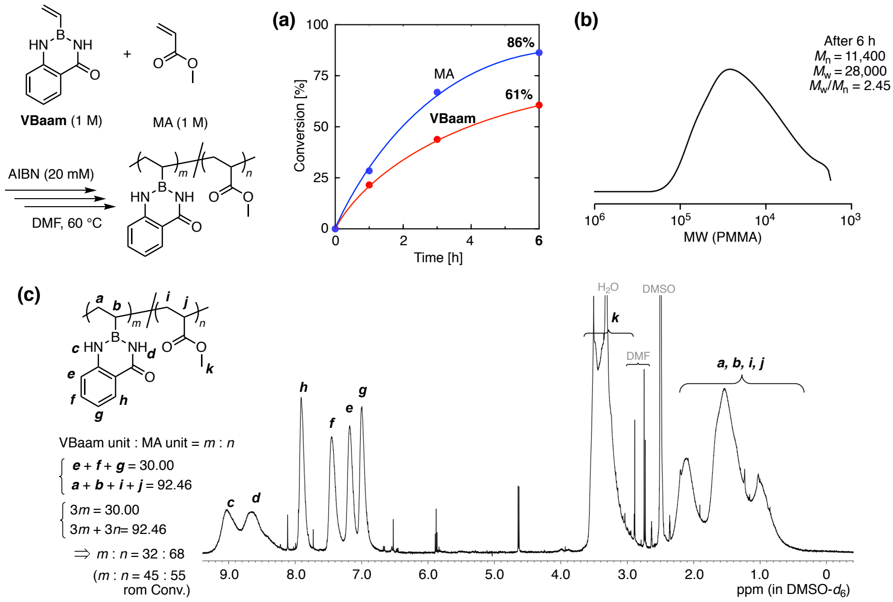

Figure S19. Radical copolymerization of VBaam with MA in DMF: (a) Time–conversion curves, (b) SEC trace of the resulting copolymer, and (c) ^{1}H NMR spectrum (in DMSO-d_6 at r.t.) of poly(VBaam-*co*-MA) after purification.

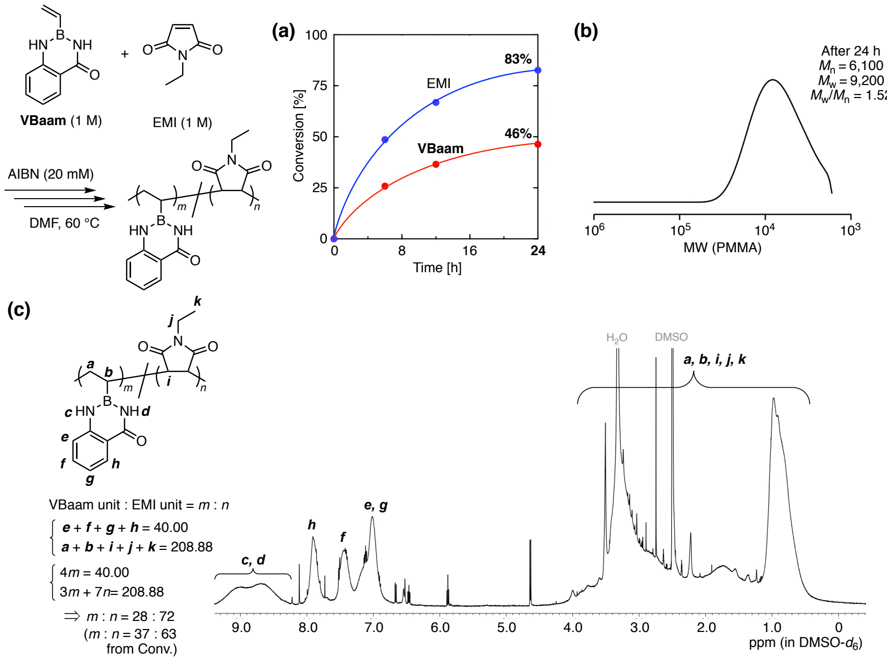

Figure S20. Radical copolymerization of VBaam with EMI in DMF: (a) Time–conversion curves, (b) SEC trace of the resulting copolymer, and (c) ^{1}H NMR spectrum (in DMSO-d_6 at r.t.) of poly(VBaam-*co*-EMI) after purification.

7 Side-chain Replacement of Poly(VBaam-*co*-St)

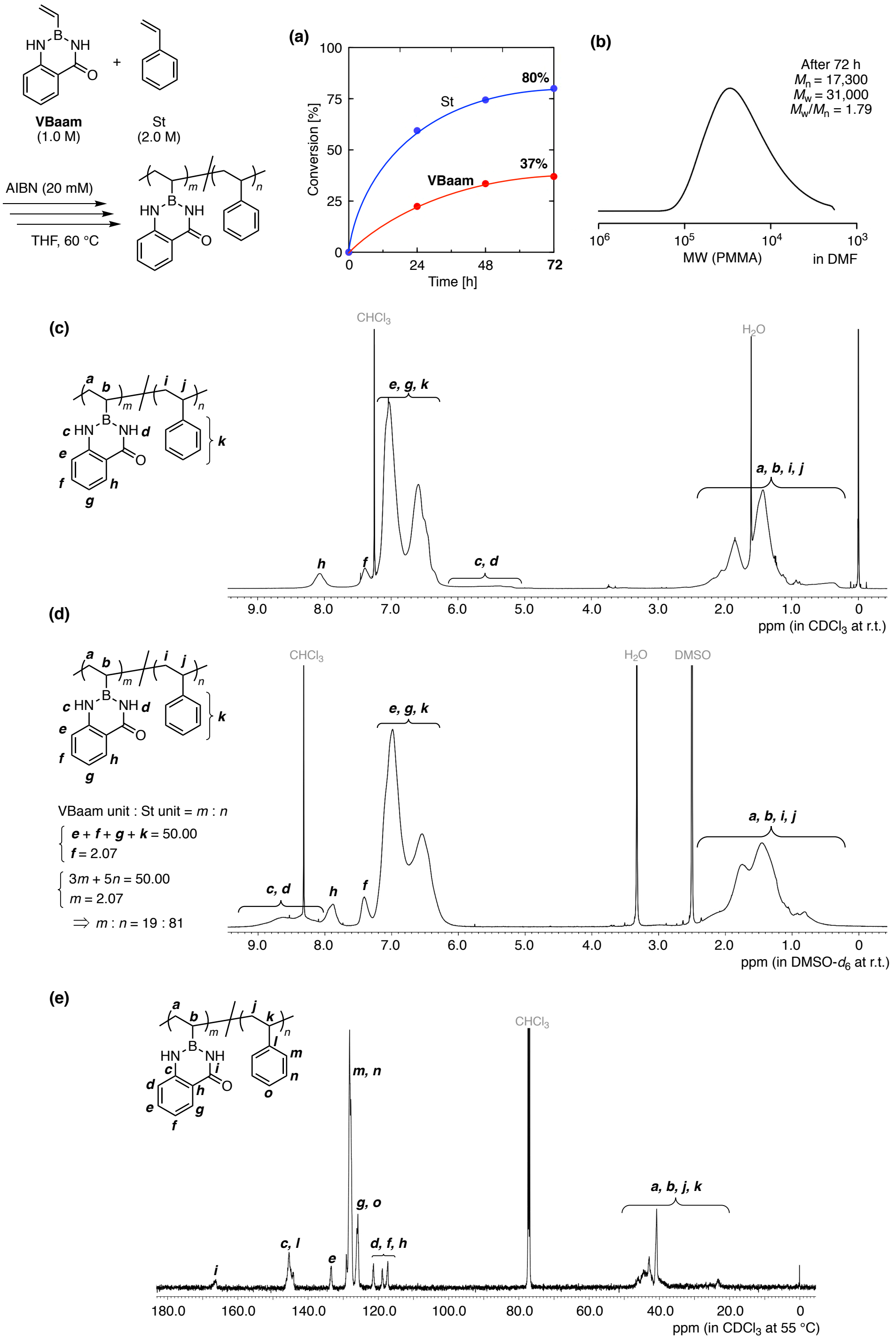

Figure S21. Synthesis of poly(VBaam-*co*-St) via free radical copolymerization as the substrate of polymer reaction: $[VBaam]_0/[St]_0/[AIBN]_0$ = 1000/2000/20 mM in THF at 60 °C. (a) Time–conversion curves and the (b) SEC trace, (c) ^{1}H NMR spectrum (in $CDCl_3$ at r.t.), (d) ^{1}H NMR spectrum (in DMSO-d_6 at r.t.), and (e) ^{13}C NMR spectrum (in $CDCl_3$ at 55 °C) of the resulting polymer.

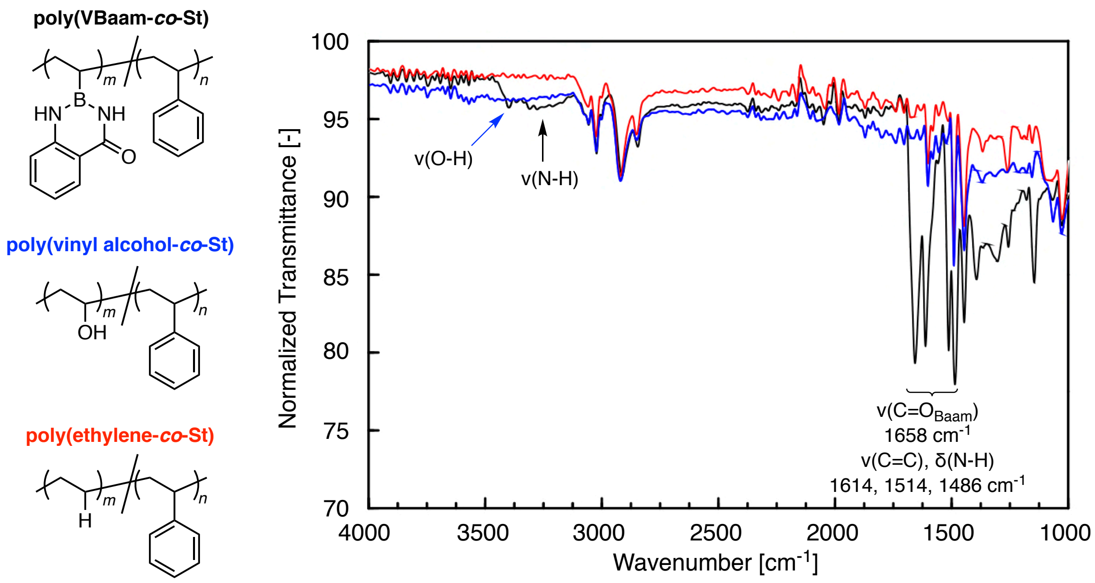

Figure S22. IR spectra of poly(VBaam-*co*-St) (black line), poly(vinyl alcohol-*co*-St) (blue line), and poly(ethylene-*co*-St) (red line).

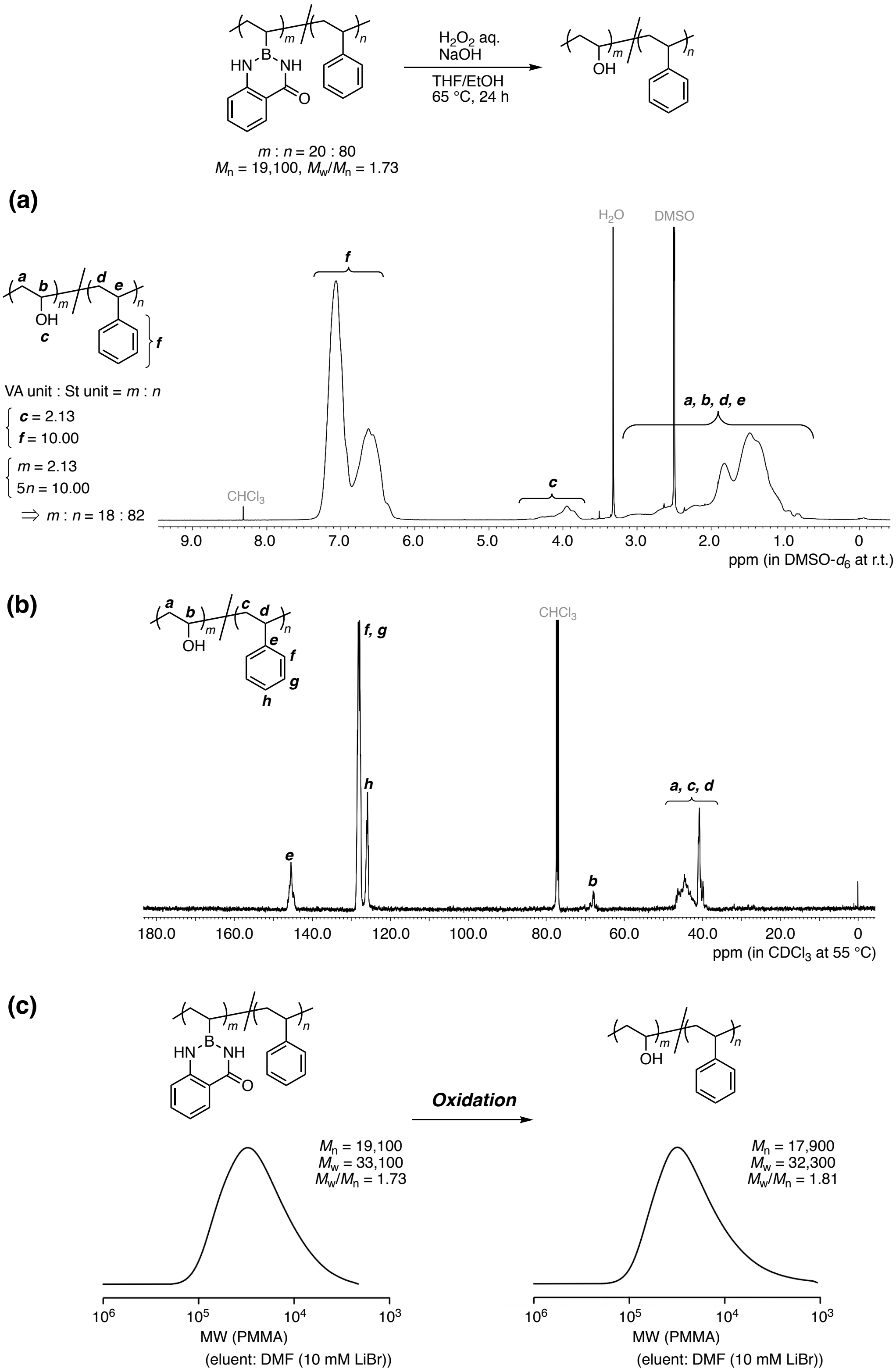

Figure S23. Oxidation of the -Baam units in poly(VBaam-*co*-St): (a) ^{1}H NMR spectrum (in DMSO-d_6 at r.t.) and (b) ^{13}C NMR spectrum (in $CDCl_3$ at 55 °C) of the resulting polymer. (c) Changes in the SEC trace upon oxidation.

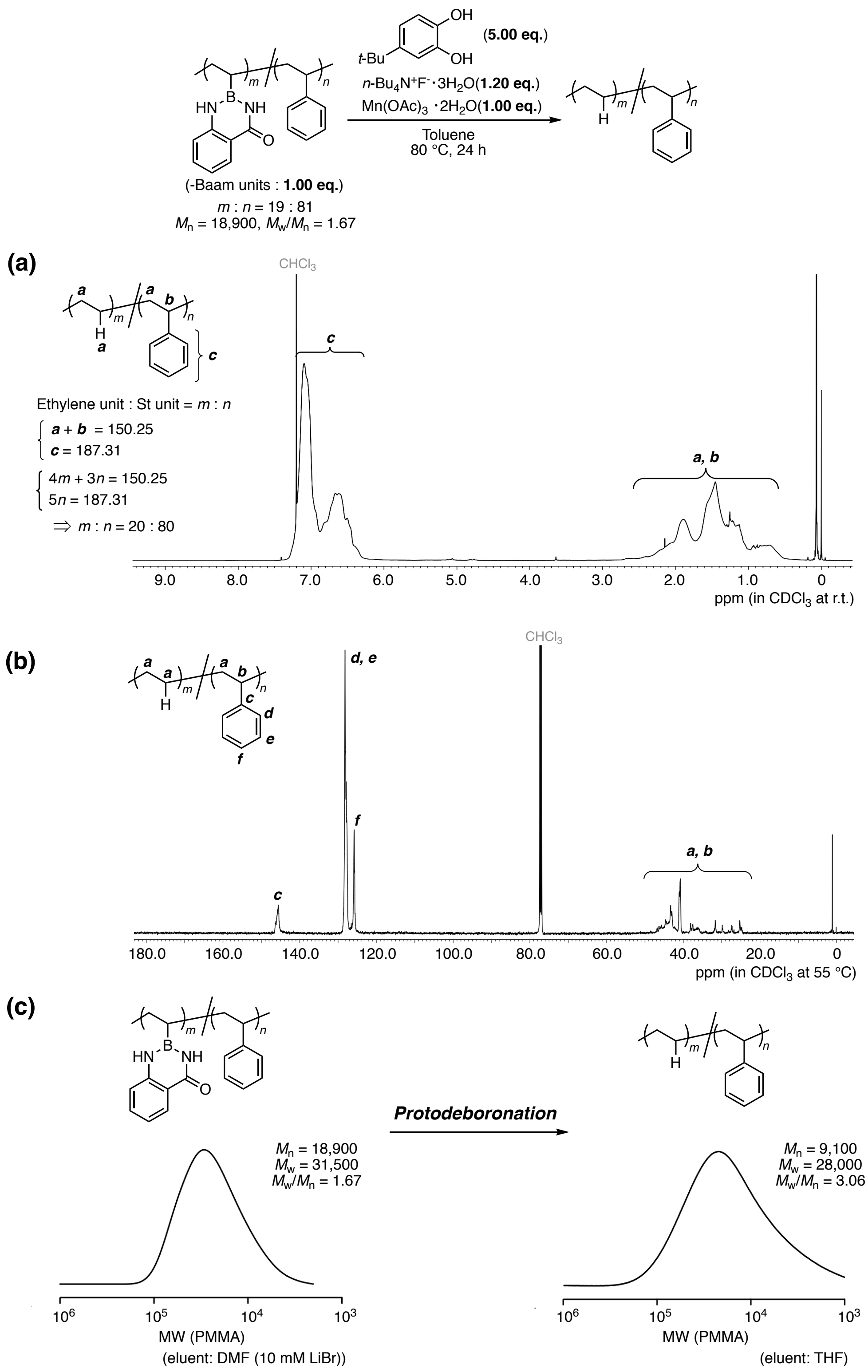

Figure S24. Protodeboronation of -Baam units in poly(VBaam-*co*-St): (a) ^{1}H NMR spectrum (in $CDCl_3$ at r.t.), and (b) ^{13}C NMR spectrum (in $CDCl_3$ at 55 °C) of the resulting polymer. (c) Changes in the SEC trace upon protodeboronation.

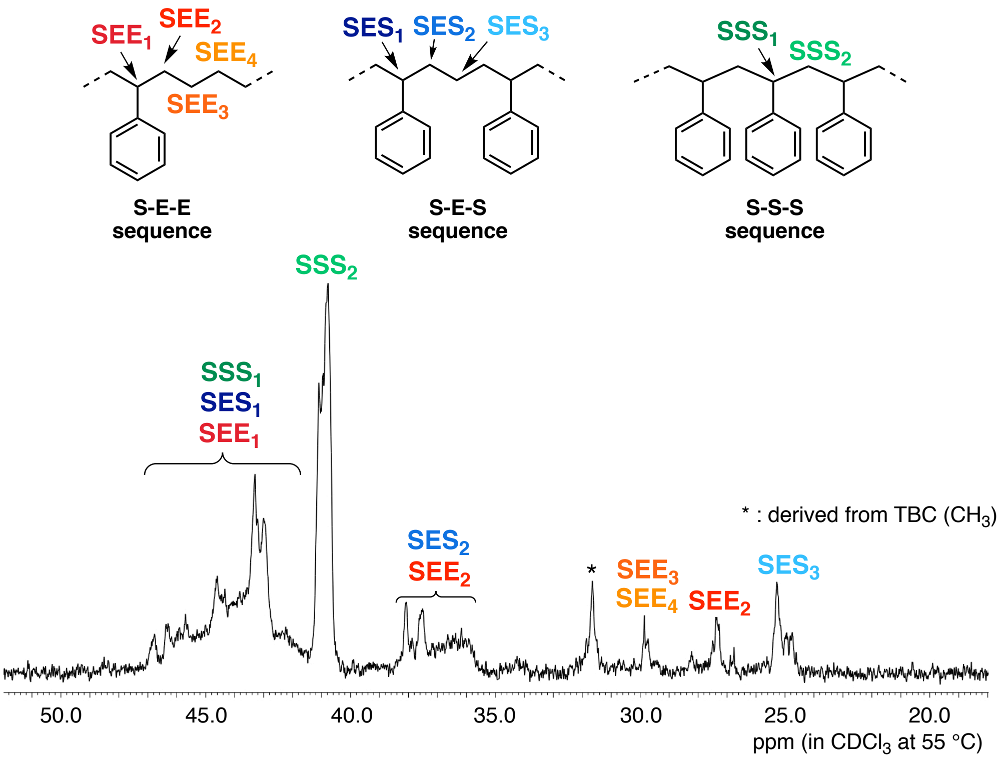

Figure S25. Sequence analysis of poly(ethylene-*co*-St) by ^{13}C NMR spectroscopy (in $CDCl_3$ at 55 °C). Peak identification was based on literature precedents.[6]

8 DSC Measurements of St-containing Polymers

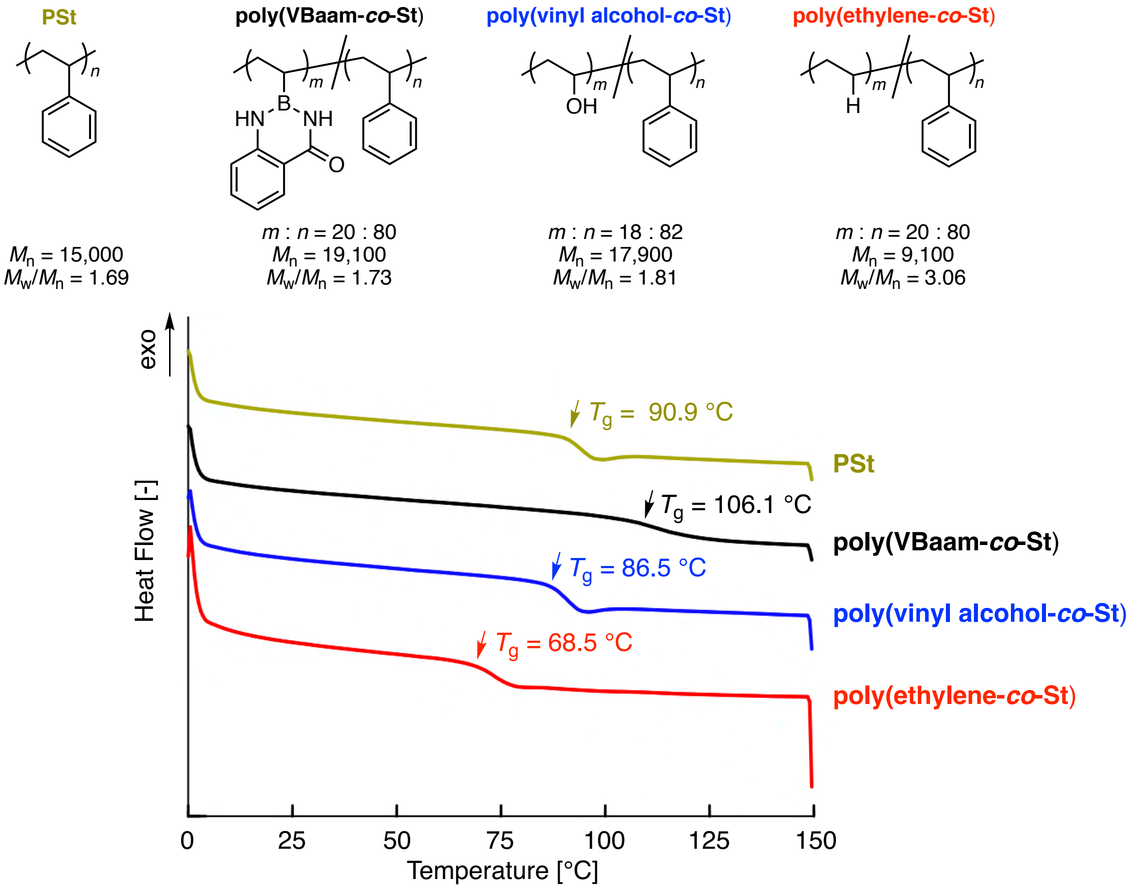

Figure S26. DSC curves (2nd heating process from 0 °C to 150 °C at 10 °C/min) of PSt, poly(VBaam-*co*-St), poly(vinyl alcohol-*co*-St), and poly(ethylene-*co*-St).

9 Reactivity of Poly(VBpin-*co*-St) in the Protodeboronation

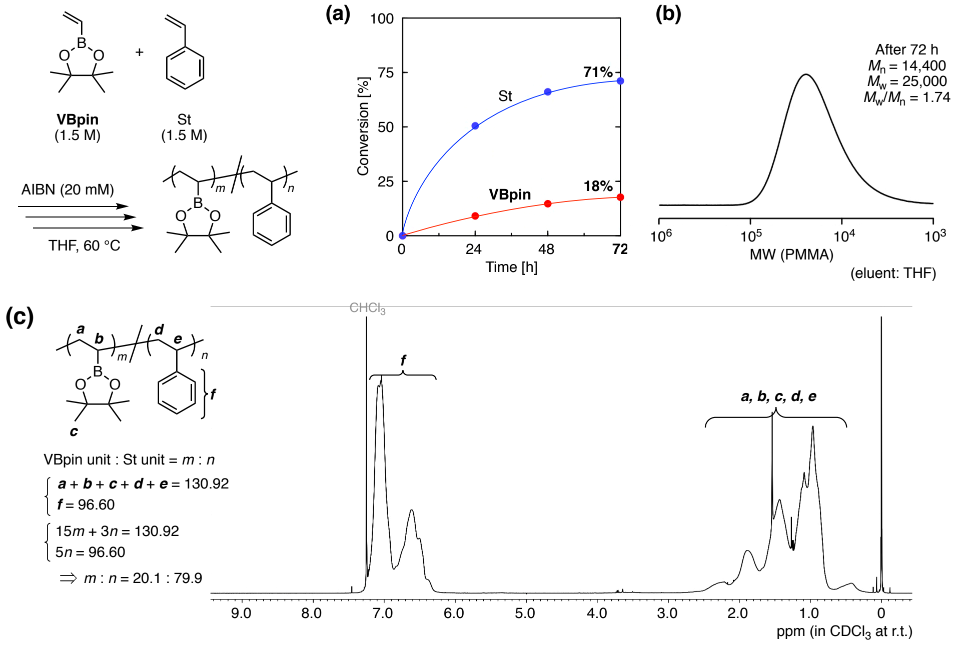

Figure S27. Synthesis of poly(VBpin-*co*-St) via free radical copolymerization of VBpin and St for the subsequent protodeboronation: $[\text{VBpin}]_0/[\text{St}]_0/[\text{AIBN}]_0$ = 1500/1500/20 mM in THF at 60 °C. (a) Time–conversion curves of the copolymerization as well as the (b) SEC trace and (c) ^{1}H NMR spectrum (in $CDCl_3$ at r.t.) of the resulting polymer.

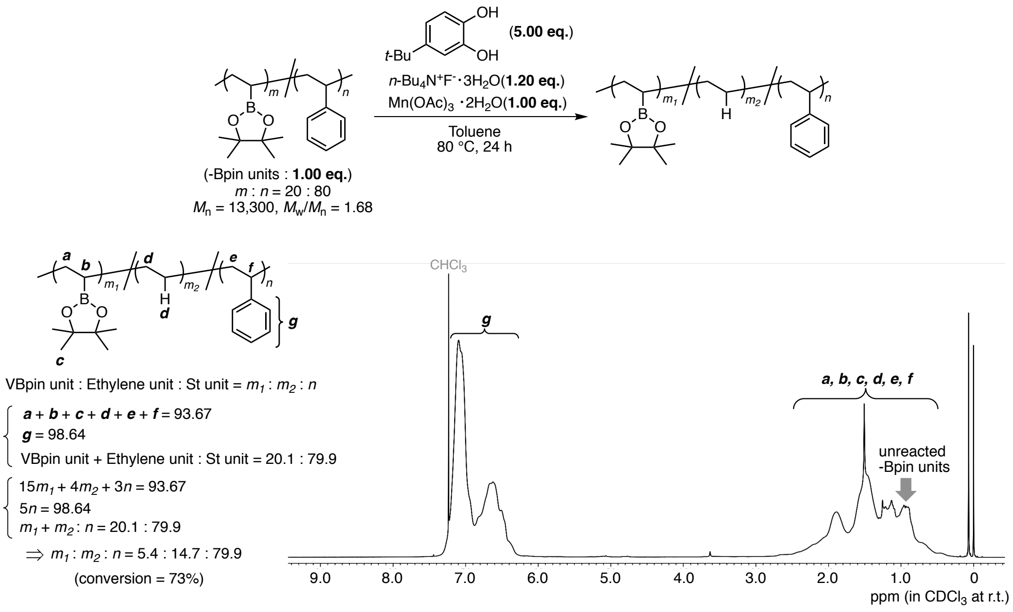

Figure S28. ^{1}H NMR spectrum (in $CDCl_3$ at r.t.) after protodeboronation of the -Bpin units in poly(VBpin-*co*-St).

10 RAFT Copolymerization of VBaam with TBA and Protodeboronation

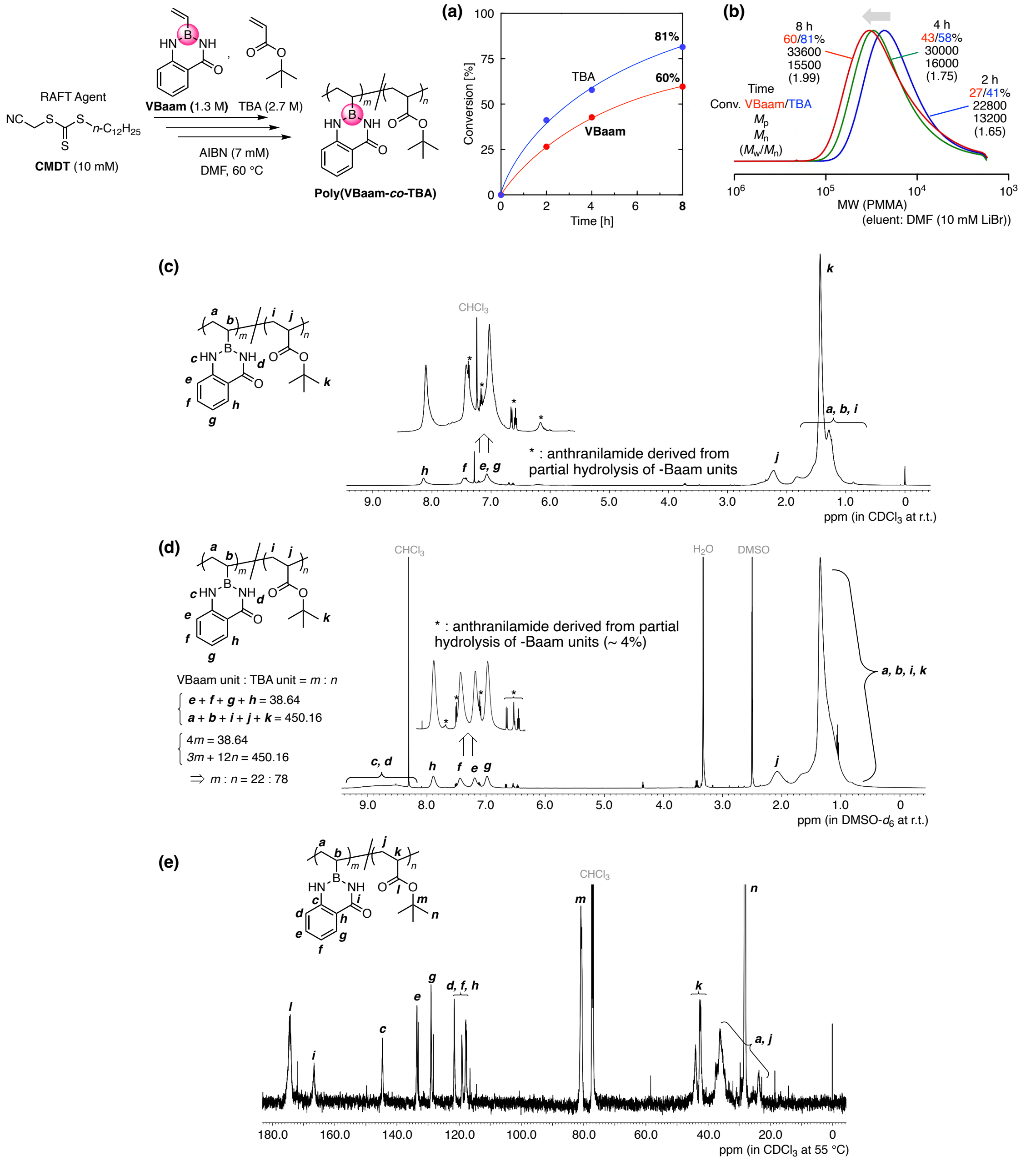

Figure S29. RAFT copolymerization of VBaam with TBA ($[VBaam]_0/[TBA]_0/[CMDT]_0/[AIBN]_0$ = 1333/2667/10/7 mM in DMF at 60 °C) for the subsequent protodeboronation: (a) Time–conversion curves. (b) SEC traces during the polymerization. (c) ^{1}H NMR spectrum (in $CDCl_3$ at r.t.), (d) ^{1}H NMR spectrum (in DMSO-d_6 at r.t.), and (e) ^{13}C NMR spectrum (in $CDCl_3$ at 55 °C) of the resulting polymer.

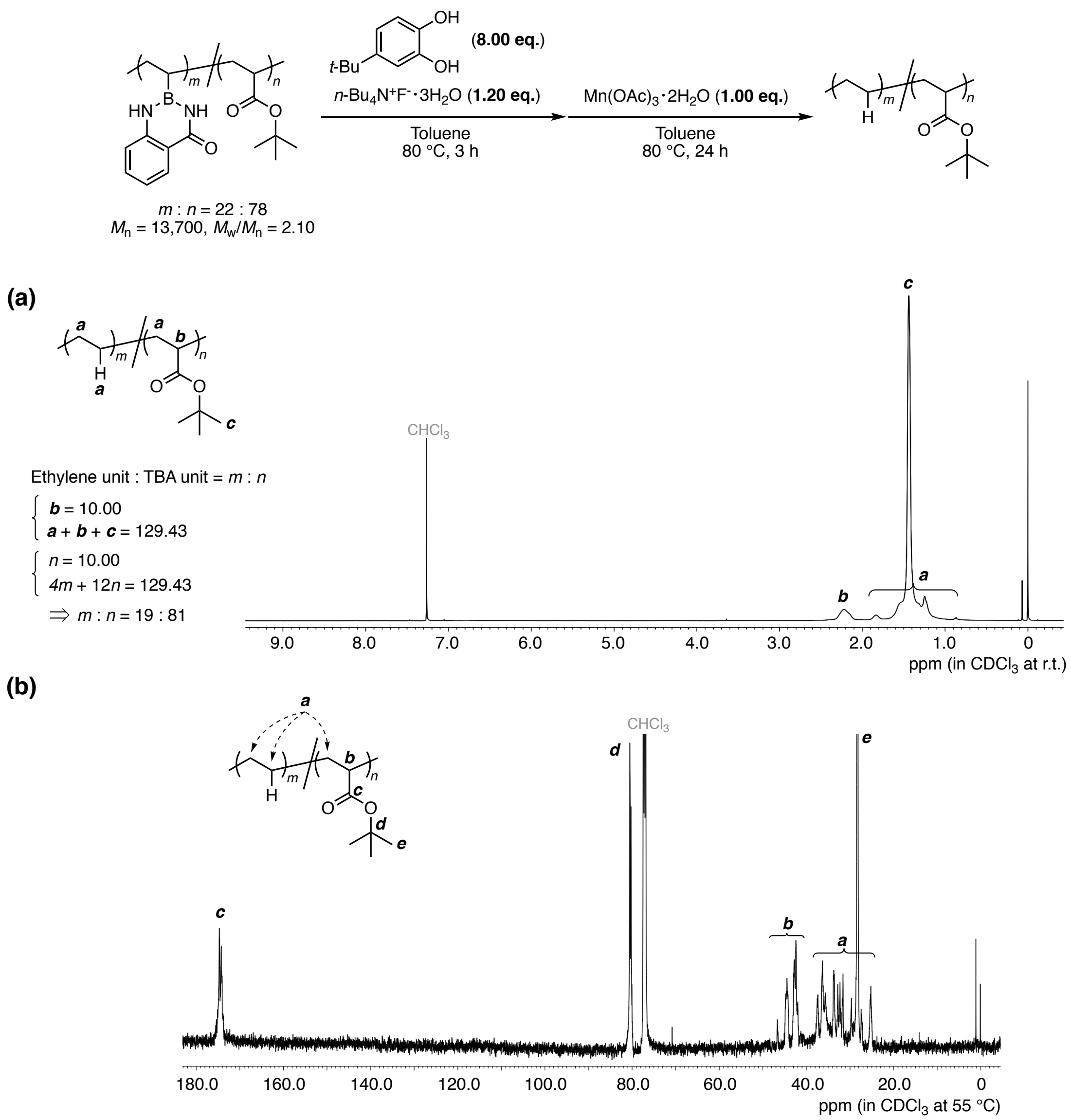

Figure S30. Protodeboronation of the -Baam units in poly(VBaam-*co*-TBA): (a) ^{1}H NMR spectrum (in $CDCl_3$ at r.t.) and (b) ^{13}C NMR spectrum (in $CDCl_3$ at 55 °C) of the resulting polymer.

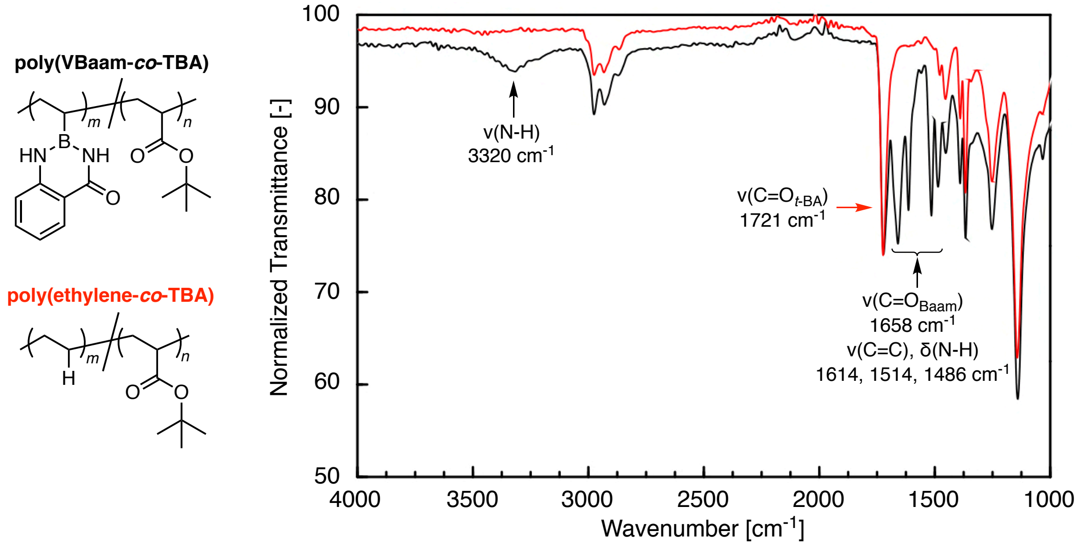

Figure S31. IR spectra of poly(VBaam-*co*-TBA) (black line) and poly(ethylene-*co*-TBA) (red line).

11 Computational Study for the Radical Polymerization Behavior

Evaluation of the stability of the chain-growth radical and the structure of each vinyl compound: The Gaussian 16A.03 program package[7] was used for all computations. Density functional theory (DFT) was applied for the optimization of the structures and vibrational analysis at the (U)B3LYP/6-31G(d) level. The nature of the optimized stationary points was proven by the results of vibrational analysis; the number of imaginary frequencies was 0. Cartesian coordinates of the optimized structures and sums of the electronic and thermal energies are shown for each species.

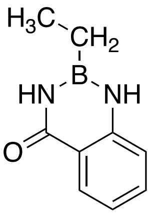

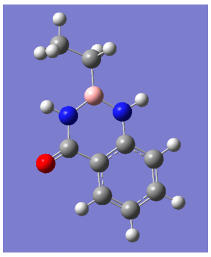

Center Number	Atomic Number	Atomic Type	Coordinates (Angstroms) X	Y	Z
1	6	0	-0.808649	-0.856685	0.000003
2	7	0	0.492595	-1.347534	0.000013
3	6	0	-1.021161	0.540324	0.000003
4	6	0	-1.918141	-1.719521	-0.000007
5	6	0	-3.206838	-1.201189	-0.000018
6	6	0	-2.330372	1.041692	-0.000007
7	6	0	-3.421933	0.184280	-0.000018
8	1	0	-1.757893	-2.795600	-0.000007
9	1	0	-4.053906	-1.882234	-0.000026
10	6	0	0.113685	1.493939	0.000013
11	1	0	-2.452727	2.120044	-0.000007
12	1	0	-4.432626	0.581067	-0.000026
13	7	0	1.374487	0.897595	0.000023
14	8	0	-0.018903	2.712127	0.000012
15	5	0	1.648386	-0.508618	0.000025

16	1	0	2.119206	1.586185	0.000027
17	1	0	0.578802	-2.355829	0.000011
18	6	0	3.115251	-1.108286	0.000048
19	1	0	3.221406	-1.772582	-0.871581
20	6	0	4.262363	-0.084859	-0.000076
21	1	0	3.221443	-1.772408	0.871806
22	1	0	4.224323	0.562021	-0.885318
23	1	0	4.224372	0.562181	0.885053
24	1	0	5.242087	-0.575359	-0.000055

Sum of electronic and thermal energies = -559.088884

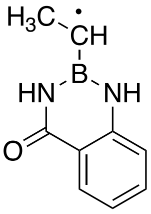

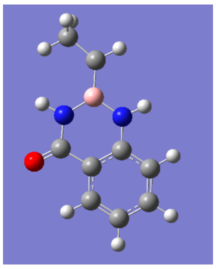

Center Number	Atomic Number	Atomic Type	Coordinates (Angstroms) X	Y	Z
1	6	0	0.764661	-0.860439	0.000000
2	7	0	-0.540490	-1.328948	0.000001
3	6	0	1.000470	0.534137	0.000000
4	6	0	1.861043	-1.741821	0.000000
5	6	0	3.157675	-1.244877	-0.000001
6	6	0	2.317221	1.012719	0.000000
7	6	0	3.394982	0.137099	-0.000001
8	1	0	1.682699	-2.815047	0.000000
9	1	0	3.993644	-1.939491	-0.000001
10	6	0	-0.118008	1.509919	0.000000
11	1	0	2.457369	2.088881	0.000000
12	1	0	4.411977	0.517476	-0.000001

13	7	0	-1.386626	0.940932	0.000000
14	8	0	0.042663	2.725645	0.000000
15	5	0	-1.688262	-0.468576	0.000001
16	1	0	-2.114611	1.646885	0.000000
17	1	0	-0.645348	-2.335344	0.000001
18	6	0	-3.110915	-1.033910	0.000001
19	1	0	-3.246218	-2.118057	0.000002
20	6	0	-4.385725	-0.252347	-0.000002
21	1	0	-5.005234	-0.493089	0.877969
22	1	0	-4.227665	0.831802	0.000010
23	1	0	-5.005219	-0.493071	-0.877987

Sum of electronic and thermal energies = -558.448605

H•

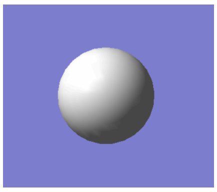

Center Number	Atomic Number	Atomic Type	Coordinates (Angstroms) X	Y	Z
1	1	0	0.000000	0.000000	0.000000

Sum of electronic and thermal energies = -0.498857

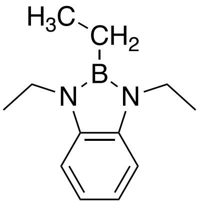

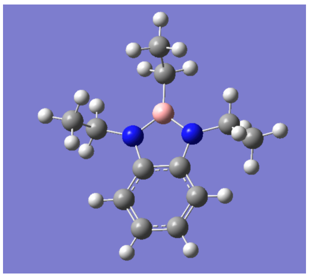

Center Number	Atomic Number	Atomic Type	Coordinates (Angstroms) X	Y	Z
1	6	0	-1.009085	0.589558	-0.155703
2	7	0	0.256982	1.181122	-0.123092
3	6	0	-0.873403	-0.779896	0.180595
4	6	0	-2.259419	1.126639	-0.454519
5	6	0	-3.375729	0.282392	-0.412638
6	6	0	-1.988565	-1.613932	0.223416
7	6	0	-3.242720	-1.067472	-0.075386
8	1	0	-2.372358	2.175389	-0.713399
9	1	0	-4.357297	0.686633	-0.644244
10	7	0	0.476578	-1.043428	0.434030
11	1	0	-1.893985	-2.664895	0.480533
12	1	0	-4.121601	-1.705599	-0.045965
13	6	0	0.953626	-2.373327	0.783377
14	5	0	1.223192	0.177490	0.250543
15	6	0	0.461277	2.599258	-0.380619
16	6	0	2.786435	0.377392	0.393077
17	1	0	3.000941	1.378409	0.793082
18	1	0	3.191793	-0.324096	1.135620
19	6	0	3.558635	0.197330	-0.933551
20	1	0	3.416785	-0.808872	-1.345404
21	1	0	3.217008	0.907888	-1.695702
22	1	0	4.636302	0.350130	-0.798033
23	1	0	1.514149	2.743552	-0.642248
24	1	0	-0.122910	2.889183	-1.264054
25	6	0	0.095666	3.496115	0.808218
26	1	0	-0.951916	3.363696	1.097073
27	1	0	0.717163	3.256426	1.677836
28	1	0	0.250597	4.551407	0.554501
29	1	0	1.929896	-2.262461	1.265635
30	1	0	0.280115	-2.807090	1.534535
31	6	0	1.074953	-3.318420	-0.418231
32	1	0	0.112573	-3.441119	-0.925366

33	1	0	1.791821	-2.926432	-1.147806
34	1	0	1.420025	-4.307282	-0.094090

Sum of electronic and thermal energies = -602.872109

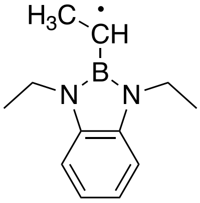

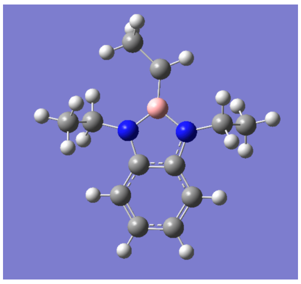

Center Number	Atomic Number	Atomic Type	Coordinates (Angstroms) X	Y	Z
1	6	0	-1.173489	0.337473	-0.163490
2	7	0	-0.135734	1.253604	-0.271824
3	6	0	-0.625100	-0.934428	0.147520
4	6	0	-2.551846	0.498861	-0.315134
5	6	0	-3.376305	-0.618896	-0.154899
6	6	0	-1.456743	-2.044154	0.306587
7	6	0	-2.836222	-1.872773	0.153802
8	1	0	-2.979448	1.469211	-0.549311
9	1	0	-4.451004	-0.508801	-0.270494
10	7	0	0.757841	-0.816121	0.249272
11	1	0	-1.047951	-3.023018	0.538641
12	1	0	-3.495026	-2.728300	0.274239
13	5	0	1.125483	0.567876	-0.020181
14	6	0	1.591860	-1.961244	0.587644
15	6	0	-0.370225	2.659239	-0.566223
16	6	0	2.488510	1.241729	-0.056937
17	1	0	2.500757	2.330061	-0.146747
18	6	0	3.861085	0.643151	0.024527
19	1	0	4.318763	0.809111	1.015059

20	1	0	3.876846	-0.435191	-0.159254
21	1	0	4.544170	1.109981	-0.699557
22	1	0	0.546753	3.069297	-0.999518
23	1	0	-1.142235	2.735348	-1.342963
24	6	0	-0.778593	3.478300	0.664108
25	1	0	-1.694704	3.083619	1.115575
26	1	0	0.010491	3.450793	1.423114
27	1	0	-0.955639	4.524336	0.387374
28	1	0	2.503563	-1.590548	1.062741
29	1	0	1.071827	-2.561425	1.345110
30	6	0	1.944701	-2.837241	-0.620196
31	1	0	1.040960	-3.216763	-1.108148
32	1	0	2.511363	-2.264705	-1.362539
33	1	0	2.552545	-3.694876	-0.308407

Sum of electronic and thermal energies = -602.231736

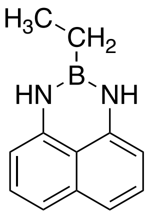

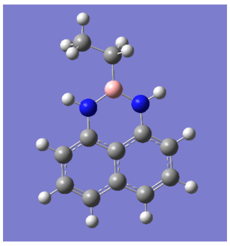

Center Number	Atomic Number	Atomic Type	Coordinates (Angstroms) X	Y	Z
1	6	0	1.182326	-2.392553	-0.000192
2	1	0	0.804806	-3.412576	-0.000266
3	6	0	0.290729	-1.330012	-0.000103
4	6	0	2.571181	-2.150938	-0.000186
5	6	0	0.782075	0.012826	-0.000006
6	6	0	3.072900	-0.867893	-0.000093
7	6	0	2.193312	0.248351	0.000000
8	6	0	-0.117754	1.123520	0.000086

9	6	0	2.662108	1.590088	0.000098
10	6	0	0.379260	2.418129	0.000180
11	6	0	1.771365	2.640964	0.000185
12	7	0	-1.093207	-1.520480	-0.000106
13	1	0	3.251613	-2.998780	-0.000256
14	1	0	4.145135	-0.691732	-0.000089
15	7	0	-1.489709	0.854971	0.000078
16	1	0	3.733463	1.771709	0.000102
17	1	0	-0.309463	3.259913	0.000250
18	1	0	2.139755	3.663732	0.000260
19	1	0	-2.087370	1.671015	0.000145
20	5	0	-2.046626	-0.456611	-0.000016
21	6	0	-3.608416	-0.734915	-0.000023
22	1	0	-1.392820	-2.486972	-0.000176
23	1	0	-3.852904	-1.361226	-0.871815
24	1	0	-3.852889	-1.361352	0.871683
25	6	0	-4.514040	0.507141	0.000075
26	1	0	-4.340036	1.131365	0.885756
27	1	0	-4.340051	1.131494	-0.885517
28	1	0	-5.575978	0.236776	0.000065

Sum of electronic and thermal energies = -599.344112

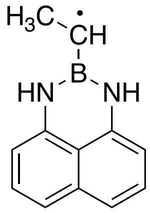

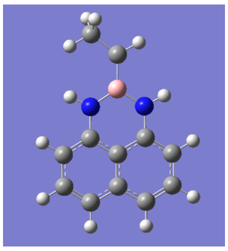

Center Number	Atomic Number	Atomic Type	Coordinates (Angstroms) X	Y	Z
1	6	0	-1.114590	-2.402099	0.000001

2	1	0	-0.718787	-3.415139	0.000001
3	6	0	-0.240832	-1.323602	-0.000001
4	6	0	-2.506882	-2.184891	0.000003
5	6	0	-0.756254	0.011717	-0.000001
6	6	0	-3.030845	-0.910615	0.000002
7	6	0	-2.171255	0.221406	0.000000
8	6	0	0.124286	1.139940	-0.000002
9	6	0	-2.665753	1.553994	0.000001
10	6	0	-0.399275	2.425327	-0.000001
11	6	0	-1.794759	2.621465	0.000000
12	7	0	1.142266	-1.490641	-0.000004
13	1	0	-3.172584	-3.044362	0.000005
14	1	0	-4.105993	-0.753221	0.000004
15	7	0	1.497614	0.900739	-0.000001
16	1	0	-3.740347	1.715123	0.000002
17	1	0	0.273200	3.280199	-0.000002
18	1	0	-2.182430	3.637122	0.000000
19	1	0	2.075344	1.730790	0.000014
20	1	0	1.459815	-2.451220	-0.000009
21	5	0	2.083170	-0.407810	-0.000004
22	6	0	3.595327	-0.658726	-0.000009
23	1	0	3.958193	-1.689192	0.000033
24	6	0	4.676964	0.374734	0.000010
25	1	0	4.294267	1.401883	0.000574
26	1	0	5.334100	0.271866	0.877774
27	1	0	5.333419	0.272596	-0.878352

Sum of electronic and thermal energies = -598.703229

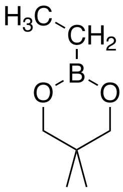 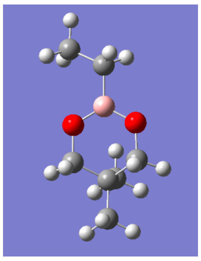

Center Number	Atomic Number	Atomic Type	Coordinates (Angstroms) X	Y	Z
1	6	0	3.540246	-0.681213	0.185970
2	6	0	2.690638	0.590233	0.042489
3	1	0	3.432366	-1.331117	-0.689181
4	1	0	4.605265	-0.446338	0.301145
5	1	0	3.233662	-1.269107	1.058880
6	1	0	3.056874	1.188018	-0.807347
7	5	0	1.140619	0.359318	-0.169616
8	1	0	2.829442	1.245124	0.914785
9	8	0	0.671757	-0.903136	-0.427563
10	8	0	0.300504	1.441937	-0.086527
11	6	0	-1.617781	-0.110058	0.067687
12	6	0	-1.100484	1.288128	-0.314197
13	6	0	-0.719913	-1.129169	-0.655563
14	1	0	-0.905509	-1.091887	-1.738450
15	1	0	-0.941534	-2.148029	-0.316467
16	6	0	-1.549219	-0.314762	1.592551
17	6	0	-3.066310	-0.270076	-0.418002
18	1	0	-1.307267	1.487688	-1.375519
19	1	0	-1.610333	2.061063	0.273072
20	1	0	-3.147513	-0.127352	-1.502428
21	1	0	-3.450722	-1.269241	-0.180602
22	1	0	-3.724596	0.460225	0.067511
23	1	0	-1.906879	-1.314862	1.864603
24	1	0	-0.529788	-0.209532	1.975494
25	1	0	-2.177716	0.419849	2.109689

Sum of electronic and thermal energies = -450.956217

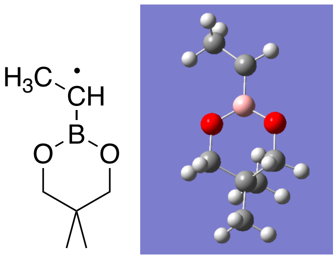

Center Number	Atomic Number	Atomic Type	Coordinates (Angstroms) X	Y	Z
1	6	0	3.691602	-0.544689	0.127289
2	6	0	2.669925	0.544611	0.089364
3	1	0	4.472076	-0.393389	-0.635790
4	1	0	4.223159	-0.566548	1.092358
5	1	0	3.244507	-1.529589	-0.035810
6	1	0	3.041324	1.558551	0.242959
7	5	0	1.165899	0.332534	-0.148903
8	8	0	0.691033	-0.948293	-0.333668
9	8	0	0.332234	1.429408	-0.152180
10	6	0	-1.596163	-0.101865	0.065020
11	6	0	-1.062551	1.266622	-0.395651
12	6	0	-0.695213	-1.170871	-0.579013
13	1	0	-0.860578	-1.192973	-1.666322
14	1	0	-0.938917	-2.166172	-0.187946
15	6	0	-1.551524	-0.210410	1.600547
16	6	0	-3.038222	-0.282099	-0.432419
17	1	0	-1.249546	1.396932	-1.471906
18	1	0	-1.580784	2.078090	0.129582
19	1	0	-3.101514	-0.209000	-1.524974
20	1	0	-3.434679	-1.261030	-0.137403
21	1	0	-3.697842	0.482816	-0.005498
22	1	0	-1.917963	-1.190081	1.929604

23	1	0	-0.537390	-0.084612	1.991082
24	1	0	-2.184609	0.557623	2.060546

Sum of electronic and thermal Energies = -450.314887

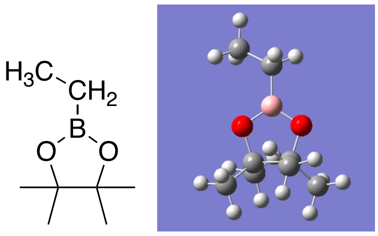

Center Number	Atomic Number	Atomic Type	Coordinates (Angstroms) X	Y	Z
1	8	0	0.146568	-1.291930	-0.396635
2	5	0	1.146260	-0.419402	-0.031854
3	6	0	-1.126278	-0.685945	-0.037433
4	8	0	0.663283	0.810202	0.349679
5	6	0	-0.758668	0.847767	0.042367
6	6	0	-2.155475	-1.055293	-1.104463
7	6	0	2.672947	-0.801304	-0.035498
8	6	0	-1.533618	-1.287599	1.314796
9	6	0	-0.897304	1.580264	-1.299316
10	6	0	-1.470108	1.633522	1.142643
11	1	0	-1.573136	-2.377179	1.219923
12	1	0	-0.805773	-1.043794	2.095254
13	1	0	-2.518810	-0.933692	1.636620
14	1	0	-1.823671	-0.765084	-2.103880
15	1	0	-2.311279	-2.138921	-1.103337
16	1	0	-3.119566	-0.574451	-0.902239
17	1	0	-1.269653	1.216250	2.132012
18	1	0	-1.119301	2.670549	1.137591
19	1	0	-2.554009	1.642025	0.979527
20	1	0	-0.435825	2.568825	-1.212575

21	1	0	-0.387281	1.039628	-2.102907
22	1	0	-1.946906	1.713329	-1.582205
23	1	0	2.852939	-1.473836	0.818063
24	1	0	2.877637	-1.419372	-0.921086
25	6	0	3.643378	0.388841	0.028624
26	1	0	3.527774	1.044394	-0.842248
27	1	0	3.459057	1.002081	0.917399
28	1	0	4.688451	0.058569	0.058689

Sum of electronic and thermal Energies = -490.251279

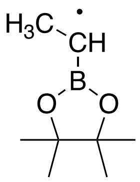

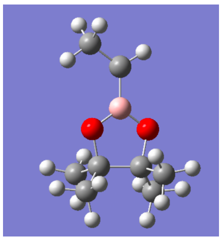

Center Number	Atomic Number	Atomic Type	Coordinates (Angstroms) X	Y	Z
1	6	0	3.760613	0.266192	0.126608
2	1	0	3.367452	1.247995	0.406271
3	1	0	4.430913	0.400456	-0.737502
4	1	0	4.404162	-0.089923	0.946934
5	6	0	2.669314	-0.709051	-0.174816
6	5	0	1.174134	-0.387403	-0.098893
7	1	0	2.985505	-1.711147	-0.465888
8	8	0	0.678280	0.843419	0.287498
9	8	0	0.175940	-1.290062	-0.407606
10	6	0	-0.749927	0.842669	0.021257
11	6	0	-1.088194	-0.699731	-0.002755
12	6	0	-2.160451	-1.115723	-1.008747
13	6	0	-0.945935	1.529676	-1.337903
14	6	0	-1.446989	1.645857	1.118361

15	6	0	-1.418183	-1.271895	1.383455
16	1	0	-0.502616	2.529482	-1.296541
17	1	0	-0.449757	0.973597	-2.139604
18	1	0	-2.006407	1.633028	-1.591401
19	1	0	-1.207329	1.263241	2.113028
20	1	0	-1.120689	2.689920	1.071828
21	1	0	-2.535178	1.625249	0.987931
22	1	0	-3.122487	-0.645937	-0.773308
23	1	0	-1.881436	-0.847201	-2.030138
24	1	0	-2.296141	-2.201456	-0.971597
25	1	0	-0.659811	-0.990107	2.120779
26	1	0	-2.395266	-0.930681	1.741895
27	1	0	-1.436836	-2.364320	1.319889

Sum of electronic and thermal Energies = -489.609552

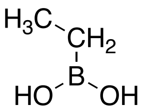

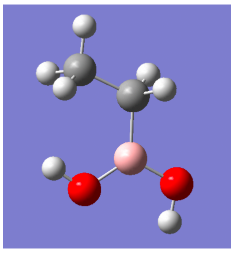

Center Number	Atomic Number	Atomic Type	Coordinates (Angstroms) X	Y	Z
1	6	0	-2.032467	0.081078	-0.000082
2	6	0	-0.770582	-0.796192	0.000111
3	1	0	-2.949937	-0.517962	-0.000195
4	1	0	-2.076064	0.728525	0.886348
5	1	0	-2.075842	0.728492	-0.886551
6	1	0	-0.783107	-1.468742	0.869897
7	1	0	-0.783096	-1.469165	-0.869340
8	5	0	0.613424	-0.022176	-0.000069

9	8	0	1.771398	-0.751580	-0.000053
10	8	0	0.729727	1.348622	0.000042
11	1	0	-0.132233	1.787034	0.000144
12	1	0	2.542454	-0.162954	-0.000040

Sum of electronic and thermal energies = -255.729322

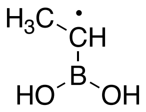

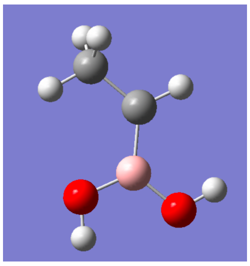

Center Number	Atomic Number	Atomic Type	Coordinates (Angstroms) X	Y	Z
1	6	0	-2.099978	0.047781	0.000111
2	6	0	-0.802127	-0.692642	0.000116
3	1	0	-2.712826	-0.212972	-0.877569
4	1	0	-2.712728	-0.212789	0.877913
5	1	0	-1.948598	1.130623	-0.000011
6	1	0	-0.887357	-1.782440	0.000235
7	5	0	0.586480	-0.018851	-0.000031
8	8	0	0.661049	1.352809	-0.000181
9	8	0	1.778310	-0.718335	-0.000020
10	1	0	1.640100	-1.674879	0.000090
11	1	0	1.586762	1.640093	-0.000262

Sum of electronic and thermal energies = -255.086811

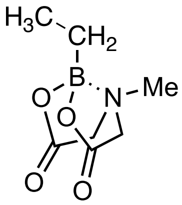

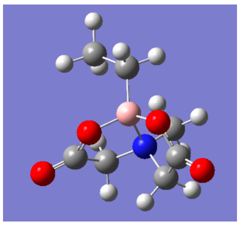

Center Number	Atomic Number	Atomic Type	Coordinates (Angstroms) X	Y	Z
1	5	0	0.094523	0.584846	-0.560526
2	8	0	-1.268016	0.729611	-1.074695
3	8	0	0.808498	-0.553941	-1.181035
4	6	0	-2.187013	-0.007380	-0.452688
5	6	0	1.288813	-1.467448	-0.341054
6	8	0	1.959997	-2.425017	-0.634886
7	8	0	-3.353681	-0.085892	-0.742833
8	6	0	-1.545808	-0.772712	0.714454
9	6	0	0.881312	-1.122495	1.101827
10	7	0	-0.217776	-0.129076	0.970735
11	6	0	-0.302451	0.826364	2.104397
12	1	0	0.645984	1.354707	2.202485
13	1	0	-1.093383	1.550771	1.898802
14	1	0	-0.528400	0.292140	3.032699
15	6	0	0.903626	1.961657	-0.562578
16	1	0	0.836737	2.328039	-1.597485
17	6	0	2.388395	1.908459	-0.164211
18	1	0	-2.180279	-0.755353	1.603023
19	1	0	-1.400663	-1.813271	0.411226
20	1	0	0.580710	-2.009694	1.664017
21	1	0	1.729883	-0.654955	1.609208
22	1	0	0.377618	2.722490	0.032488
23	1	0	2.877804	2.879863	-0.298743
24	1	0	2.535801	1.628860	0.889124
25	1	0	2.934377	1.178934	-0.772654

Sum of electronic and thermal energies = -654.378822

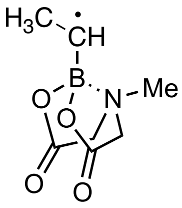

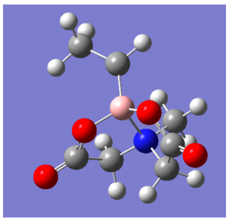

Center Number	Atomic Number	Atomic Type	Coordinates (Angstroms) X	Y	Z
1	5	0	0.170973	0.671662	-0.423343
2	8	0	-1.149554	0.948462	-0.984136
3	8	0	0.897370	-0.393318	-1.142234
4	6	0	-2.134869	0.152910	-0.566371
5	6	0	1.276617	-1.446604	-0.420419
6	8	0	1.945032	-2.370964	-0.810778
7	8	0	-3.273922	0.170515	-0.957986
8	6	0	-1.618340	-0.820270	0.505019
9	6	0	0.758364	-1.318630	1.022014
10	7	0	-0.305943	-0.288113	0.973134
11	6	0	-0.450653	0.488770	2.227193
12	1	0	0.498008	0.977429	2.453700
13	1	0	-1.213734	1.256844	2.081902
14	1	0	-0.745017	-0.167867	3.052591
15	6	0	1.009707	1.949079	-0.089974
16	1	0	0.481089	2.871313	0.160001
17	6	0	2.498053	2.073245	-0.187774
18	1	0	-2.332971	-0.922417	1.324887
19	1	0	-1.478788	-1.802016	0.043521
20	1	0	0.404721	-2.276656	1.410871
21	1	0	1.572968	-0.961810	1.659829
22	1	0	2.949489	2.433884	0.750942
23	1	0	2.981031	1.128568	-0.457791
24	1	0	2.785270	2.812648	-0.952732

Sum of electronic and thermal Energies = -653.733077

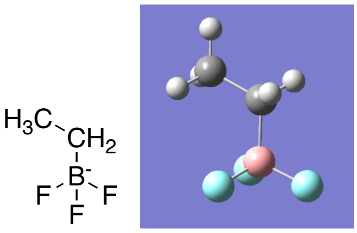

Center Number	Atomic Number	Atomic Type	Coordinates (Angstroms) X	Y	Z
1	6	0	-2.140298	-0.089576	-0.000344
2	6	0	-0.913407	0.831961	-0.000101
3	1	0	-3.108720	0.440732	-0.000455
4	1	0	-2.119970	-0.745376	-0.881267
5	1	0	-2.120230	-0.745487	0.880502
6	1	0	-0.962427	1.497770	-0.878900
7	1	0	-0.962692	1.497657	0.878766
8	5	0	0.503179	0.012719	0.000075
9	9	0	0.592621	-0.816550	1.155544
10	9	0	0.593017	-0.816403	-1.155459
11	9	0	1.601070	0.914820	0.000320

Sum of electronic and thermal energies = -403.737902

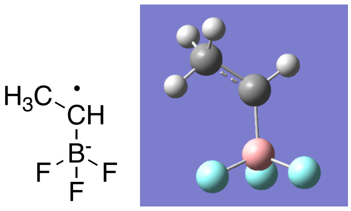

Center Number	Atomic Number	Atomic Type	Coordinates (Angstroms) X	Y	Z
1	6	0	-2.260242	0.023572	0.001789
2	6	0	-0.957806	-0.718477	-0.011899
3	5	0	0.502004	-0.020975	-0.002338
4	1	0	-1.060488	-1.810858	0.038759
5	9	0	0.361622	1.381250	-0.215518
6	9	0	1.166717	-0.222038	1.242640
7	9	0	1.330489	-0.561015	-1.026084
8	1	0	-2.955104	-0.283663	-0.805505
9	1	0	-2.836799	-0.113905	0.940266
10	1	0	-2.078797	1.098966	-0.110507

Sum of electronic and thermal energies = -403.085722

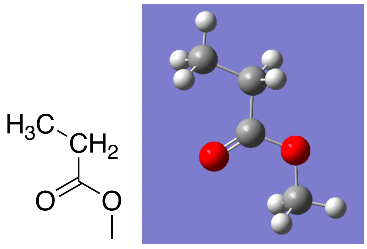

Center Number	Atomic Number	Atomic Type	Coordinates (Angstroms) X	Y	Z
1	6	0	2.482917	0.111372	0.000246
2	1	0	2.520286	0.758273	-0.881243
3	1	0	3.373406	-0.525625	0.000316
4	1	0	2.519867	0.757890	0.882038
5	6	0	1.216682	-0.742668	-0.000238
6	6	0	-0.052189	0.088344	-0.000094
7	1	0	1.181012	-1.406452	-0.873743
8	1	0	1.180790	-1.407176	0.872701
9	8	0	-1.148653	-0.706157	0.000089

10	8	0	-0.105803	1.298973	-0.000159
11	6	0	-2.408027	-0.015591	0.000109
12	1	0	-2.503028	0.613371	0.889515
13	1	0	-2.502719	0.613992	-0.888884
14	1	0	-3.170263	-0.795544	-0.000276

Sum of electronic and thermal Energies = -307.576676

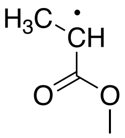

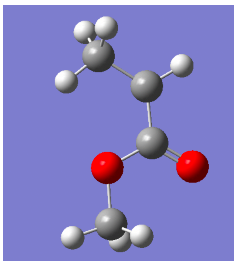

Center Number	Atomic Number	Atomic Type	Coordinates (Angstroms) X	Y	Z
1	6	0	-2.238696	-0.694611	-0.000001
2	1	0	-1.650618	-1.613874	0.000093
3	1	0	-2.901586	-0.701675	-0.878383
4	1	0	-2.901738	-0.701579	0.878265
5	6	0	-1.373172	0.514608	0.000006
6	6	0	0.075637	0.520276	0.000001
7	1	0	-1.824248	1.502158	0.000002
8	8	0	0.617097	-0.731305	0.000002
9	8	0	0.756192	1.538764	-0.000003
10	6	0	2.048941	-0.768984	-0.000001
11	1	0	2.450119	-0.272826	-0.888574
12	1	0	2.450123	-0.272814	0.888564
13	1	0	2.315378	-1.826802	0.000006

Sum of electronic and thermal energies = -306.934149

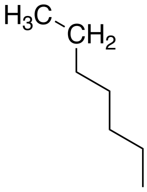

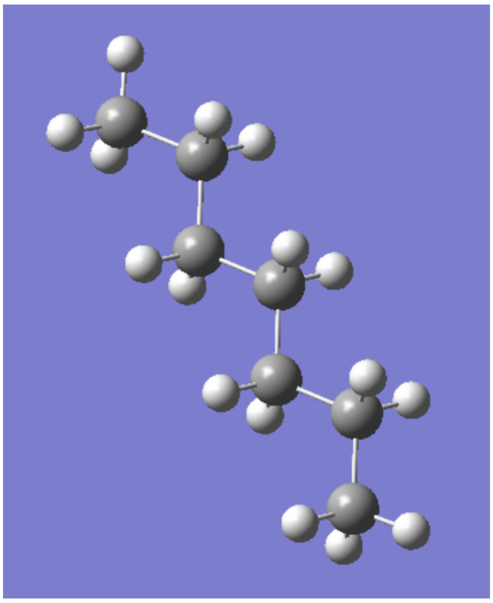

Center Number	Atomic Number	Atomic Type	Coordinates (Angstroms) X	Y	Z
1	6	0	-3.844346	-0.352265	-0.000106
2	6	0	-2.567710	0.494787	0.000207
3	1	0	-4.742805	0.275610	0.000435
4	1	0	-3.890689	-0.998938	-0.885117
5	1	0	-3.890390	-1.000027	0.884126
6	1	0	-2.567089	1.156647	-0.877618
7	1	0	-2.567125	1.156074	0.878461
8	6	0	-1.283730	-0.345157	-0.000040
9	1	0	-1.284813	-1.007836	0.878385
10	1	0	-1.284952	-1.007555	-0.878678
11	6	0	0.000008	0.494844	-0.000021
12	1	0	0.000015	1.157215	-0.878595
13	1	0	-0.000047	1.157279	0.878505
14	6	0	1.283723	-0.345132	0.000052
15	1	0	1.284944	-1.007515	0.878706
16	1	0	1.284840	-1.007849	-0.878349
17	6	0	2.567726	0.494795	-0.000186

18	1	0	2.567131	1.156121	-0.878412
19	1	0	2.567107	1.156626	0.877663
20	6	0	3.844335	-0.352276	0.000095
21	1	0	3.890675	-0.998976	0.885085
22	1	0	3.890359	-1.000020	-0.884154
23	1	0	4.742811	0.275574	-0.000442

Sum of electronic and thermal energies = -276.170770

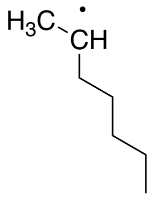

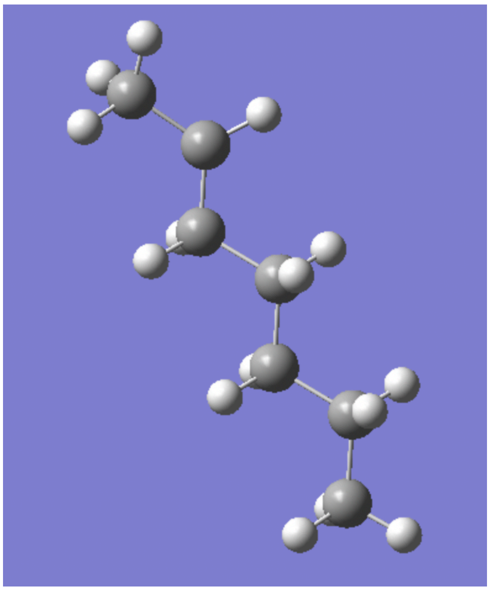

Center Number	Atomic Number	Atomic Type	Coordinates (Angstroms) X	Y	Z
1	6	0	3.914261	-0.223925	-0.138300
2	6	0	2.587415	0.424625	0.079286
3	1	0	4.688242	0.506723	-0.400178
4	1	0	4.273163	-0.757277	0.761715
5	1	0	3.870674	-0.974634	-0.939906
6	1	0	2.564981	1.442904	0.465024
7	6	0	1.321637	-0.369984	0.122615
8	1	0	1.341891	-1.140274	-0.665464

9	1	0	1.259365	-0.940977	1.071108
10	6	0	0.047767	0.478644	-0.019138
11	1	0	0.036947	1.243770	0.771140
12	1	0	0.085020	1.025458	-0.971782
13	6	0	-1.245331	-0.342168	0.051517
14	1	0	-1.233780	-1.108663	-0.737970
15	1	0	-1.276854	-0.890687	1.005032
16	6	0	-2.517311	0.504281	-0.086334
17	1	0	-2.529760	1.269624	0.702805
18	1	0	-2.485974	1.051735	-1.039135
19	6	0	-3.805008	-0.322891	-0.015169
20	1	0	-3.838159	-1.074253	-0.813868
21	1	0	-3.882157	-0.854854	0.941200
22	1	0	-4.694185	0.309911	-0.116583

Sum of electronic and thermal energies = -275.519330

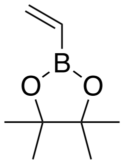

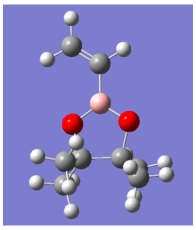

Center Number	Atomic Number	Atomic Type	Coordinates (Angstroms) X	Y	Z
1	6	0	-3.691010	0.284690	0.116658
2	1	0	-3.414414	1.303069	0.382287
3	1	0	-4.758584	0.074542	0.073922
4	6	0	-2.758725	-0.643336	-0.138386
5	5	0	-1.236966	-0.344633	-0.071981
6	1	0	-3.083590	-1.650878	-0.401541
7	8	0	-0.711585	0.872231	0.299243
8	8	0	-0.272088	-1.274108	-0.380443

9	6	0	0.715057	0.837929	0.017517
10	6	0	1.016694	-0.712274	-0.004879
11	6	0	1.359377	-1.287614	1.376304
12	6	0	1.440980	1.627347	1.105526
13	6	0	0.911395	1.517354	-1.344930
14	6	0	2.056053	-1.159528	-1.031321
15	1	0	1.137687	2.678277	1.059667
16	1	0	1.203352	1.252469	2.103561
17	1	0	2.526928	1.582023	0.963545
18	1	0	0.392272	0.972989	-2.140089
19	1	0	0.493322	2.527768	-1.300264
20	1	0	1.971034	1.594130	-1.610782
21	1	0	2.350735	-0.967550	1.714187
22	1	0	0.622187	-0.986292	2.127180
23	1	0	1.352070	-2.380351	1.316462
24	1	0	1.762988	-0.888098	-2.047951
25	1	0	3.034167	-0.713209	-0.817820
26	1	0	2.165138	-2.248111	-0.991790

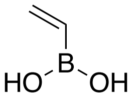

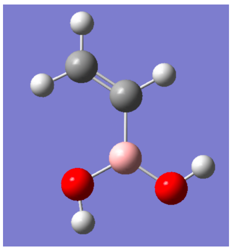

Center Number	Atomic Number	Atomic Type	Coordinates (Angstroms) X	Y	Z
1	6	0	2.047550	0.067541	0.000145
2	6	0	0.921501	-0.657906	-0.000162
3	1	0	3.038699	-0.383152	0.000218
4	1	0	2.012912	1.154748	0.000284
5	1	0	1.023404	-1.746899	-0.000314

6	5	0	-0.504334	-0.015645	-0.000280
7	8	0	-0.626548	1.347289	-0.000048
8	8	0	-1.663098	-0.759606	0.000141
9	1	0	-1.489484	-1.710527	0.000319
10	1	0	-1.560999	1.604774	0.000250

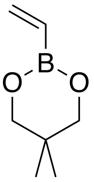

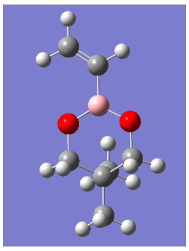

Center Number	Atomic Number	Atomic Type	Coordinates (Angstroms) X	Y	Z
1	6	0	3.633184	-0.541716	0.136821
2	6	0	2.758182	0.471750	0.096762
3	1	0	4.698626	-0.394134	0.307021
4	1	0	3.308341	-1.570944	-0.001847
5	1	0	3.131012	1.487274	0.240068
6	5	0	1.230184	0.277913	-0.148802
7	8	0	0.728276	-0.988438	-0.313514
8	8	0	0.430711	1.392476	-0.174919
9	6	0	-1.539353	-0.082623	0.065680
10	6	0	-0.971128	1.265233	-0.413164
11	6	0	-0.663655	-1.183253	-0.559257
12	1	0	-0.825396	-1.220090	-1.646226
13	1	0	-0.930018	-2.165517	-0.151320
14	6	0	-1.502427	-0.169672	1.602922
15	6	0	-2.984009	-0.233991	-0.434105
16	1	0	-1.154162	1.389015	-1.490177
17	1	0	-1.463575	2.097064	0.104186
18	1	0	-3.042131	-0.176231	-1.527827
19	1	0	-3.405802	-1.197882	-0.125270

20	1	0	-3.625318	0.553747	-0.021119
21	1	0	-1.895540	-1.134469	1.944602
22	1	0	-0.486869	-0.065292	1.996158
23	1	0	-2.116749	0.621216	2.049275

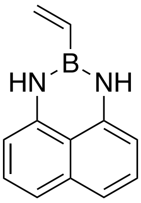

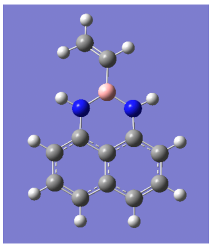

Center Number	Atomic Number	Atomic Type	Coordinates (Angstroms) X	Y	Z
1	6	0	0.996741	-2.412820	0.010688
2	1	0	0.574747	-3.415183	0.005802
3	6	0	0.153720	-1.312112	-0.017252
4	6	0	2.394441	-2.231948	0.044862
5	6	0	0.701710	0.008755	-0.010160
6	6	0	2.950660	-0.971738	0.050507
7	6	0	2.121319	0.182289	0.022406
8	6	0	-0.148879	1.158299	-0.037994
9	6	0	2.649326	1.501736	0.024702
10	6	0	0.405894	2.429541	-0.036185
11	6	0	1.805930	2.590593	-0.004806
12	7	0	-1.236679	-1.442467	-0.052425
13	1	0	3.037238	-3.108305	0.066674
14	1	0	4.029202	-0.842192	0.076623
15	7	0	-1.529631	0.951725	-0.069621
16	1	0	3.727323	1.635871	0.049212
17	1	0	-0.244423	3.301014	-0.058203
18	1	0	2.218907	3.596159	-0.003617
19	5	0	-2.139607	-0.338082	-0.070269
20	1	0	-2.088697	1.792915	-0.122748

21	1	0	-1.580037	-2.394107	-0.038886
22	6	0	-3.684746	-0.558943	-0.085486
23	1	0	-4.055673	-1.559788	-0.322727
24	6	0	-4.604573	0.375063	0.191499
25	1	0	-4.332200	1.395674	0.459522
26	1	0	-5.673446	0.171260	0.177325

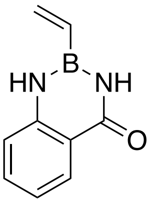

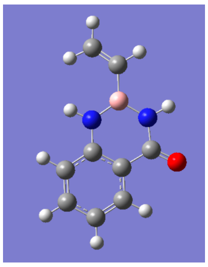

Center Number	Atomic Number	Atomic Type	Coordinates (Angstroms) X	Y	Z
1	6	0	-0.481348	-0.809183	-0.039787
2	7	0	0.896337	-0.989517	-0.081393
3	6	0	-1.007557	0.501681	0.007543
4	6	0	-1.363384	-1.903552	-0.046430
5	6	0	-2.735914	-1.695000	-0.004385
6	6	0	-2.396119	0.688705	0.049917
7	6	0	-3.261960	-0.396283	0.044760
8	1	0	-0.961002	-2.913620	-0.083417
9	1	0	-3.404449	-2.551903	-0.009507
10	6	0	-0.122671	1.691661	0.009600
11	1	0	-2.762184	1.709713	0.085723
12	1	0	-4.336230	-0.242157	0.077817
13	7	0	1.240062	1.401810	-0.037850
14	8	0	-0.532215	2.845725	0.049739
15	5	0	1.825255	0.092774	-0.076366
16	1	0	1.807350	2.242769	-0.023772
17	1	0	1.210692	-1.948624	-0.149025

18	6	0	3.371639	-0.107586	-0.106245
19	6	0	4.007842	-1.248943	0.190702
20	1	0	3.994055	0.751692	-0.367881
21	1	0	3.473475	-2.151533	0.486779
22	1	0	5.091783	-1.341070	0.167857

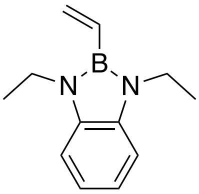

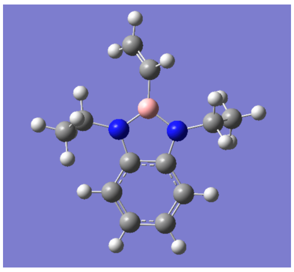

Center Number	Atomic Number	Atomic Type	Coordinates (Angstroms) X	Y	Z
1	6	0	0.746986	-0.801845	-0.169721
2	7	0	-0.638703	-0.987022	-0.200017
3	6	0	1.017719	0.546382	0.170883
4	6	0	1.789494	-1.692968	-0.419121
5	6	0	3.104727	-1.222993	-0.329406
6	6	0	2.329848	1.008652	0.256959
7	6	0	3.371273	0.108266	0.005570
8	1	0	1.592074	-2.728291	-0.680605
9	1	0	3.927611	-1.905267	-0.523851
10	7	0	-0.197134	1.203388	0.376654
11	1	0	2.544919	2.041950	0.513019
12	1	0	4.399693	0.452653	0.070289
13	6	0	-0.276500	2.626998	0.671441
14	5	0	-1.267937	0.267836	0.134655
15	6	0	-1.235105	-2.302907	-0.394560
16	6	0	-2.788646	0.598823	0.238107
17	6	0	-3.746715	0.203243	-0.612875
18	1	0	-3.109306	1.243045	1.062022

19	1	0	-3.528793	-0.411514	-1.484420
20	1	0	-4.790152	0.489455	-0.491455
21	1	0	-1.251949	2.819552	1.129141
22	1	0	0.480502	2.875905	1.426556
23	6	0	-0.103032	3.516844	-0.564826
24	1	0	0.861850	3.336786	-1.049869
25	1	0	-0.892882	3.314557	-1.295886
26	1	0	-0.154012	4.575662	-0.285055
27	1	0	-2.286834	-2.157347	-0.649765
28	1	0	-0.760475	-2.785221	-1.259351
29	6	0	-1.129743	-3.206670	0.839718
30	1	0	-0.087600	-3.359126	1.137503
31	1	0	-1.662020	-2.760141	1.686365
32	1	0	-1.573906	-4.187360	0.632609

Energy Diagram Investigation for the Chain-Growth Reaction of VBaam and VBpin: The Gaussian 16C.01 program package[8] was used for all computations. DFT calculations were applied for the optimization of the structures and vibrational analysis at the (U)CAM-B3LYP/6-31G(d) level. The nature of the optimized stationary points was proven by the presence of either 0 (minimum) or 1 (transition state) imaginary vibrational frequency. Cartesian coordinates of optimized structures and sums of electronic and thermal energies are shown for each species.

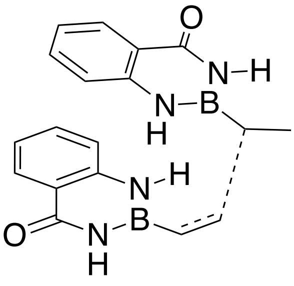

(Transition state)

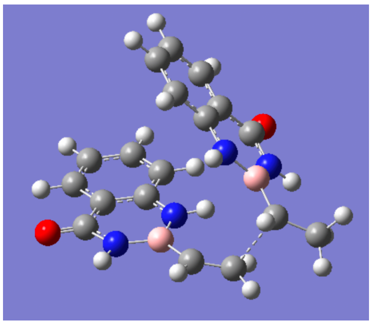

Center Number	Atomic Number	Atomic Type	Coordinates (Angstroms) X	Y	Z
1	6	0	1.356160	1.454921	1.286214
2	7	0	2.169271	0.331273	1.299222
3	6	0	1.082464	2.095892	0.066487
4	6	0	0.800752	1.966109	2.464711
5	6	0	-0.006417	3.088249	2.422576
6	6	0	0.263411	3.226735	0.044691
7	6	0	-0.280362	3.728400	1.211545
8	1	0	1.007253	1.471704	3.409999
9	1	0	-0.431768	3.470803	3.345325
10	6	0	1.635299	1.592461	-1.206657
11	1	0	0.073226	3.688485	-0.917731
12	1	0	-0.916816	4.606167	1.188628
13	7	0	2.450845	0.477355	-1.092023
14	8	0	1.390392	2.088579	-2.296761
15	1	0	2.839902	0.202675	-1.986460
16	5	0	2.763781	-0.216408	0.130374
17	1	0	2.313530	-0.095181	2.203744

18	6	0	3.627076	-1.490958	0.160072
19	1	0	3.858495	-1.907414	1.140198
20	6	0	2.039713	-3.016329	-0.352361
21	6	0	4.646267	-1.809739	-0.897001
22	1	0	4.295384	-1.566525	-1.906323
23	1	0	5.566664	-1.231691	-0.734095
24	1	0	4.933204	-2.866483	-0.897242
25	6	0	0.971587	-3.007162	0.505976
26	1	0	1.941892	-2.600235	-1.352422
27	1	0	2.827190	-3.756390	-0.245782
28	1	0	1.070468	-3.609469	1.410564
29	5	0	-0.352698	-2.233600	0.282750
30	7	0	-0.509233	-1.198670	-0.677586
31	7	0	-1.525955	-2.551924	1.041419
32	1	0	0.272676	-0.862905	-1.223210
33	6	0	-2.772337	-1.964091	0.895128
34	1	0	-1.532782	-3.284104	1.741338
35	8	0	-3.725728	-2.312257	1.572527
36	6	0	-2.839612	-0.900214	-0.129984
37	6	0	-1.711774	-0.533438	-0.882122
38	6	0	-4.054808	-0.244825	-0.338327
39	6	0	-4.160867	0.766060	-1.274513
40	6	0	-1.819429	0.498672	-1.822842
41	6	0	-3.032545	1.135717	-2.011210
42	1	0	-4.901245	-0.559822	0.262012
43	1	0	-5.107668	1.271429	-1.432826
44	1	0	-0.943294	0.808135	-2.386260
45	1	0	-3.100997	1.935289	-2.742714

Sum of electronic and thermal energies= -1115.725752

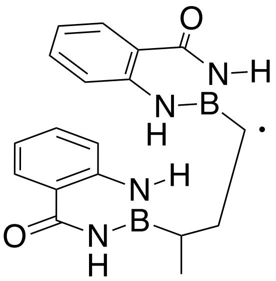

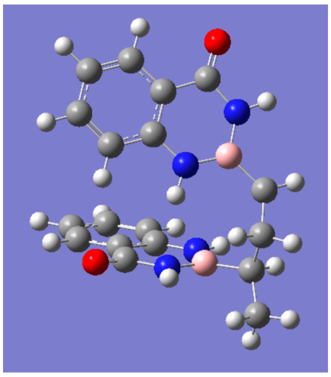

Center Number	Atomic Number	Atomic Type	Coordinates (Angstroms) X	Y	Z
1	6	0	-2.180405	-1.080174	1.109205
2	7	0	-2.345368	0.300015	1.143630
3	6	0	-1.958678	-1.717528	-0.122098
4	6	0	-2.224403	-1.847510	2.277617
5	6	0	-2.050810	-3.218473	2.214439
6	6	0	-1.783591	-3.102309	-0.165427
7	6	0	-1.829950	-3.856314	0.991576
8	1	0	-2.392858	-1.357360	3.232582
9	1	0	-2.085965	-3.800709	3.130096
10	6	0	-1.898030	-0.952047	-1.382975
11	1	0	-1.610670	-3.555335	-1.135305
12	1	0	-1.693250	-4.931465	0.952626
13	7	0	-2.114886	0.412458	-1.248442
14	8	0	-1.660564	-1.451147	-2.472120
15	1	0	-2.123594	0.883782	-2.146217
16	5	0	-2.315820	1.115210	-0.012362
17	1	0	-2.458404	0.705168	2.062471
18	6	0	-2.440485	2.690962	0.021819
19	1	0	-2.632032	3.011887	1.055163
20	6	0	-1.101364	3.347267	-0.426485
21	6	0	-3.597349	3.196136	-0.853597
22	1	0	-3.451478	2.931063	-1.907402
23	1	0	-4.556788	2.771969	-0.541050
24	1	0	-3.682250	4.287193	-0.804967

25	6	0	0.043028	3.098754	0.506502
26	1	0	-0.858782	2.991883	-1.437334
27	1	0	-1.271357	4.428673	-0.521164
28	1	0	0.058776	3.740237	1.389032
29	5	0	1.141365	2.036609	0.347721
30	7	0	1.085614	0.995477	-0.621164
31	7	0	2.306095	2.028029	1.188475
32	1	0	0.282508	0.880407	-1.223587
33	6	0	3.351815	1.123263	1.118563
34	1	0	2.461964	2.732594	1.899246
35	8	0	4.318157	1.203332	1.859773
36	6	0	3.193527	0.068183	0.092792
37	6	0	2.064151	0.018553	-0.740418
38	6	0	4.187502	-0.904259	-0.030974
39	6	0	4.069820	-1.920545	-0.960398
40	6	0	1.941001	-1.019235	-1.673273
41	6	0	2.936558	-1.973671	-1.776422
42	1	0	5.044492	-0.829295	0.629356
43	1	0	4.845460	-2.673441	-1.052069
44	1	0	1.052937	-1.077881	-2.297543
45	1	0	2.828618	-2.774137	-2.502118

Sum of electronic and thermal energies = -1115.766086

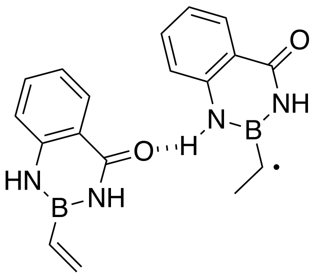

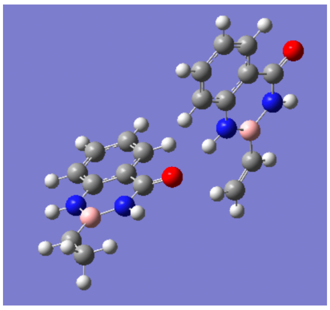

Center Number	Atomic Number	Atomic Type	Coordinates (Angstroms) X	Y	Z

1	6	0	-3.523906	-1.033861	-0.929486
2	7	0	-4.559427	-0.175115	-0.602834
3	6	0	-2.238184	-0.801226	-0.407883
4	6	0	-3.732081	-2.132777	-1.772043
5	6	0	-2.683062	-2.976018	-2.086103
6	6	0	-1.190834	-1.666323	-0.739660
7	6	0	-1.404164	-2.748151	-1.571839
8	1	0	-4.724663	-2.313767	-2.175358
9	1	0	-2.860115	-3.823556	-2.741101
10	6	0	-1.979570	0.344683	0.482884
11	1	0	-0.207819	-1.467659	-0.329107
12	1	0	-0.585965	-3.413611	-1.823651
13	7	0	-3.062566	1.145216	0.750406
14	8	0	-0.879047	0.596388	0.974526
15	1	0	-2.823260	1.912581	1.366989
16	5	0	-4.399012	0.956669	0.246370
17	1	0	-5.457237	-0.396980	-1.010319
18	6	0	-5.561980	1.887942	0.587242
19	1	0	-6.545614	1.676586	0.166182
20	6	0	0.701093	-2.141516	2.643897
21	6	0	-5.505179	3.095532	1.460110
22	1	0	-5.813697	3.997369	0.913608
23	1	0	-6.196326	3.006427	2.309563
24	1	0	-4.508450	3.286699	1.868981
25	6	0	1.946376	-2.245399	2.175251
26	1	0	0.068874	-1.285205	2.418911
27	1	0	0.254278	-2.904985	3.276799
28	1	0	2.512029	-3.135866	2.457273
29	5	0	2.652272	-1.185883	1.274922
30	7	0	1.993867	-0.055035	0.731298
31	7	0	4.040193	-1.319929	0.941610
32	1	0	1.005627	0.129309	0.891123
33	6	0	4.774943	-0.445735	0.158182
34	1	0	4.602717	-2.091994	1.278596
35	8	0	5.958122	-0.635320	-0.077036

36	6	0	4.011967	0.712014	-0.354430
37	6	0	2.649264	0.878311	-0.055637
38	6	0	4.669534	1.653199	-1.149731
39	6	0	3.995926	2.751450	-1.648827
40	6	0	1.970189	1.995336	-0.563452
41	6	0	2.640177	2.914966	-1.348191
42	1	0	5.721194	1.485383	-1.355096
43	1	0	4.511827	3.479728	-2.265727
44	1	0	0.918451	2.120639	-0.325077
45	1	0	2.101732	3.775902	-1.734017

Sum of electronic and thermal energies = -1115.746918

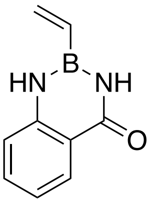

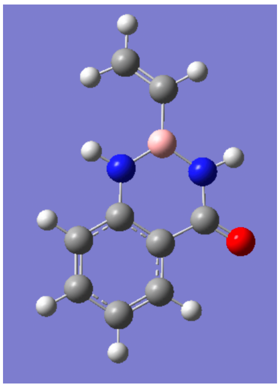

Center Number	Atomic Number	Atomic Type	Coordinates (Angstroms) X	Y	Z
1	6	0	3.976308	-1.239990	0.237066
2	6	0	3.360807	-0.115913	-0.131541
3	1	0	3.426162	-2.107762	0.597211
4	1	0	5.057254	-1.352004	0.215281
5	1	0	3.989963	0.715410	-0.453312
6	5	0	1.817425	0.092288	-0.095324
7	7	0	0.895311	-0.986930	-0.101411
8	7	0	1.238392	1.398585	-0.046010
9	1	0	1.210420	-1.943245	-0.182937

10	6	0	-0.117347	1.683860	0.011359
11	1	0	1.804752	2.238376	-0.028767
12	8	0	-0.528816	2.830394	0.060978
13	6	0	-1.001368	0.496812	0.008325
14	6	0	-0.479754	-0.805639	-0.049455
15	6	0	-2.382977	0.688063	0.060861
16	6	0	-3.248027	-0.390309	0.055280
17	6	0	-1.359228	-1.894283	-0.056573
18	6	0	-2.725578	-1.684159	-0.004583
19	1	0	-2.743745	1.709824	0.104821
20	1	0	-4.320695	-0.234826	0.096222
21	1	0	-0.958698	-2.903510	-0.101941
22	1	0	-3.394954	-2.539100	-0.010267

Sum of electronic and thermal energies = -557.583104

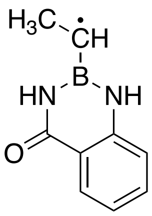

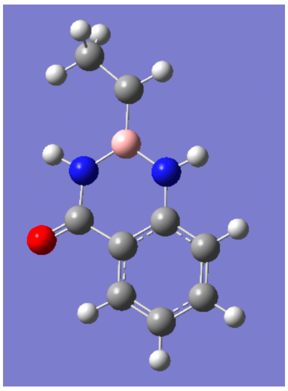

Center Number	Atomic Number	Atomic Type	Coordinates (Angstroms) X	Y	Z
1	6	0	0.764988	-0.857395	0.000117
2	7	0	-0.537479	-1.329438	0.000188
3	6	0	0.996381	0.528302	-0.000078
4	6	0	1.858785	-1.731881	0.000234
5	6	0	3.148651	-1.232470	0.000158
6	6	0	2.305208	1.012121	-0.000151

7	6	0	3.381956	0.144630	-0.000035
8	1	0	1.683677	-2.804487	0.000384
9	1	0	3.985907	-1.923938	0.000250
10	6	0	-0.121936	1.498968	-0.000208
11	1	0	2.438400	2.088396	-0.000302
12	1	0	4.396740	0.527690	-0.000093
13	7	0	-1.384391	0.932900	-0.000123
14	8	0	0.038912	2.708495	-0.000377
15	1	0	-2.111933	1.637259	-0.000219
16	5	0	-1.678139	-0.472743	0.000074
17	1	0	-0.640209	-2.334495	0.000329
18	6	0	-3.102411	-1.034076	0.000157
19	1	0	-3.243238	-2.115855	0.000308
20	6	0	-4.366489	-0.242661	0.000051
21	1	0	-4.985370	-0.477371	-0.877160
22	1	0	-4.985367	-0.477131	0.877329
23	1	0	-4.196911	0.838230	-0.000097

Sum of electronic and thermal energies = -558.150596

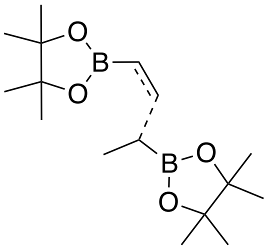

(Transition state)

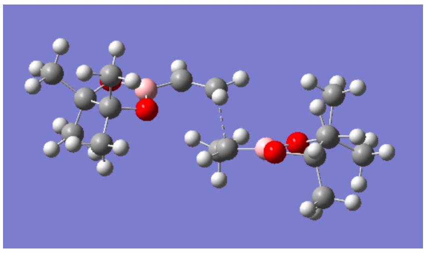

Center Number	Atomic Number	Atomic Type	Coordinates (Angstroms) X	Y	Z
1	6	0	0.794450	-2.612608	1.141595
2	6	0	0.902058	-1.140656	0.881882

3	1	0	1.022985	-2.834333	2.194010
4	1	0	-0.214099	-2.991275	0.947059
5	1	0	1.504750	-3.180719	0.533698
6	1	0	0.139613	-0.521920	1.349691
7	5	0	2.240859	-0.481289	0.525407
8	6	0	0.035310	-0.800409	-1.185749
9	1	0	0.134314	0.269056	-1.026854
10	1	0	0.875181	-1.286144	-1.677040
11	6	0	-1.203333	-1.381872	-1.174143
12	5	0	-2.445952	-0.640136	-0.652883
13	1	0	-1.291465	-2.418437	-1.494254
14	8	0	-2.390368	0.606987	-0.076319
15	8	0	-3.723393	-1.139625	-0.698985
16	6	0	-3.746818	1.076838	0.073126
17	6	0	-4.563441	-0.261390	0.080354
18	6	0	-5.932205	-0.187123	-0.578555
19	6	0	-4.045247	1.957298	-1.139590
20	6	0	-3.838504	1.897554	1.350275
21	6	0	-4.682615	-0.885775	1.469915
22	8	0	2.436237	0.877847	0.454319
23	8	0	3.367295	-1.190436	0.176738
24	6	0	3.848306	1.104834	0.262408
25	6	0	4.324348	-0.252732	-0.360804
26	6	0	5.720885	-0.696960	0.046041
27	6	0	4.443974	1.373735	1.643730
28	6	0	4.027600	2.323827	-0.629425
29	6	0	4.181486	-0.299017	-1.881559
30	1	0	3.184190	0.021629	-2.195425
31	1	0	4.921376	0.335329	-2.378035
32	1	0	4.328031	-1.328640	-2.218207
33	1	0	3.484547	2.216814	-1.569661
34	1	0	3.643024	3.210074	-0.117504
35	1	0	5.086014	2.493006	-0.852602
36	1	0	3.915777	2.216669	2.096637
37	1	0	4.324427	0.507944	2.300857
38	1	0	5.507545	1.622314	1.585767

39	1	0	6.473538	0.024600	-0.287880
40	1	0	5.805025	-0.814996	1.127383
41	1	0	5.946917	-1.661399	-0.416875
42	1	0	-3.300989	2.756440	-1.186104
43	1	0	-3.983827	1.384005	-2.068687
44	1	0	-5.037985	2.412085	-1.076230
45	1	0	-3.487304	1.333564	2.215697
46	1	0	-3.215756	2.791188	1.255335
47	1	0	-4.868559	2.219180	1.535253
48	1	0	-3.707041	-0.947589	1.959893
49	1	0	-5.359770	-0.316936	2.113516
50	1	0	-5.074123	-1.901134	1.367569
51	1	0	-6.581021	0.521186	-0.053175
52	1	0	-5.858197	0.115415	-1.624174
53	1	0	-6.407342	-1.171299	-0.545317

Sum of electronic and thermal energies = -978.118034

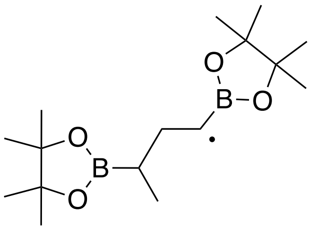

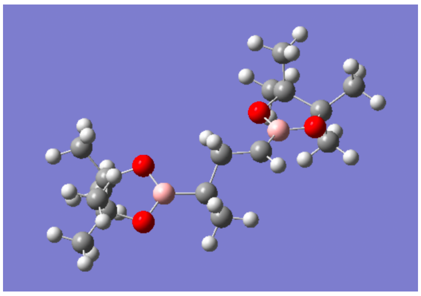

Center Number	Atomic Number	Atomic Type	Coordinates (Angstroms) X	Y	Z
1	6	0	-1.365441	2.958753	0.086021
2	6	0	-1.087179	1.456364	0.218089
3	1	0	-2.146798	3.274249	0.782715
4	1	0	-0.473678	3.560807	0.285635
5	1	0	-1.711782	3.206013	-0.924515
6	1	0	-0.687087	1.277790	1.229302

7	5	0	-2.407970	0.606085	0.131768
8	6	0	-0.023870	0.941615	-0.785883
9	1	0	0.037947	-0.147883	-0.715952
10	1	0	-0.389252	1.165297	-1.802053
11	6	0	1.332219	1.540935	-0.609071
12	5	0	2.593964	0.730099	-0.317473
13	1	0	1.407984	2.623389	-0.698859
14	8	0	2.599836	-0.637103	-0.153926
15	8	0	3.850340	1.276422	-0.189000
16	6	0	3.976744	-1.065411	-0.129152
17	6	0	4.725323	0.241622	0.304706
18	6	0	6.102130	0.433332	-0.312955
19	6	0	4.324984	-1.514574	-1.547850
20	6	0	4.105083	-2.234717	0.835169
21	6	0	4.802233	0.414627	1.821284
22	8	0	-2.457925	-0.687066	-0.317727
23	8	0	-3.637407	1.071304	0.519688
24	6	0	-3.769574	-1.211080	-0.012254
25	6	0	-4.626339	0.094684	0.123163
26	6	0	-5.715166	0.047659	1.183904
27	6	0	-3.637513	-1.985415	1.298006
28	6	0	-4.190582	-2.148635	-1.133112
29	6	0	-5.207999	0.576110	-1.204912
30	1	0	-4.439254	0.619205	-1.981350
31	1	0	-6.016557	-0.073369	-1.552177
32	1	0	-5.608021	1.584240	-1.069724
33	1	0	-4.149917	-1.656115	-2.105762
34	1	0	-3.515859	-3.008376	-1.162254
35	1	0	-5.207451	-2.520108	-0.970185
36	1	0	-2.870096	-2.754180	1.177160
37	1	0	-3.331273	-1.328863	2.117091
38	1	0	-4.575935	-2.473383	1.576256
39	1	0	-6.453884	-0.726122	0.951275
40	1	0	-5.302999	-0.149237	2.174686
41	1	0	-6.232216	1.010345	1.219050
42	1	0	3.620850	-2.292727	-1.853488

43	1	0	4.240627	-0.685607	-2.255885
44	1	0	5.338470	-1.921690	-1.607589
45	1	0	3.721979	-1.982002	1.825004
46	1	0	3.529205	-3.084077	0.457678
47	1	0	5.149434	-2.548729	0.932548
48	1	0	3.821028	0.278404	2.284259
49	1	0	5.502679	-0.292070	2.275634
50	1	0	5.142227	1.429524	2.042954
51	1	0	6.780855	-0.372034	-0.013791
52	1	0	6.052277	0.462329	-1.402484
53	1	0	6.527454	1.380380	0.030149

Sum of electronic and thermal energies = -978.157463

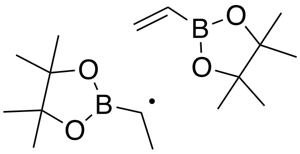

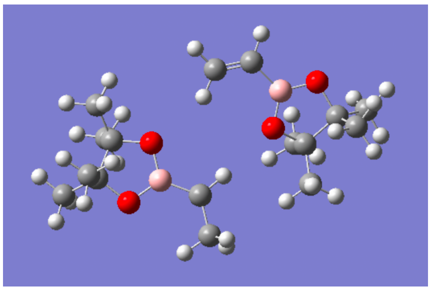

Center Number	Atomic Number	Atomic Type	Coordinates (Angstroms) X	Y	Z
1	6	0	-0.906192	3.146114	-0.653283
2	6	0	-0.917636	1.675364	-0.408320
3	1	0	-0.302045	3.675493	0.098061
4	1	0	-0.449355	3.385755	-1.624484
5	1	0	-1.912501	3.572283	-0.638115
6	1	0	0.046355	1.168974	-0.401931
7	5	0	-2.189631	0.864087	-0.177749
8	6	0	0.499212	-2.618726	-0.648820
9	1	0	-0.076574	-1.740492	-0.368535
10	1	0	-0.083345	-3.504020	-0.897819

11	6	0	1.833323	-2.619705	-0.683764
12	5	0	2.689720	-1.380943	-0.329376
13	1	0	2.348143	-3.538180	-0.964592
14	8	0	2.183447	-0.125621	-0.089808
15	8	0	4.054530	-1.422979	-0.213129
16	6	0	3.254819	0.678168	0.454220
17	6	0	4.527628	-0.065917	-0.075900
18	6	0	5.719525	-0.059863	0.868695
19	6	0	3.114669	0.626564	1.974645
20	6	0	3.094710	2.108199	-0.037886
21	6	0	4.957371	0.392494	-1.468623
22	8	0	-2.205739	-0.502167	0.000812
23	8	0	-3.452565	1.409180	-0.124964
24	6	0	-3.536868	-0.872345	0.416755
25	6	0	-4.397843	0.319225	-0.127344
26	6	0	-5.586868	0.705102	0.739121
27	6	0	-3.516653	-0.952380	1.942639
28	6	0	-3.870369	-2.233603	-0.174026
29	6	0	-4.844503	0.124972	-1.575855
30	1	0	-4.002362	-0.155499	-2.214411
31	1	0	-5.618059	-0.643462	-1.662334
32	1	0	-5.251474	1.068560	-1.948379
33	1	0	-3.744557	-2.242137	-1.257770
34	1	0	-3.202644	-2.989037	0.249438
35	1	0	-4.900080	-2.520903	0.062211
36	1	0	-2.741703	-1.660142	2.247968
37	1	0	-3.280670	0.018974	2.385793
38	1	0	-4.475155	-1.294325	2.343554
39	1	0	-6.299183	-0.122693	0.816745
40	1	0	-5.274281	0.993070	1.743990
41	1	0	-6.104693	1.556937	0.289954
42	1	0	2.114653	0.972957	2.247870
43	1	0	3.232093	-0.394236	2.348413
44	1	0	3.850485	1.265873	2.470802
45	1	0	3.027582	2.153074	-1.126009
46	1	0	2.179121	2.539808	0.375829

47	1	0	3.936952	2.727601	0.287097
48	1	0	4.111914	0.395290	-2.161998
49	1	0	5.394948	1.394679	-1.448637
50	1	0	5.706803	-0.304212	-1.852788
51	1	0	6.068101	0.961773	1.051359
52	1	0	5.475380	-0.522714	1.826018
53	1	0	6.542375	-0.624047	0.421520

Sum of electronic and thermal energies = -978.130241

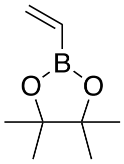

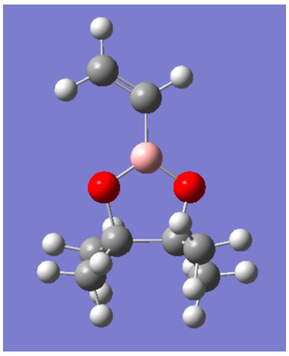

Center Number	Atomic Number	Atomic Type	Coordinates (Angstroms) X	Y	Z
1	6	0	3.662802	0.288870	0.121145
2	1	0	3.367573	1.299559	0.392056
3	1	0	4.732257	0.094734	0.081136
4	6	0	2.749255	-0.646279	-0.142500
5	5	0	1.230024	-0.351285	-0.075512
6	1	0	3.083662	-1.647372	-0.411373
7	8	0	0.709187	0.858671	0.305355
8	8	0	0.262749	-1.267542	-0.390872
9	6	0	-0.706319	0.831395	0.017897
10	6	0	-1.011993	-0.706242	-0.005145
11	6	0	-2.060252	-1.142234	-1.016970
12	6	0	-0.892495	1.503437	-1.341516

13	6	0	-1.435349	1.619262	1.095178
14	6	0	-1.342387	-1.276263	1.373239
15	1	0	-0.468451	2.509887	-1.298156
16	1	0	-0.373147	0.952073	-2.130113
17	1	0	-1.949182	1.584609	-1.611738
18	1	0	-1.198736	1.248367	2.093531
19	1	0	-1.135232	2.669433	1.045601
20	1	0	-2.519238	1.568847	0.949649
21	1	0	-0.597578	-0.974870	2.114855
22	1	0	-2.328452	-0.953394	1.719114
23	1	0	-1.337216	-2.367619	1.313779
24	1	0	-3.030003	-0.684716	-0.796046
25	1	0	-1.771542	-0.875700	-2.034758
26	1	0	-2.179897	-2.228117	-0.973804

Sum of electronic and thermal energies = -488.781173

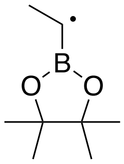

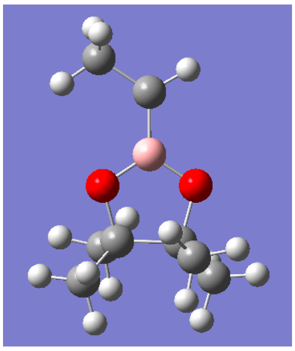

Center Number	Atomic Number	Atomic Type	Coordinates (Angstroms) X	Y	Z
1	6	0	3.737104	0.270355	0.131081
2	6	0	2.659357	-0.711087	-0.180144
3	1	0	4.379811	-0.085230	0.949145
4	1	0	4.404575	0.419520	-0.729817
5	1	0	3.329872	1.243019	0.417483

6	1	0	2.980807	-1.707971	-0.477347
7	5	0	1.167004	-0.391627	-0.102940
8	8	0	0.168060	-1.282223	-0.417516
9	8	0	0.675019	0.831412	0.292648
10	6	0	-1.082754	-0.694108	-0.002703
11	6	0	-0.742366	0.835986	0.021484
12	6	0	-1.442644	1.637054	1.108358
13	6	0	-1.399475	-1.261360	1.380553
14	6	0	-2.162820	-1.100187	-0.993699
15	6	0	-0.930041	1.515012	-1.334639
16	1	0	-0.433924	0.952218	-2.130114
17	1	0	-1.987784	1.621811	-1.591712
18	1	0	-0.481641	2.511073	-1.295095
19	1	0	-1.888668	-0.836349	-2.016240
20	1	0	-2.307624	-2.183130	-0.952426
21	1	0	-3.116616	-0.620532	-0.751026
22	1	0	-1.418909	-2.352390	1.317602
23	1	0	-0.633944	-0.978552	2.108335
24	1	0	-2.371404	-0.918585	1.746922
25	1	0	-2.529010	1.610194	0.975235
26	1	0	-1.202687	1.258792	2.103195
27	1	0	-1.120669	2.680750	1.057759

Sum of electronic and thermal energies = -489.347482

12 References

(1) Cain, D. L.; McLaughlin, C.; Molloy, J. J.; Carpenter-Warren, C.; Anderson, N. A.; Watson, A. J. B. *Synlett* **2019**, *30*, 787−791.

(2) Morrill, C.; Grubbs, R. H. *J. Org. Chem.* **2003**, *68*, 6031−6034.

(3) Ling, X.; Schaeffer, N.; Roland, S.; Pileni, M.-P. *Langmuir* **2013**, *29*, 12647−12656.

(4) Mayo, F. R.; Lewis, F. M. *J. Am. Chem. Soc.* **1944**, *66*, 1594−1601.

(5) (a) Van Herk, A. M.; Dröge, T. *Macromol. Theory Simul.* **1997**, *6*, 1263–1276. (b) van de Wouw, H. L.; Awuyah, E. C.; Baris, J. I.; Klausen, R. S. *Macromolecules* **2018**, *51*, 6359–6368.

(6) Zhang, H.; Nomura, K. *J. Am. Chem. Soc.* **2005**, *127*, 9364−9365.

(7) Gaussian 16, Revision A.03, M. J. Frisch, G. W. Trucks, H. B. Schlegel, G. E. Scuseria, M. A. Robb, J. R. Cheeseman, G. Scalmani, V. Barone, G. A. Petersson, H. Nakatsuji, X. Li, M. Caricato, A. V. Marenich, J. Bloino, B. G. Janesko, R. Gomperts, B. Mennucci, H. P. Hratchian, J. V. Ortiz, A. F. Izmaylov, J. L. Sonnenberg, D. Williams-Young, F. Ding, F. Lipparini, F. Egidi, J. Goings, B. Peng, A. Petrone, T. Henderson, D. Ranasinghe, V. G. Zakrzewski, J. Gao, N. Rega, G. Zheng, W. Liang, M. Hada, M. Ehara, K. Toyota, R. Fukuda, J. Hasegawa, M. Ishida, T. Nakajima, Y. Honda, O. Kitao, H. Nakai, T. Vreven, K. Throssell, J. A. Montgomery, Jr., J. E. Peralta, F. Ogliaro, M. J. Bearpark, J. J. Heyd, E. N. Brothers, K. N. Kudin, V. N. Staroverov, T. A. Keith, R. Kobayashi, J. Normand, K. Raghavachari, A. P. Rendell, J. C. Burant, S. S. Iyengar, J. Tomasi, M. Cossi, J. M. Millam, M. Klene, C. Adamo, R. Cammi, J. W. Ochterski, R. L. Martin, K. Morokuma, O. Farkas, J. B. Foresman, and D. J. Fox, Gaussian, Inc., Wallingford CT, 2016.

(8) Gaussian 16, Revision C.01, M. J. Frisch, G. W. Trucks, H. B. Schlegel, G. E. Scuseria, M. A. Robb, J. R. Cheeseman, G. Scalmani, V. Barone, G. A. Petersson, H. Nakatsuji, X. Li, M. Caricato, A. V. Marenich, J. Bloino, B. G. Janesko, R. Gomperts, B. Mennucci, H. P. Hratchian, J. V. Ortiz, A. F. Izmaylov, J. L. Sonnenberg, D. Williams-Young, F. Ding, F. Lipparini, F. Egidi, J. Goings, B. Peng, A. Petrone, T. Henderson, D. Ranasinghe, V. G. Zakrzewski, J. Gao, N. Rega, G. Zheng, W. Liang, M. Hada, M. Ehara, K. Toyota, R. Fukuda, J. Hasegawa, M. Ishida, T. Nakajima, Y. Honda, O. Kitao, H. Nakai, T. Vreven, K. Throssell, J. A. Montgomery, Jr., J. E. Peralta, F. Ogliaro, M. J. Bearpark, J. J. Heyd, E. N. Brothers, K. N. Kudin, V. N. Staroverov, T. A. Keith, R. Kobayashi, J. Normand, K. Raghavachari, A. P. Rendell, J. C. Burant, S. S. Iyengar, J. Tomasi, M. Cossi, J. M. Millam, M. Klene, C. Adamo, R. Cammi, J. W. Ochterski, R. L. Martin, K. Morokuma, O. Farkas, J. B. Foresman, and D. J. Fox, Gaussian, Inc., Wallingford CT, 2019.
